# The iGains4Gains model guides irrigation water conservation and allocation to enhance nexus gains across water, food, carbon emissions, and nature

**DOI:** 10.1088/2976-601X/adabe9

**Published:** 2025-02-06

**Authors:** Bruce Lankford, Nafn Amdar, Matthew McCartney, Tafadzwanashe Mabhaudhi

**Affiliations:** 1 School of Global Development, University of East Anglia, Norwich, United Kingdom; 2 International Water Management Institute, Amman, Jordan; 3 International Water Management Institute, Battaramulla, Sri Lanka; 4 Centre on Climate Change and Planetary Health, London School of Hygiene and Tropical Medicine, London, United Kingdom; 5 Centre for Transformative Agricultural and Food Systems, University of KwaZulu-Natal, Pietermaritzburg, South Africa; 6 Institute for Water, Environment and Health, United Nations University, Richmond, Ontario, Canada

**Keywords:** climate change, irrigated agriculture, irrigation paradox, real water savings

## Abstract

This paper introduces and applies iGain4Gains, an Excel-based model, to reveal how changes to water conservation and allocation, and irrigation technology, can produce four nexus gains. These gains are; reduced aggregate water consumption, sustained crop production, lower carbon emissions, and enhanced water availability for nature. We developed the model with limited data and hypothetical future scenarios from the Amman–Zarqa basin in Jordan. Given its significant irrigation and urban water demands and difficult decisions regarding future water allocation and nexus choices, this basin is a highly appropriate case study. The paper’s primary aim is to demonstrate the iGains4Gains nexus model rather than to build an accurate hydrological model of the basin’s water resources. The model addresses two critical questions regarding increased irrigation efficiency. First, can irrigation efficiency and other factors, such as irrigated area, be applied to achieve real water savings while maintaining crop production, ensuring greenhouse gas emission reductions, and ‘freeing’ water for nature? Second, with the insight that water conservation is a distributive/allocative act, we ask who between four paracommoners (the proprietor irrigation system, neighbouring irrigation systems, society, and nature) benefits hydrologically from changes in irrigation efficiency? Recognising nexus gains are not always linear, positive and predictable, the model reveals that achieving all four gains simultaneously is difficult, likely leading to trade-offs such as water consumption rebounds or increased carbon emissions. Demonstrated by its use at a workshop in Jordan in February 2024, iGains4Gains can be used by students, scientists and decision-makers, to explore and understand nexus trade-offs connected to changes in irrigation management. The paper concludes with recommendations for governing water and irrigated agriculture in basins where large volumes of water are withdrawn and depleted by irrigation.

## 1 Introduction

### 1.1 Nexus gains from water allocation and irrigation water conservation

The water-energy-food (WEF) nexus frames the interconnectedness of key resources in river basin systems that alter and reflect water withdrawals and consumption to meet societal priorities such as domestic water provision and food security (Taguta *et al* 2022, Shah 2023). A major economic and hydrological subset of this allocation is water for irrigated agriculture with its objective of crop production (Schultz *et al* 2005, Brauman *et al* 2013). However, the WEF nexus reminds us that trade-offs and externalities arise because of this water use. In river basins, these externalities are the large volumes of water withdrawn and consumed by irrigation (Wu *et al* 2022), the use of energy (and therefore the emitting of carbon dioxide) in water operations (Scott 2013, Tarjuelo *et al* 2015), and the reduction of water for other sectors, especially environmental flows (Harwood *et al* 2018).

**Figure 1:**
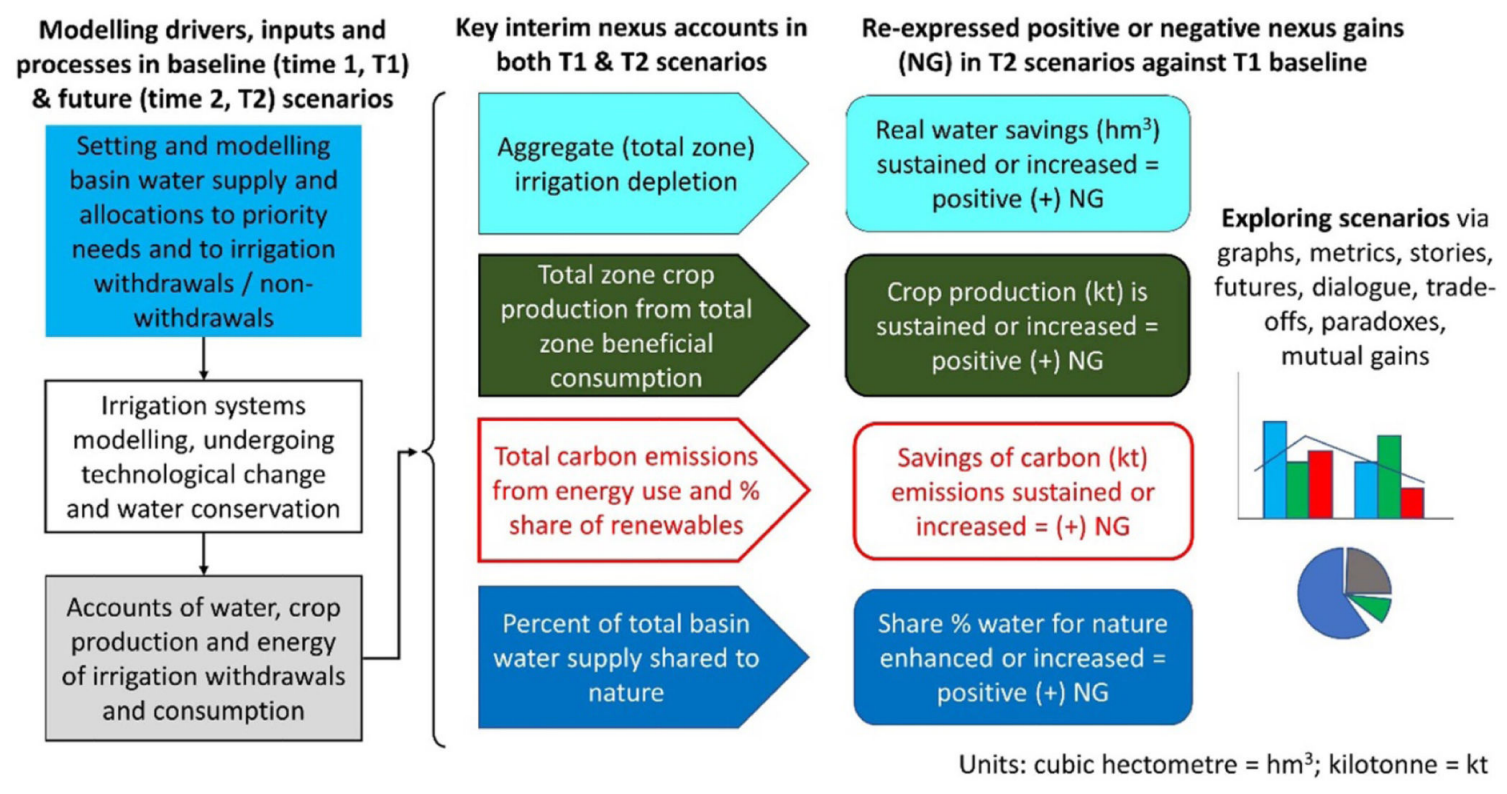
The iGains4Gains determination of four nexus gains in an irrigated river basin.

Figure 1 introduces our task and approach. This task is framed as a single question; ‘Can changes in water allocation and irrigation management in irrigated river basins deliver water-energy-food nexus gains?’ To answer this, we developed an Excel model called iGains4Gains. This model combines purposive water allocation decisions and the redistribution of water based on irrigation efficiency improvements across three zones (primary, expansion and reuse) of irrigated systems located within a river basin/aquifer to assess nexus gains. It compares selected nexus metrics in T2 (after) scenarios with the same metrics from a T1 baseline case (before). Figure 1 presents a process that starts in the top left-hand corner, where decisions about priority basin water allocation are made. These determine how much water is withdrawn into the irrigated systems of the basin. The next step applies irrigation/hydrology variables and calculations (e.g. irrigation efficiency and effective rainfall) to these withdrawals, resulting in the irrigation systems’ water, crop and energy accounts. The model then reconstitutes the different flows from the priority allocations and irrigation hydrology to derive interim basin water allocations and dispositions, including; the amount of water beneficially consumed in crop production; the energy used within, and carbon emissions from, irrigation technologies, such as sprinkler or drip; and the proportion of the water allocated to nature. These interim results are then expressed as positive or negative nexus gains.

Thus, summarising, moving from left to right in figure 1, the modeller selects user-defined (independent) water allocation and irrigation hydrology variables to derive metrics and graphs of nexus gains or reversals. User-defined selections are usually estimates guided by existing data, experience, and literature sources, including textbooks, dialogue, and new research. Some variables (e.g. total command area under irrigation) can be relatively accurately determined from satellite imagery. Other variables (e.g. irrigation efficiency) are often difficult to measure across time and space, and may be informed by experience, estimates and fieldwork.

Our paper is structured as follows. We complete the introduction by contextualising our approach within the growing nexus literature, highlighting key dimensions that underpin our interpretation and approach. In section 2, we introduce the iGains4Gains model (appendix B), with additional details provided in appendix A. Section 3 describes the case study, the Amman–Zarqa (AZ) basin, chosen for its relevance due to the significant challenges it faces from climate change, population growth and high water demand and competition. The model reveals the nexus gains and reversals associated with different water management scenarios. Section 4 presents the results of the variables selected in section 3 and sections 5 and 6 are a discussion and conclusion, respectively. Table 1 presents key terms and definitions employed in this paper. Appendix A presents the full list of terms.

### 1.2 Locating our approach within the literature on WEF nexus modelling

In the last ten years, many models addressing the WEF nexus have been produced (Taguta *et al* 2022). We locate our approach’s objective and scope in the following sub-sections by reviewing selected WEF literature and published models and methods. This, in turn, allows us to define our contribution to knowledge and to justify our methods. Although further information is given below, it is worth stating here how our approach is novel within the field of nexus modelling. iGains4Gains employs updated water accounting (Lankford 2023, Lankford and Scott 2023) to quantify how changes in purposive water allocation and irrigation management (particularly efficiency) lead to new water dispositions, giving results for real water savings, crop production, carbon emissions and water for nature.

#### 1.2.1 Changes in basin water allocations, withdrawals and consumption

Our model quantifies the nexus gains arising from changes to allocating and managing water flows within an irrigated river basin. These allocations are water for society (for domestic and industrial provisioning), irrigated agriculture and nature. With this focus on basin/aquifer water volumes and their flow pathways, we do not quantify other ways of altering crop production, food security and energy that arise outside these water volumes. This is why our nexus model excludes rainfed agriculture which was, for example, covered by Kumar *et al* (2012), or shifts in virtual water (embedded water within crops) addressed by El Gafy *et al* (2017).

#### 1.2.2 The placement of irrigation and irrigation efficiency in the WEF nexus

We focus on WEF nexus modelling where changes in irrigation hydrology are central to generating nexus outcomes. This choice is driven by the increasing competition for water in water-stressed basins (Kattel 2019) and because irrigation accounts for considerable water withdrawals and consumption across the globe (Wu *et al* 2022). It is under these circumstances, water reallocation out of irrigation without harming crop production (Pérez-Blanco *et al* 2020, Wellington *et al* 2023), raising carbon dioxide emissions (Rothausen and Conway 2011, Scott 2013) or undermining ecological flows (Batchelor *et al* 2014, Mccartney *et al* 2019) become significant WEF challenges. By placing irrigation technology, efficiency and other practices centrally in the WEF nexus, we differ from other approaches which, although they take a comprehensive and quantitative view of climate, water, energy and food (Lee *et al* 2020, Akbari Variani *et al* 2023, Wu *et al* 2023), do not model how hydrological changes within irrigation materially affect water consumption and availability, crop production, carbon emissions and water for nature. The phenomenon of WEF frameworks not being able to cover all of the many intersections of water, land, food and energy is well recognised (Simpson and Jewitt 2019).

#### 1.2.3 The diversity of nexus metrics: many or one metric?

In expressing changes within the WEF nexus, a choice arises regarding the number of metrics that describe outcomes, and that there may be a balance between too many metrics or too few or only one. In their review, Hamidov and Helming (2020) suggest that nexus models can contain many factors and variables, such as virtual water, soil salinization, soil carbon sequestration and novel production techniques found in industrial farming. Concerning a high number of variables in the composition and outputs of nexus models, examples include El Gafy *et al* (2017) and Sadeghi *et al* (2020). On the question of the validity of a single nexus index, we point to approaches that combine metrics to calculate a WEF nexus index (El Gafy *et al* 2017, Nhamo *et al* 2020, Sadeghi *et al* 2020).

While we fully accept that each nexus model will have its own objectives and internal logic, there is a discussion on the appropriateness of including too many or too few factors. In our answer to this question, the four nexus outcomes within iGains4Gains address a key debate regarding the scope for pareto-neutral carbon-free water savings derived from large areas of irrigated agriculture in a water-limited world. Thus, the results from our model iGains4Gains are expressed by four nexus gains/reversals: total water depletion, food production, carbon emissions and water for nature. These four metrics discern the behaviour of the nexus between water, food, energy and nature without being overwhelmed by too many nexus indicators or, contrastingly, being asked to judge the dynamics of the nexus via a single index. Thus, our four selected nexus metrics enable the sectoral implications of water allocation and conservation changes to be assessed. A single nexus metric would hide this diversity and the paradoxical shifts and trade-offs occurring within the nexus. Nonetheless, we can conjoin these four nexus interests as one nexus ambition. This can be expressed as a single sentence: to reduce total water consumption in irrigation to improve water allocation for nature whilst increasing or maintaining crop production and reducing carbon emissions.

#### 1.2.4 Nexus interconnectedness: post-hoc by association or causally driven?

In their literature review, Hamidov and Helming (2020) distinguish models that focus on the trade-offs and interconnectedness of the nexus, suggesting that more research on this particular aspect of the nexus should be prioritised. Furthermore, Payet-Burin *et al* (2019) identify two kinds of model interconnectedness: one where results from separate ‘single system’ models are fed into a nexus model and one where all interactions are captured within one holistic model. Our model mathematically interconnects the four nexus outcomes within one holistic, integrated model. In other words, changes to input variables cause changes in the four nexus outcomes. This interconnectedness is achieved via computations of water, energy and crop production applied to a total area of irrigation that changes depending on water allocations, irrigation hydrology and water conservation. This causality is also revealed in the inseparability of the four nexus indicators; it is impossible within iGains4Gains to boost one nexus gain without positive or negative changes occurring in at least one other nexus outcome.

Unless we have misinterpreted other nexus calculators, some designs connect nexus outcomes by association. In other words, while it appears water savings, energy and yields are connected to water management, the former three can be adjusted independently of the latter. This arises because the sub-modules in the model do not reference the dynamics of water management via common determining variables such as changing the irrigation efficiency and command area. Examples of nexus models that contain sub-models that appear not to be mathematically interconnected include Xu *et al* (2020) and Lee *et al* (2020).

#### 1.2.5 Choices regarding energy and carbon emissions

Our model iGains4Gains takes two purposive decisions on energy that directly reflect the changing volumes of water use within irrigation driven by water allocations and water conservation. First, the model only calculates energy use and greenhouse gas (GHG) emissions ascribed to irrigation water withdrawals, conveyance and distribution, which can change when irrigation technologies change or when volumes of water alter. This is akin to the approach taken by Campana *et al* (2022). Thus, our model does not calculate energy use and GHG emissions arising elsewhere as these depend on farming practices that are not tied to irrigation. The following are examples of these excluded emissions: those arising from farm mechanisation and fertiliser use (El Gafy *et al* 2017, Akbari Variani *et al* 2023), during the desalinisation of sea water (Panagopoulos 2021), during domestic and urban water purification and reticulation (Nair *et al* 2014), and when upstream irrigation affects downstream hydropower generation (Geressu *et al* 2020). Although changes in hydropower generation in an irrigated catchment will be a function of changes in total water depletion within irrigation, other non-irrigation factors are involved at this wider catchment scale. To reiterate, we are primarily interested in the pareto consequences of changes brought by water conservation within the irrigation sector, asking if water savings can be made without cutting crop production, increasing GHG emissions, and harming nature.

Second, the model re-calculates changes in energy use within irrigation as carbon dioxide emissions. Our emphasis on carbon dioxide reflects increasing concerns regarding climate change from GHGs and the need to transition to renewables (or nuclear) rapidly (Rahman *et al* 2022). In other words, a nexus model need not be concerned with measuring energy *per se* if energy is sourced from renewables at a relatively low cost.

#### 1.2.6 AWD, real water savings, scale, water reuse and water rebounds

Our model iGains4Gains is expressly designed to calculate the changes in aggregate water depletion (AWD) across the total area of an irrigated system—which itself changes via the use of return flows or forestalled losses. Calculating a change in AWD allows real water savings (or rebounds) to be assessed. Let us unpack this in more detail. Because real water savings recorded across a total irrigation system are central to the four nexus gains, we control for the possibility of rebounds in water consumption (Hamidov and Helming 2020) via either water reuse of return flows across scale, or the use of forestalled losses within scale. Building on Lankford (2023) and Lankford and Scott (2023), the iGains4Gains model accommodates across-scale and within-scale growth of irrigation areas. It does this by accounting for water demands at the field-scale and then at the farm-scale applied to three zones connected by changes to water management. These are the primary irrigation zone (usually given by the T1 first-use command area), the expansion zone (a reflection of whether in T2 the primary zone has expanded) and the reuse zone (given by the area of land irrigated from return flows from the primary and expansion zones in both T1 and T2).

Without this control and analysis of efficiency-induced growth in water depletion, misguided or conflicting attempts to save water (Molle and Tanouti 2017) will continue to result in either no impact or an increase or rebound in water consumption (Ward and Pulido-Velázquez 2008, Grafton *et al* 2018, Wheeler *et al* 2020) or run the risk of reducing crop production (Pérez-Blanco *et al* 2020). However, incorporating scale and scale effects within models that quantify irrigation water depletion is far from easy. This can mean that models that purport to capture total depletion at the catchment scale accurately do not fully connect field-level savings, farm-level water withdrawal savings, and total zone aggregate water depletion (Jägermeyr *et al* 2017, Geressu *et al* 2020, Kaune *et al* 2020, Siderius *et al* 2022).

#### 1.2.7 Water for nature as a nexus outcome

Water for nature is given prominence as a key nexus metric in iGains4Gains for several reasons. Water for nature, seen for example in environmental flows (or ecological reserve in South Africa), is a key goal and indicator of the good governance of river basins (Tickner *et al* 2020, Chen *et al* 2021). In addition, it is recognised that environmental flows connect to poverty alleviation (Poff and Matthews 2013, Matthews *et al* 2014) and to human health and well-being (Anderson *et al* 2019). The corollary also applies; environmental degradation and harm correlate to social harm and injustices (Grande *et al* 2014). Furthermore, because safeguarding environmental flows is difficult in practice, we perceive this as a critical indicator of the governance of a river basin and its ability to fairly allocate water amongst ‘at risk’ uses, such as the environment, that may only receive residual or no flows at all (Forslund *et al* 2009).

This water for nature gain could be seen as another way of expressing a ‘real water saving’ (defined as a cut in aggregate water consumption). However, the clarity of water for nature gives weight to both the purposive allocation of basin water to nature and the redistribution of irrigation water to nature as a paracommoner. Thus, our water for nature indicator responds to Grande *et al*’s (2014) identification of environmental equity as a criterion of good water management.

## 2 Methods: the iGain4Gains model

### 2.1 Introduction

**Figure 2:**
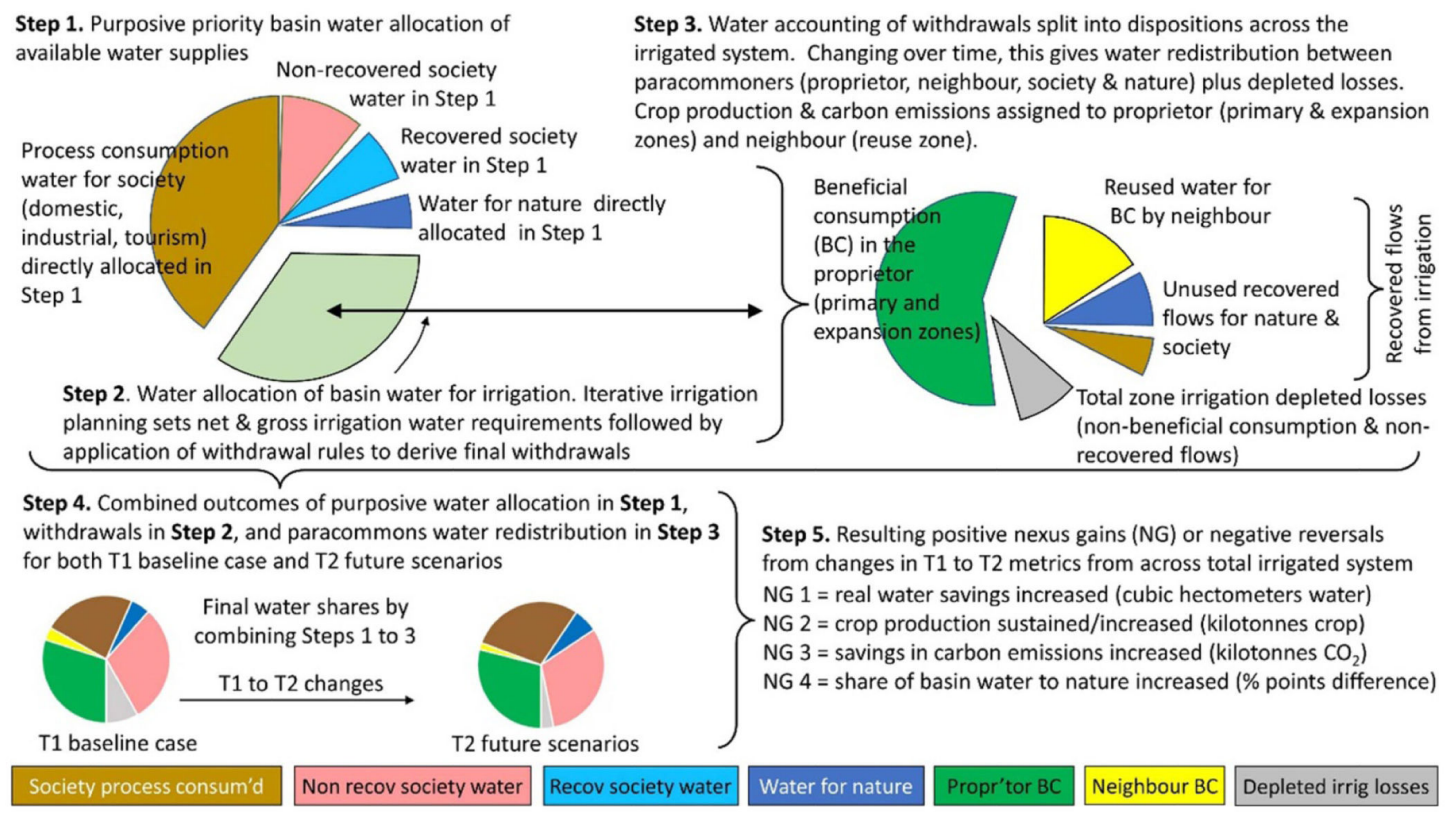
The five main steps in deriving the nexus gains of an irrigated river basin.

Figure 2 presents an overview of the iGains4Gains model broken down into five steps (the model comprises 14 stages, as explained below). The top lefthand side of the diagram presents the first step of purposive priority water allocation. These priority allocations result in water for society (domestic, industrial and tourism use) and water for nature. The second step establishes the water allocation for agriculture as a final withdrawal volume, derived from the net and gross irrigation requirements adjusted by some withdrawal rules for both T1 and T2 scenarios. In the third step, in the top right-hand side of the diagram, the volume withdrawn is fractionated by irrigation planning and practice variables into water accounting dispositions (e.g. beneficial consumption (BC), non-BC (NBC), etc) within three zones of the irrigated system (Willardson *et al* 1994, Perry 2011). These dispositions, zones of irrigation and their areal extent can then be linked to changes in per-zone and total zone crop production and carbon emissions. The fourth step, at the bottom of the diagram, is the combination of outcomes from steps 1–3, namely purposive water allocation, irrigation withdrawals and the management of irrigation water. These are calculated as changes over time from T1 to T2. For example, water for nature is derived from changes to initial purposive allocation and changes to return flows or savings within irrigation. In the fifth and final step, our model calculates how these changes generate four potential nexus outcomes expressed as gains: (1) water savings boosted (depletion is reduced across the total irrigated system); (2) crop production is sustained or boosted; (3) carbon emissions are saved (reduced), and (4) water for nature is sustained or increased.

**Figure 3:**
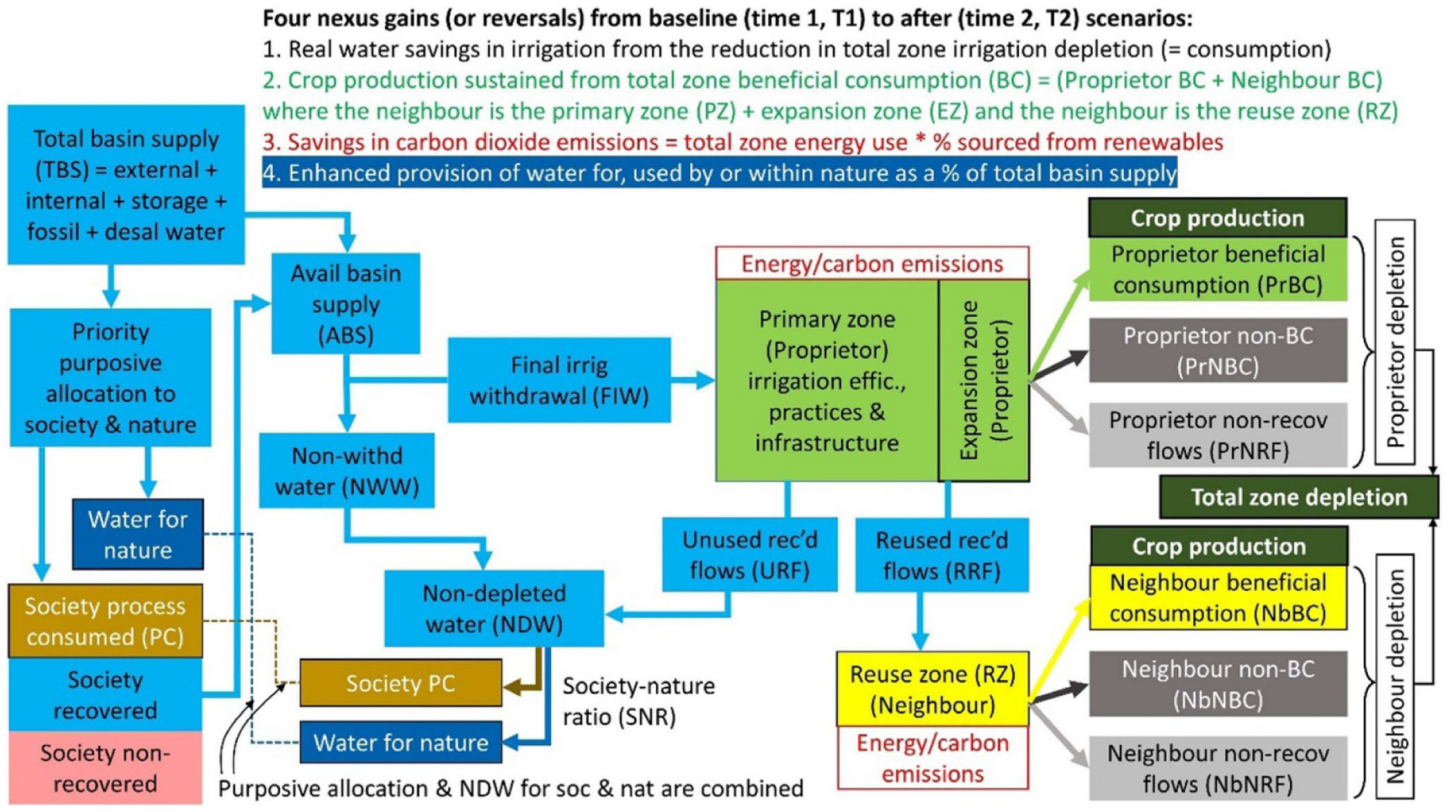
Block diagram of water flows and dispositions in the iGains4Gains model.

The iGains4Gains model can also be shown as a spatial conceptualisation of the principal flows of water from the basin supply through to different uses and dispositions. Figure 3 presents this conceptualisation as a block diagram using different colours to reflect different basin flows, uses and dispositions. The large light blue box in the top left corner is the total water supply for all uses. This is then purposively allocated to society comprising industry, tourism and domestic use, and nature (as navy blue) as priority demands. Society’s water divides into process consumed (brown), recovered (light blue) and non-recovered (pink). The remaining water (including any recovered flows (RF) from society) is available for withdrawal or non-withdrawal (in light blue) by the first-use irrigation system (the primary zone or proprietor in green). Depending on changes, the primary zone can expand into a (green) T2 expansion zone, usually using forestalled T1 losses. RF from the primary zone are reused by a reuse zone (the immediate neighbour, yellow). All unused RF (URF) from the primary zone are in light blue, and are combined with non-withdrawn water to make non-depleted water (NDW, light blue) available for use by society and nature.

The following points briefly describe some other design principles for iGains4Gains:

- From the primary, expansion and reuse zones, there are three dispositions: BC (green), NBC (dark grey) and non-recovered fraction (or flows, NRF, light grey). From within each zone and across the total zone, crop production can be calculated from the sum of BC, and carbon emissions can be calculated from the sum of energy use corrected for the ratio of fossil-free energy to fossil fuel.
- Water for nature is derived from the sum of water purposively allocated to nature (prior to allocations to irrigation), plus the apportioning of water from: (1) water that is not non-withdrawn into irrigation; and (2) recovered water draining from the primary and expansion irrigation zones. It is assumed that no further recovered losses emanate from the irrigation reuse zone.
- Allocation of water saved from irrigation to other sectors can only occur via a reduction in aggregate basin-level water consumption, or in other words, real water savings (Seckler 1996). Although the distinction between real and paper (dry) savings is fully recognised, this paper uses the word ‘savings’ to signify ‘real’ reductions in basin-level consumption.

### 2.2 Designing and validating a user-friendly Excel model

The primary goals of building iGains4Gains were to develop a useable functioning model of a water-food-energy nexus that reflects; purposive water allocation; changes to irrigation management in a river basin; average parameters of the basin under different scenarios; and a focus on key inputs and outcomes that users can manipulate.

We selected Excel as our model software and designed the model so that users with a modicum of irrigation and water knowledge could explore different questions and ‘what-if’ scenarios. The model iGains4Gains enables one baseline case and three future cases or scenarios to be explored. Each case employs 12 month data and time-steps, so that, for example, the annual rather than daily or monthly rainfall is entered. Scenarios, where management variables are changed, are set in the future at a moment decided by the user. Example questions and scenarios that guided model development included; what irrigation variables best explain changes in water depletion; does changing the irrigation area alter nexus outcomes; what happens if water conservation increases water consumption; and does a switch to drip irrigation increase or decrease carbon emissions? Excel also allows users and developers to unpack underlying questions, transparently examine the equations in the model, and build more advanced versions of iGains4Gains.

To build and validate our iGains4Gains model, we selected existing data and characteristics from the AZ basin in Jordan (see section 3) with the purpose of apportioning the basin supply to different sectors and dispositions following before-and-after changes in water allocation and irrigation management. In other words our case study, and its data, were not interpreted for conventional hydrological modelling of the AZ basin that mainstream hydrologists might use to study rainfall-runoff behaviours (Huang *et al* 2017).

### 2.3 The four nexus gains and implications for the maths

Achieving desirable water and societal objectives, also recognising negative externalities, means we count a nexus gain when it is a positive or normative good for people and the environment. The four nexus gains are reducing water consumed, sustaining or increasing crop production, cutting carbon emissions, and sustaining or boosting water for nature. Each nexus gain depends on the mathematical computation of the outcome in T2 compared to the baseline in T1. The metrics and graphs produced in section 3 below demonstrate these decisions. For all metrics, the manual in appendix A gives more information.

The first gain is real water savings achieved or increased, measured in cubic hectometres (hm^3^) of water across the total irrigated area. Real water savings occur from reducing total zone/aggregate irrigation depletion (synonymous with water consumption). Aggregate depletion is the sum of beneficial crop water consumption, non-beneficial consumption (NBC) and non-recovered flows (NRF) from the three zones (proprietor primary, proprietor expansion and neighbour). A nexus gain is when real water savings increase, thus when aggregate depletion in T2 is less than depletion in T1. A nexus reversal is when real water savings do not occur, meaning aggregate depletion increases (rebounds) over time.

The second gain is crop production maintained or increased, measured in total kilotonnes (kt) across the total irrigated area. A nexus gain is when the baseline T2 total zone crop production is the same as or higher than in the baseline T1 case (expressed in tonnage and as a percentage change). Since crop production is tallied to BC, this nexus gain occurs when total zone BC in T2 (=Primary BC + Expansion BC + Reuse BC) remains the same as or is higher than T1. This cross-check of the change in aggregate depletion against the change in BC is the pareto check commonly referred to in the literature (Pérez-Blanco *et al* 2020). The model focuses on crop tonnage because modellers can input the relevant crop water productivity (equivalent to the crop water use efficiency in the model) to derive total crop production. A future version of the model could apply food calories or food values, but these variables would add another degree of freedom that complicates the question of whether real water savings lower total crop production (Pérez-Blanco *et al* 2020).

Third, savings of carbon dioxide emissions are increased, as measured in kilotonnes (kt) across the total irrigated area. In other words, a nexus gain occurs when carbon dioxide emissions are reduced. This means T2 irrigation-derived carbon emissions are reduced in T2 compared to T1. Emissions come from total zone energy use in operating irrigation, factored by the percentage of energy sourced from renewables. Energy use is derived from the two main parts of operating an irrigation system: lifting water (if not sourced by gravity) and pressurising a piped network (also if not fed by gravity). The energy required for irrigation starts from two key decisions (Jensen 1983); (i) the technology selected because drip uses lower hydraulic pressures than sprinkler irrigation; and (ii), the design of irrigation scheduling which determines dosages and flow rates, which in turn affect pressure in the pipe network. The energy requirement is then converted to a carbon dioxide emissions equivalent, assuming that diesel oil is used to run irrigation pumps. These CO_2_ emissions are then corrected for the percentage of energy sourced from non-CO_2_ emitting nuclear, solar or wind power. Summarising, this metric also acts as a pareto check that conserving water by adopting new irrigation technology does not harm others; i.e. contribute to global carbon emissions.

Fourth, water for nature is sustained or increased, measured in differences in the percentages of basin water apportioned to nature. Recall, that water for nature is calculated by combining the purposive priority allocation of water to nature in Step 1 of figure 2 added to the water for nature that comes from the non-depleted irrigation water due to changes in irrigation water withdrawals and its dispositions, including how URF is divided between nature and society. The maths representing how water allocated to nature as a nexus gain examines the ‘gain in percentage points’ in the proportion of total basin water allocated to ‘water for nature’ moving from T1 to T2. Thus, if 4% of total basin water is for nature in T1 increases to 6% in T2, then a nexus gain of 2% has occurred. However, if the proportion of water for nature in T2 is 3% compared to 4% in T1, then a nexus reversal of 1% occurred. While we appreciate that environmental flows can be expressed by metrics of hydrological variability such as exceedance values (Godinho *et al* 2014), our approach to calculating water for nature as a percentage and percentage points change: (1) aims to present a straightforward metric and (2) is constrained by the time step of the model (a single year).

### 2.4 The iGains4Gains manual and 14-stage model

**Figure 4:**
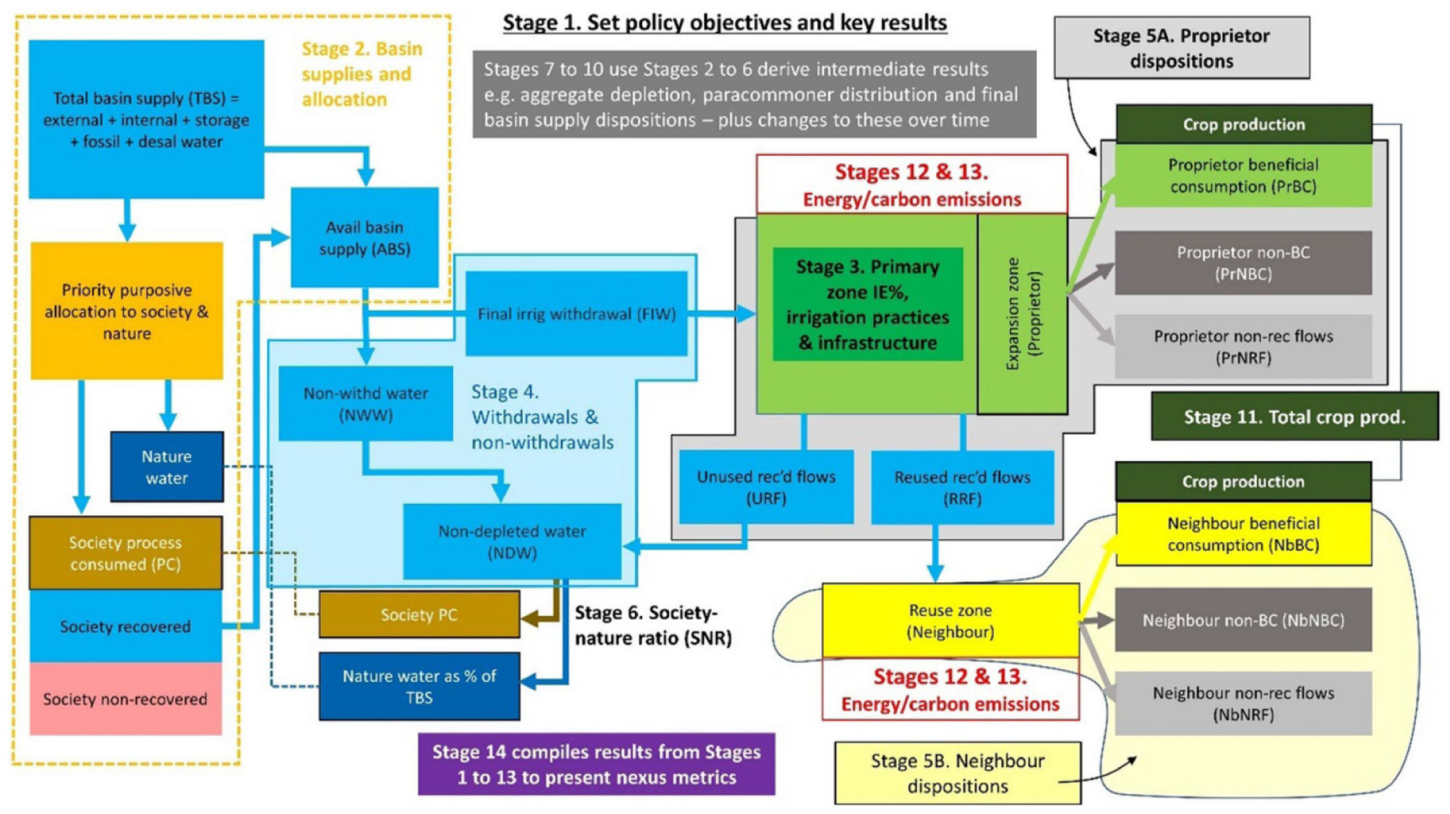
The 14 stages of the iGains4Gains model using figure 3.

The iGains4Gains Excel model breaks down the tasks in figures 1–3 into 14 stages. Figure 4 draws the 14 stages onto the block diagram of figure 3 to explain how each part sits within the model as a sequence of decisions, noting that users will usually operate the model iteratively. Described in detail in appendix A, their functions are briefly outlined here:

- Stage 1 establishes multiple scenarios of water saving and redistribution, and presents some headline nexus results. With only three future scenarios to compare against the baseline, modellers and decision-makers use Stage 1 to confirm the objective for each scenario. For example, to sustain crop production whilst effecting real water savings with a lower priority given to reducing carbon emissions.
- Stage 2 selects the river basin water supplies, priority allocations and return flows. This stage establishes both what water is available to the system and the purposive allocation of water to two main priority sectors: society (domestic, industrial, tourism) and nature. The model assumes water is first given to society and nature before allocating water to agriculture. Allocations to agriculture are determined in Stages 3 and 4. Model users can iterate between these stages to obtain allocations that meet real-world observations.
- Stage 3 sets the input variables and initial calculations for ‘irrigation needs planning’, which includes setting the irrigation efficiency hydrology. Drawing on FAO (1999), this Stage employs user-defined climate, water, land and irrigation variables to feed through to Stage 4 to establish net and gross irrigation water requirements and, from these, the water allocations to irrigated agriculture. These variables should reflect changes in irrigation technologies and management. For example, drip irrigation is regarded as being more efficient than sprinkler irrigation, which is thought to be more efficient than gravity/surface irrigation (Jensen 1983). In addition to irrigation efficiency, other examples of practices and variables include the irrigation command area, improved capture of rainfall, changes in cropping seen in different average areal crop factors, and the application of deficit irrigation.
- Stage 4 draws on Stage 3 results to compute irrigation withdrawals and non-withdrawals. Actual withdrawals depend on the rules applied to the T1 baseline and T2 scenarios. There are three rules for determining the withdrawals. 1) Applying the baseline irrigation withdrawal (BIW) keeps the withdrawal in T2 the same as in T1. 2) If the gross irrigation demand has changed, the required irrigation withdrawal (RIW) resets the T2 withdrawal to what it should be. 3) A user-defined withdrawal allows for flexibility in setting the withdrawal volume. (Note that the baseline BIW and RIW are equivalent). The remaining non-withdrawn water is then part of the NDW available for use by society and nature.
- Stages 5A and 5B use Stage 3 choices to analyse volumetric dispositions in the proprietor and neighbour, respectively. The proprietor’s five dispositions are BC, NBC, NRF, RF and URF. The model assumes the neighbour depletes all the RF from the proprietor, so the three dispositions of the neighbour are BC, NBC, and NRF.
- Stage 6 computes the total of the NDW from the sum of the non-withdrawn water and URF from the proprietor. It also sets the society-nature ratio for sharing NDW between society and nature.
- Stage 7 pulls together the results from earlier stages to compute the aggregate water depletion (AWD), aggregate depletion change (ADC) and the maximum real water savings (MRWS). The AWD is the total zone sum of the fractions: BC, NBC and NRF. The ADC is the total reduction in water depletion arising from irrigation efficiency and other management decisions. Thus, ADC is the change in AWD from T1 to T2. The MRWS is the maximum negative ADC across all three future scenarios.
- Stage 8 works out the redistribution of water between paracommoners due to water conservation in the irrigated system. The four paracommoners are the proprietor, neighbour, society and nature. This paracommoner redistribution excludes the effects of purposive water allocation between society and nature effected in Stage 2. Stage 10 (below) combines Stages 2 and 8 to determine overall outcomes.
- Stage 9 derives other useful metrics. Examples include; the final total zone irrigated area; the mm depth applied at the field level; and the contribution to crop BC from other informal supplementary water sources not treated as water withdrawals from basin supplies.
- Stage 10 uses the results of Stage 2 water allocation and Stage 8 paracommons redistribution to calculate five final water dispositions of the whole basin supply. These are total irrigation zone BC, total society process consumed, total water for nature, society non-recovered fraction and total irrigation depleted losses. Note that total zone BC can be broken down into proprietor BC and neighbour BC, a computation that gives six final dispositions.
- Stage 11 conducts pareto-checks on crop production due to changes to purposive allocations, withdrawals and irrigation hydrology. The modeller enters a crop water use efficiency value (tonnes/hm^3^) to express the total zone BC (hm^3^) in kilotonnes of crop grown.
- Stages 12A to 12D determine the changes to the irrigation energy requirements. Key inputs in Stage 12 are either drawn from earlier Stages (such as the area under irrigation) or specific Stage 12 inputs. Factors that affect energy requirements (Jensen 1983) include: the percentage and hectares under irrigation technologies of gravity, drip and sprinkler; the aquifer depth that water is lifted from; soil and crop agronomic design selections that affect system flow rates; typical operating pressures of drip and sprinkler piped networks; and the number and command areas of irrigation pumps.
- Stages 13A to 13D calculate the carbon dioxide emissions and CO_2_ savings from the energy requirements derived in Stage 12. The only input (user-defined) variable in Stage 13 is the percentage of energy sourced from non-carbon-emitting renewable sources such as solar and wind. A saving in carbon emissions is computed from the difference between the baseline (T1) and T2 carbon emissions, expecting that T2 emissions are less than the T1 emissions. However, a reduction in carbon savings can arise from farmers opting to pump more using fossil fuels.
- Stage 14 compiles key results from Stages 1–13 to present the main nexus metrics plus subsidiary information.

### 2.5 Future versions of the model

Either working in Excel or with other software, future versions might see various upgrades and changes. Examples include an online version, or adding agent-based modelling. The latter offers advanced or wider modelling, which could adopt other modules such as water and energy pricing or more details on farmer decisions. However, agent-based modelling tends to be very data-intensive and difficult to validate since it typically depends on assumptions of agent behaviour. Alongside crop yield, other additions could model food calorific and economic outcomes. Another supplement could add sensitivity analyses to identify which model parameters most effectively generate sought-for outcomes. However, conducting sensitivity analysis requires further data and time, which was not available to us at the stage of model development. In addition, reporting on sensitivity analyses would detract from our chosen focus on explaining how iGains4Gains functions to derive the four key nexus gains.

## 3 Amman-Zarqa (AZ) basin in Jordan

### 3.1 Introduction

**Figure 5:**
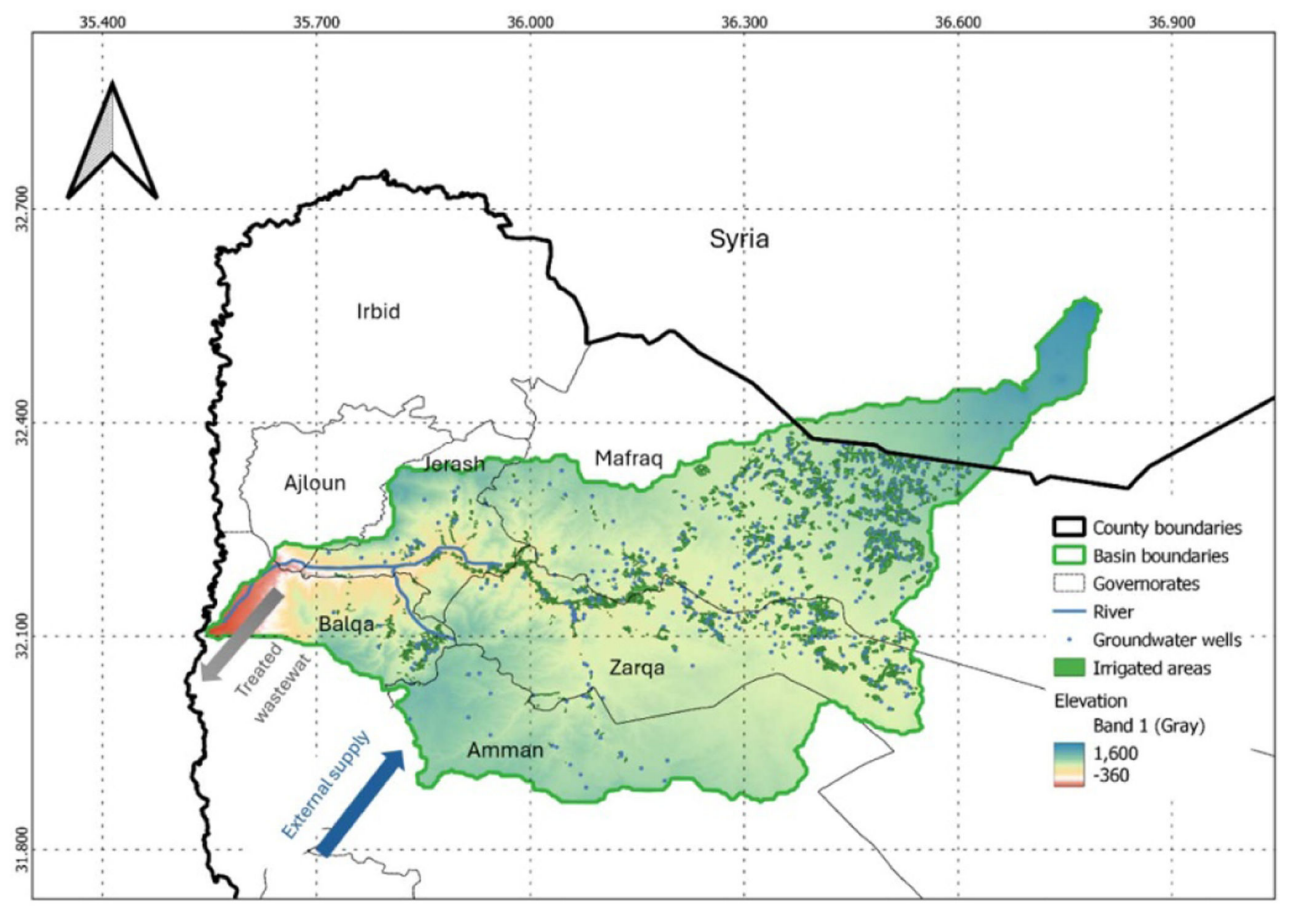
The Amman–Zarqa (AZ) river basin.

The Amman-Zarqa (AZ) Basin in the north-western part of Jordan is part of the larger Jordan River Basin (figure 5). The AZ basin covers an area of approximately 4 100 km^2^, with 93% within Jordan and the remaining 7% within Syria. The basin is naturally characterized by an arid to semi-arid climate with limited water resources (the mean annual rainfall is approximately 200 mm). It hosts over 60% of Jordan’s population, 80% of its industries, and significant agricultural activities (Al-Omari *et al* 2013). Given the high water demand from various users and limited water availability, the basin is currently experiencing the consequences of extensive groundwater over-extraction, evidenced by the rapid decline in groundwater levels, with depths in some areas approaching 500 m (MWI 2015).

We selected the AZ basin as our case study to test and develop iGains4Gains because it faces significant water allocation and irrigation management challenges, which the model is well positioned to answer. These challenges include a rapidly changing semi-arid climate plus significant population and economic growth. Combined, water scarcity and increasing competing demands intensify the complexity of water allocation decisions in the basin. The iGain4Gains model is particularly relevant as it offers a structured approach to understanding the intricate dynamics between available water supply and demand where irrigation is a major consumer of water. Thus, the model supports evaluation of the potential for water savings in irrigated agriculture in the basin and the exploration of choices for reallocating water while monitoring the hydrological impacts and broader nexus implications of reallocation decisions. Another pragmatic reason for selecting the AZ basin stems from the availability of key data covering most of the model inputs.

### 3.2 Establishing future scenarios

To reveal how iGain4Gains calculates nexus changes requires the development of future T2 scenarios, which can be compared to the T1 baseline case. Although the three future scenarios are set for this paper, other model users applying the model to the AZ case study or other basins may select their own future scenarios. The objectives for each of the three future scenarios of the AZ basin are given below. The selected scenarios in this paper explore how changes in irrigation efficiency and irrigated area impact nexus outcomes under climate change. Some variables were chosen based on available data, particularly those related to basin supply (Stage 2) and evapotranspiration changes under climate change (Stage 3), further explained in section 3.3. However, the remaining input variables were selected for their ability to demonstrate the working of the model and trade-offs in nexus gains, and to promote dialogue, rather than to present accurately forecasted and officially recognised future goals. For example, there is no official target for delivering water for nature, which also recognises how politically unpalatable this is in this populous, arid and over-extended basin. Similarly, there are no goals or policies addressing specific changes in irrigated areas required under future climate change. However, in all three future scenarios, irrigation efficiency increases.

The aim of Scenario 2 is to show an increase in both water consumption by irrigation and total crop production, higher carbon emissions and a minor gain in water for nature. The rebounds in water consumption and carbon emissions are driven by increases in irrigation efficiency but with no complementary reduction in T2 irrigation withdrawals or additional use of renewables.

Scenario 3 applies a higher efficiency, cuts the irrigated area and imposes a required water withdrawal (not the baseline withdrawal (BIW)) to derive real water savings for nature. The cut in irrigation sees crop production drop by nearly a third, but this cut, plus greater use of renewable energy, delivers considerable carbon savings. Scenario 3 sees the highest amount of water provided to nature.

Scenario 4 uses a higher irrigation efficiency, applies the RIW as the withdrawal rule, and sets a goal-seek solution for its starting irrigated area to create no change in water consumption (real water savings = 0.0). Compared to the baseline, this scenario delivers gains in crop production, considerable savings in carbon emissions, and slightly more total basin water to nature.

### 3.3 Setting out the supply-side and purposive water allocations

Table 2 summarizes the data used to quantify the AZ basin supply and priority allocation under the baseline scenario (T1), and three future scenarios under climate change (Sc 2, Sc 3, Sc 4). Data utilized to develop the T1 scenario were taken from the Ministry of Water and Irrigation (MWI) for the hydrological year 2020 (September 2019 to August 2020) and are described below:

- ***Supply sources*** include; (1) internal renewable groundwater supply estimated at 87 hm^3^ yr^*−*1^, which aligns with the safe yield of the basin’s aquifers (MWI 2015); (2) surface water from the Zarqa River of 25 hm^3^ yr^*−*1^ (Amdar *et al* 2024); (3) to meet the high demand, the basin further receives an external supply totalling 165 hm^3^ yr^*−*1^, primarily piped water from the Disi fossil aquifer located in Southern Jordan to support municipal supply (Amdar *et al* 2024); and (4) an additional 90 hm^3^ yr^*−*1^ of water sourced from internal fossil water reserves within the basin^7^. Collectively, these resources provide 367 hm^3^ yr^*−*1^ of water for the basin.
- ***Municipal water supply:*** the estimated water supply is 87 l/c/day (MWI 2023). However, due to the significant losses in municipal networks, which amount to 47% (MWI 2023), the actual net water supply could be as low as 46 l/c/day.
- ***Priority allocation in the basin*** encompasses municipal, industrial, and tourism sectors. Municipal supply, estimated based on a 87 l/c/day demand, totals 174 hm^3^ yr^*−*1^. Industrial water allocation is 40 hm^3^ yr^*−*1^, and tourism allocation is less than 1 hm^3^ yr^*−*1^. This brings the total priority non-agricultural water allocation to 215 hm^3^ yr^*−*1^.
- ***Priority water for nature allocation*** is set at 0.0 hm^3^ yr^*−*1^ under all scenarios. However, water is provided to nature via return flows and savings from irrigation.
- ***Water consumption from priority allocation*** is estimated at 20%, 80% and 80% of water allocated to the domestic, tourism and industrial sectors, respectively, equivalent to a collective net use of 67 hm^3^ yr^*−*1^.
- ***Return flows from priority allocation***; the remaining water allocated to priority sectors but not consumed is 148 hm^3^ yr^*−*1^, equivalent to the volume of treated wastewater (TWW) generated within the basin. Of this amount, 10% is recovered for restricted irrigation along the Zarqa River (Al-Bakri *et al* 2016), while the remaining return flow, referred to in our model as the ‘society non-recovered fraction’, is discharged into the Zarqa River and transferred to the Jordan Valley for irrigation (which is not included in the model).

Concerning future scenarios Sc 2, Sc 3, and Sc 4 variables in the model, projections on the basin’s water resources under climate change by 2050 were sourced from Jordan’s 4th National Communication Report (UNDP and Ministry of Environment, 2022). The report provides detailed projections of future water supplies in the AZ basin described in the following points:

- ***Supply sources***: the basin is projected to experience an average reduction in recharge by 2050 compared to the baseline period of 1990–2020. This reduction could decrease the internal renewable groundwater supply to 73 hm^3^ yr^*−*1^ and fossil water supply to 50 hm^3^ yr^*−*1^ by 2050. Similarly, surface water supply is expected to decline to 21 hm^3^ yr^*−*1^. Given the anticipated downward trend in water availability in Jordan, supply from external sources is also projected to decrease by 20% to 134 hm^3^ yr^*−*1^. To cover the future water demand gap, the government has initiated the National Desalination Project. This project will provide around 300 hm^3^ yr^*−*1^ of desalinated seawater by 2040 (MWI 2023). Our model assumes approximately 120 hm^3^ yr^*−*1^, given that not all of this will be achieved or delivered entirely to the AZ basin. Collectively, these resources could raise the basin supply to 398 hm^3^ yr^*−*1^ by 2050.
- ***Municipal water supply***: with an anticipated future gross demand of 100 l/c/day, and accounting for water losses in municipal networks of 25%, the anticipated net per capita supply in the municipal sector is projected to be 75 l/c/day.
- ***Priority allocation*** in the basin is expected to increase to 356 hm^3^ yr^*−*1^ due to population growth reaching approximately 8.5 million. Allocation for industries is projected to rise to 45 hm^3^ yr^*−*1^, while the tourism sector’s allocation will remain slightly less than 1.0 hm^3^ yr^*−*1^.
- ***Process water consumption from priority allocation*** was estimated at 20%, 80% and 80% of water supply to the domestic, tourism and industrial sectors, respectively.
- ***Return flows from priority allocation*** were estimated at 257 hm^3^ yr^*−*1^. Of this, 142 hm^3^ yr^*−*1^ can be recovered within the basin and used for expanding irrigation with TWW along the Zarqa River. The remaining flows via the Zarqa River out of the basin to the Jordan Valley, where it is either lost or used by irrigation.

Based on the data explained above, the remaining available basin supply after allocation to priority users was estimated at 167 hm^3^ yr^*−*1^ under the baseline T1 scenario, and increases to 184 hm^3^ yr^*−*1^ in the three future scenarios.

### 3.4 Establishing the irrigation requirements, efficiency hydrology and withdrawals

In the next step (table 3), we estimated the irrigation water requirement and final irrigation withdrawal (FIW) for the baseline T1 period using the data below:

- ***Irrigated area***, which represents the starting proprietor area within the AZ basin, was estimated at approximately 17 000 ha (Shammout *et al* 2021). These areas are clustered in the north-eastern part of the basin within the Mafraq governorate. Nearly 85% of this irrigated land is cultivated with orchards such as stone fruits and olives, while the remaining area is used for seasonal vegetables. Most irrigated areas utilise drip irrigation, while fewer farms still use high-flow mini-sprinklers primarily on stone fruits (Al-Raggad and Fraj 2019).
- ***The reference crop evapotranspiration and rainfall*** over irrigated areas in the basin were derived from the FAO’s Water Productivity portal through Open-Access to level 2 remotely sensed data. The average crop evapotranspiration was 1736 mm yr^*−*1^, while the average rainfall was 202 mm yr^*−*1^ in 2020.
- ***The actual crop evapotranspiration***: given the dominance of fruit orchards, their irrigation season commencing from February to November, the average areal crop factor was estimated at 0.5. Considering a field reduction factor of 0.95, and a deficit irrigation factor of 0.9, the average actual crop evapotranspiration in this region was calculated at 742 mm yr^*−*1^. This value was validated against that estimated for this region between 2017 and 2019 at between 716–722 mm yr^*−*1^ (Al-Bakri *et al* 2023).
- ***The net irrigation requirements*** were thus calculated at 581 mm yr^*−*1^, assuming a contribution of 162 mm yr^*−*1^ of effective rainfall, where 742–162 = 581.
- Considering the weighted average efficiency per irrigation system type and conveyance efficiency, ***the field application depth*** was estimated at 759 mm yr^*−*1^ under the baseline scenario.
- The above inputs resulted in a gross irrigation requirement of 841 mm, and a FIW (termed the BIW in T1) of 143.0 hm^3^.

Also, in table 3, and following the same approach, the field application depths and water withdrawals were estimated under the T2 scenarios (Sc 2, Sc 3, and Sc4) as follows:

- ***Starting irrigated areas*** were maintained at 17 000 ha under Sc 2, equal to the baseline scenario T1. However, irrigated areas were decreased to 14 000 ha under Sc 3 and increased to 18 930 ha under Sc 4 (derived using Excel’s goal-seek to achieve no change in depleted water). Note that the final irrigated areas differ from the proprietor’s starting area due to irrigation expansion or reuse.
- ***Average crop evapotranspiration*** is projected to increase by an average of 4% by 2050, from 1736 mm yr^*−*1^ to 1813 mm yr^*−*1^. However, rainfall is projected to decrease from 202 mm yr^*−*1^ in 2020 to an average of approximately 95 mm yr^*−*1^ by 2050 (UNDP and Ministry of Environment 2022). Accordingly, the net irrigation requirements increase from 581 mm yr^*−*1^ in the baseline case to 658 mm yr^*−*1^ for all three future scenarios.
- ***Improvements in farm irrigation efficiency*** were assumed, taking sprinkler irrigation up to 70% efficiency and drip irrigation to 85% efficiency in Scenarios 2 and 3. This resulted in a new lower average field application depth of 788 mm yr^*−*1^. In Sc 4, drip was applied to 100% of the AZ basin, accompanied by an increased efficiency to 90%, delivering a lower field application depth of 688 mm. These increases in efficiency mean that, even though net irrigation requirements increase due to climate change, gross irrigation requirements decrease from 841 mm yr^*−*1^ in Case 1 to 788 mm yr^*−*1^ for Scenarios 2 and 3, and down to 731 mm yr^*−*1^ in Scenario 4.
- ***The FIW*** was set using the BIW for Scenario 2 and the RIW for Scenarios 3 and 4. This resulted in with-drawals for Scenarios 2, 3 and 4 of 143.0 hm^3^, 110.4 hm^3^ and 138.5 hm^3^ respectively. These withdrawal rules were deliberately chosen to demonstrate different nexus trade-offs.

### 3.5 Establishing changes in energy use

Table 4 presents the key input selections that drive the changes in energy use, both in terms of energy requirements for irrigation and the proportion of energy sourced from non-GHG renewable sources. Regarding the latter, this is set at 30% in the T1 case (NEPCO 2022). For Scenario 2 it is maintained at 30%, but is increased in Scenarios 3 and 4 to 65% and 80%, respectively. The lift height from the deep aquifers is set at 300 m for the baseline, increasing to 330 m in all three future scenarios (MWI and BGR 2019). It is important to note that there is no gravity-fed field irrigation in all four scenarios, and that all changes occur within sprinkler and drip technologies.

### 3.6 Deliberative dialogue using iGains4Gains at a workshop

An early version of iGains4Gains was tested at the Nexus Advanced School for early career researchers at Hashemite University, Zarqa, Jordan, in February 2024. Twenty course participants from the MENA and Sub-Saharan Africa regions participated in five days of lectures, course work and field trips. The model testing aimed to determine how readily course participants could understand and apply it.

## 4 Results

### 4.1 Nexus outcomes

**Figure 6:**
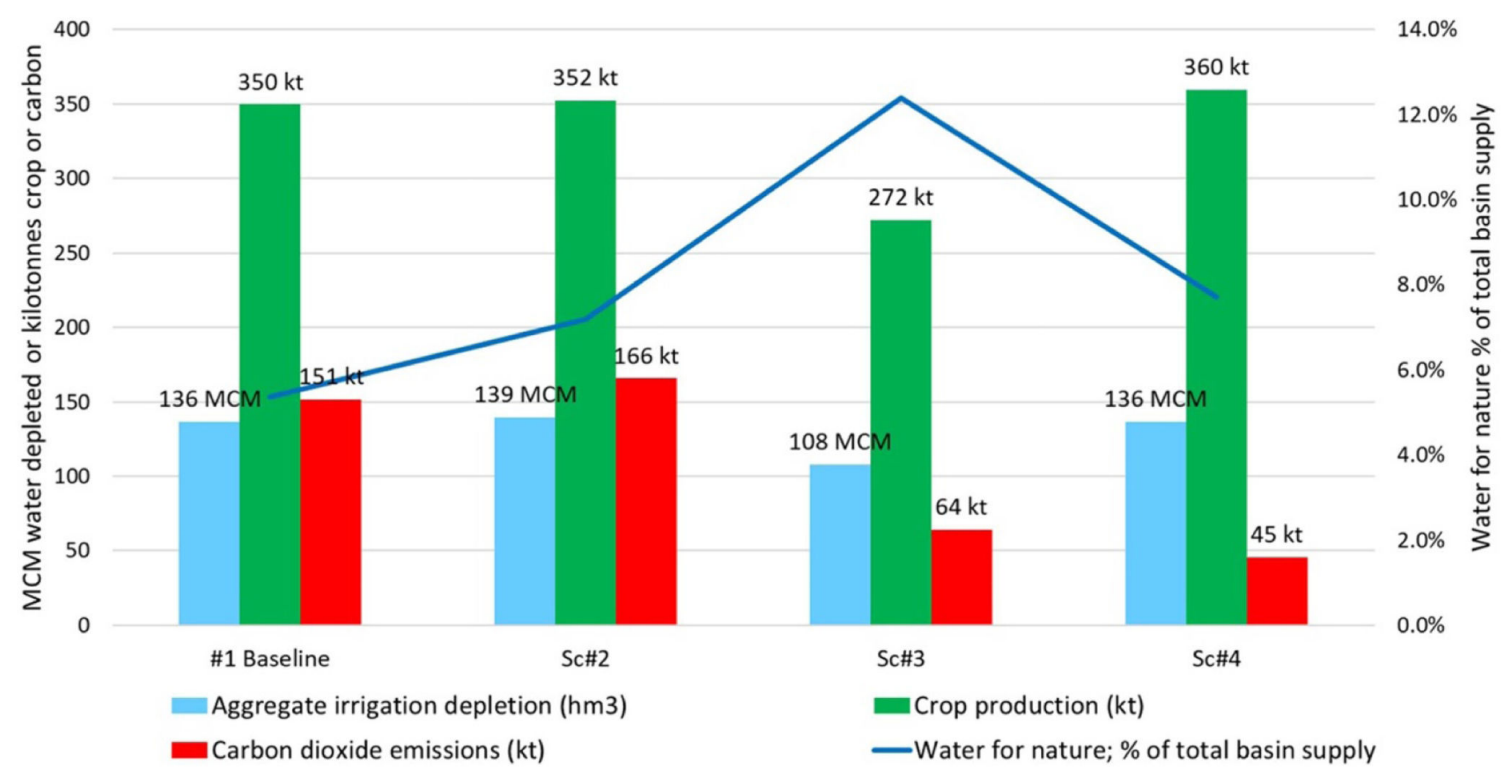
Total water depletion, crop production, carbon and water for nature for four cases.

**Figure 7:**
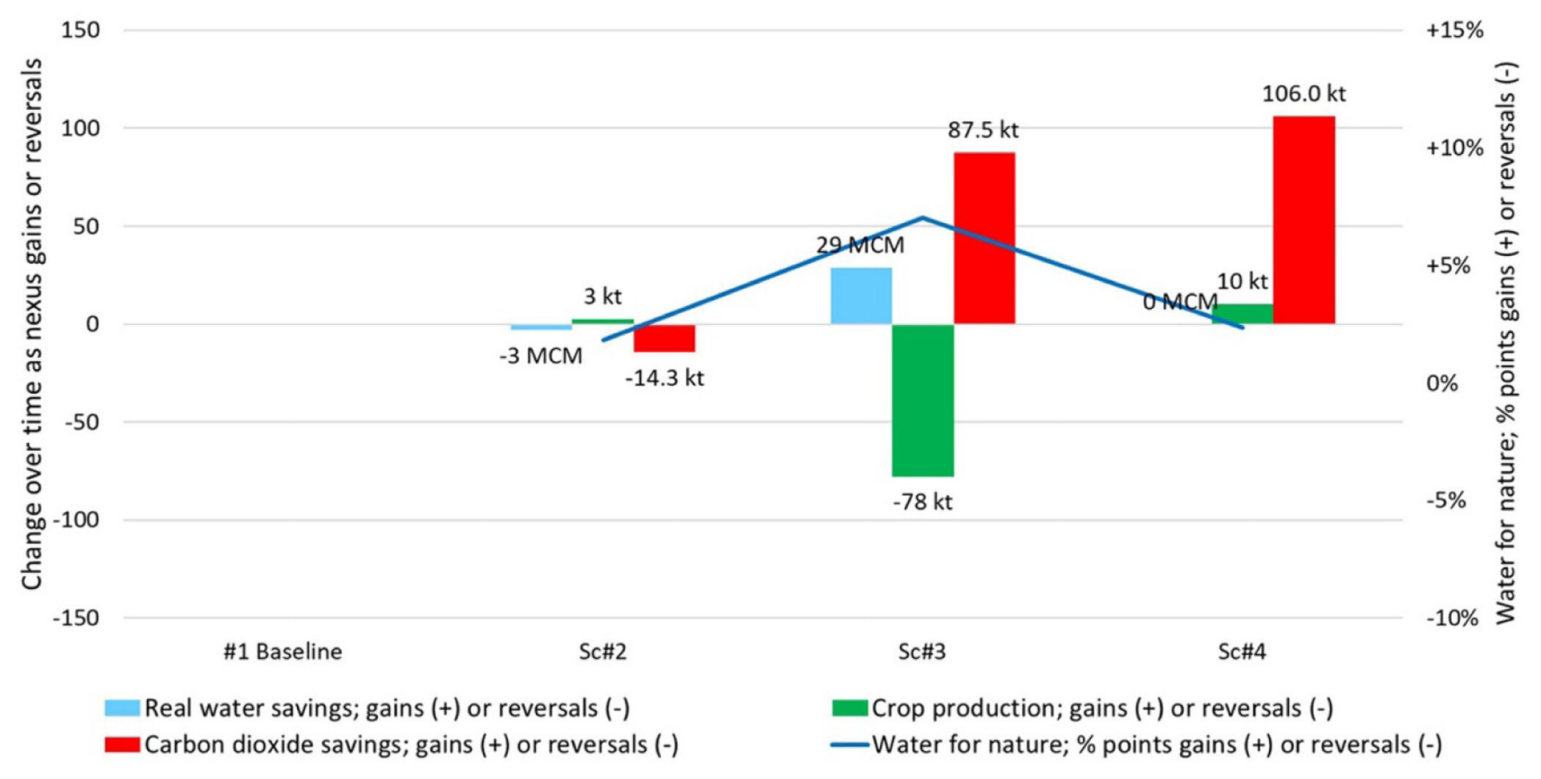
Changes in water depletion, crop production, carbon and water for nature for T2 scenarios.

**Figure 8:**
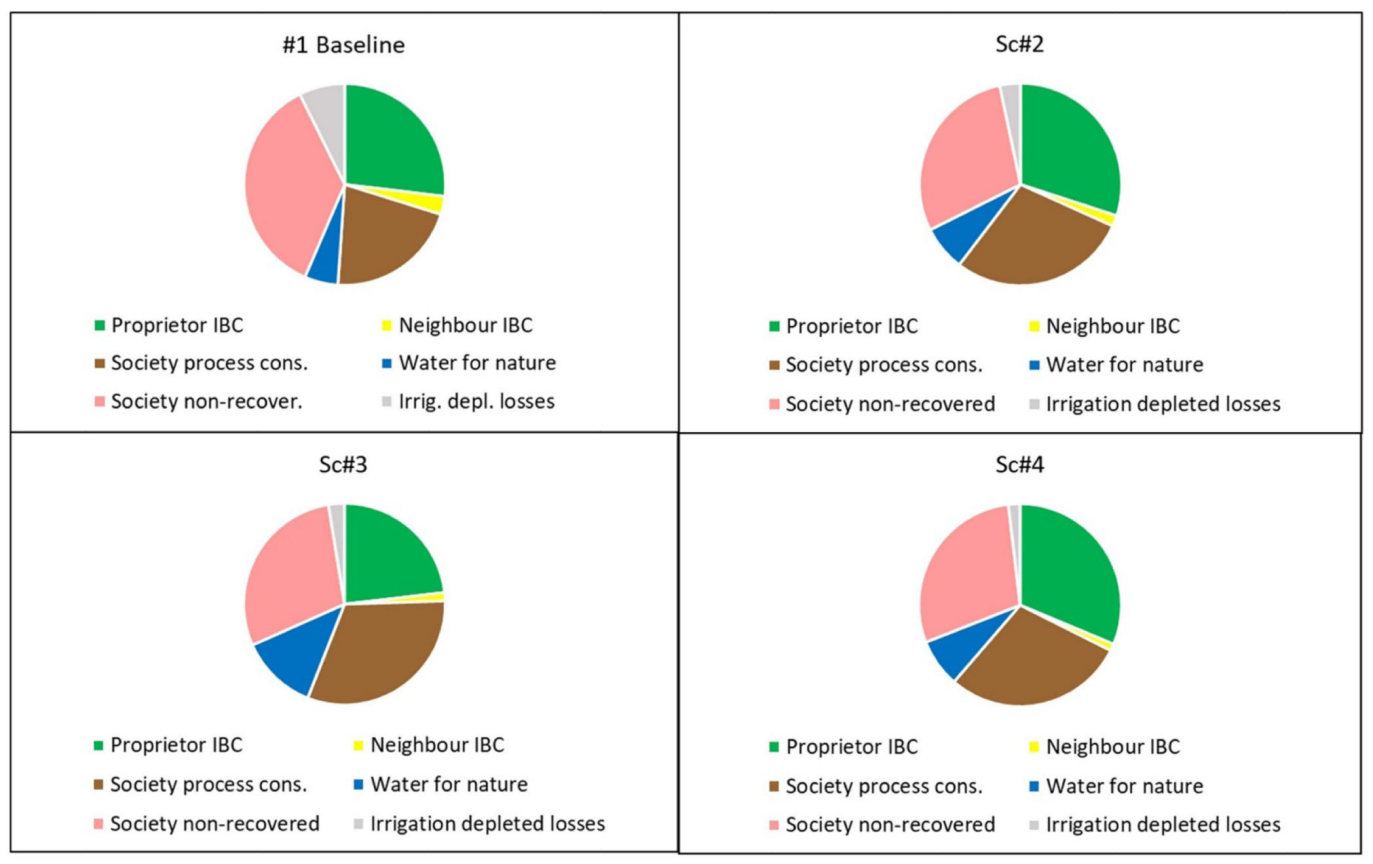
Six water dispositions of total basin supply from priority water allocation and water conservation for the four scenarios.

**Figure 9:**
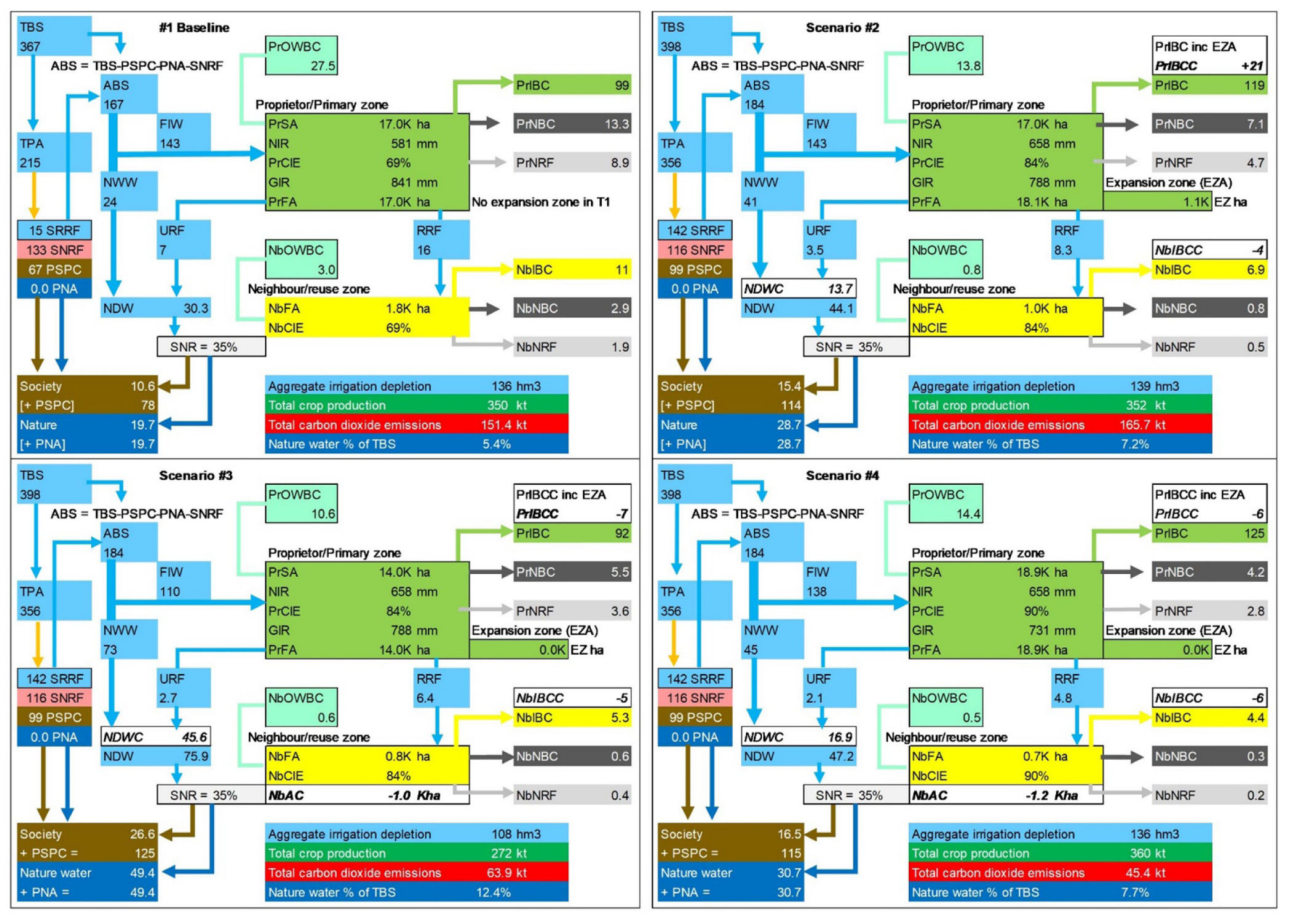
Water distribution as block diagrams from water allocation and conservation across four scenarios.

Table 5 and figures 6–9 present key results from the analysis of the AZ basin, the baseline case, and three future T2 scenarios. Note figure 6 presents the results for water depletion, crop production, carbon and water for nature for all four cases, while figure 7 presents these outcomes as nexus gains or reversals for the T2 scenarios. Regarding nexus outcomes, the following observations can be made:

In Sc2, real water savings do not occur because aggregate depletion increases by about 3 hm^3^. Note: paper savings are achieved when defined by field applications (which decrease by about 33 mm). However, defined by changes in withdrawals, there are no paper savings, as Scenario 2 withdrawals remain the same as the baseline case. For crop production, there is a nexus gain because both BC and crop production increase. Crop production increases in Scenario 2 by 2.7 kilotonnes. Carbon dioxide emissions increase by 9% for this scenario, causing a nexus reversal for carbon. Water for nature increases from 5.4% in the baseline to approximately 7.2% in Scenario 2, representing a nexus gain of about two percentage points.

For Sc3, there is a clear nexus gain in both carbon emissions and water savings but at the cost of crop production which goes down by *−*22%. The latter decreases due to less crop-BC and a smaller irrigated area (the total final area drops to less than 15 000 ha). This scenario delivers the greatest amount of water for nature, up to approximately 12% of the total basin supply.

Sc4 sees four nexus gains against baseline delivered for crop production (up by 10 kt), neutral water savings (via no change in water depleted), carbon emissions savings of 106 kt, and water for nature up by 2.3 percentage points.

### 4.2 Water redistribution from water allocation and irrigation water conservation

The iGain4Gains model determines the changes in the final six dispositions of water flowing due to both priority water allocation decisions taken in the river basin, and agro-hydrological water savings promulgated within the irrigated system. The six dispositions; are proprietor irrigation BC, neighbour BC, society process consumption, water for nature, society non-recovered fraction, and irrigation depleted losses. These changes can be seen in table 6 and figures 8 and 9. With respect to the three future scenarios; the three main dispositions in the AZ Basin are the proprietor’s irrigation BC, society’s process consumption and the SNRF from society.

Expressing the final water dispositions; the proprietor gets most water (30% and 31%) in Scenarios 2 and 4, respectively. However the proprietor’s water share goes down from 27% in the baseline case to 23% in Scenario 3. The neighbour (the reuse zone) sees a reduction in supply for all three future scenarios as the proprietor’s irrigation efficiency increases and when less water is withdrawn for irrigation in Scenarios 3 and 4. Society process consumption increases from approximately 78 hm^3^ to above 100 hm^3^ in all future scenarios, largely reflecting the increase in population. Water for nature increases in all future scenarios, mainly via a boost from desalinated water (up from 0 hm^3^ to 120 hm^3^). Although desalinated water is for municipal priorities, this indirectly relieves scarcity pressures for all users. The share of water for nature is the greatest in Scenario 3 at 12% of the total basin supply.

## 5 Discussion

### 5.1 Optimising nexus outcomes and recognising trade-offs

The iGains4Gains model reveals it is difficult to optimise all four nexus gains. Instead, the model reveals that trade-offs between the four nexus indicators are much more likely, a conclusion also arrived at by Payet-Burin *et al* (2019). Simultaneously boosting all four individual nexus outcomes (real water savings, crop production, reduced GHGs and improved water for nature) is achievable in the iGains4Gains model as demonstrated by Scenario 4. Nonetheless, this requires careful control over several key input variables best informed by a dialogue between interested and affected parties. All four nexus indicators remain the same or go up when three main factors are controlled: (1) withdrawals and irrigated area are together constrained to prevent rebounds from irrigation efficiency improvements (Lankford 2023); (2) depleted irrigation losses are reduced and are commuted to BC (crop production) and water for nature; (3) increased efficiency is achieved without using additional energy to run pressurised and pumped systems, or if this is the case, renewable energy is utilised so that GHG emissions are eliminated.

However, these three controlling factors may not apply in the real world. For example, suppose irrigators have the legal right to continue withdrawing their historic licences despite having made efficiency gains by adopting drip irrigation (Huffaker and Whittlesey 2000). In that case, they can increase their irrigated area with the newly freed-up water. They have, in effect, used paper savings (decreased their field application depths) to either peg their new total depletion to the baseline’s depletion or to increase depletion.

Achieving all four nexus gains is *de facto* difficult in the AZ Basin because of the exceptional pressure on limited water resources to satisfy a growing population. Furthermore, continuing groundwater abstractions, evidenced by declining groundwater levels (MWI and BGR 2019) also does not appear to be sustainable in the long term. Since the model is not built around the recursive hydrological connections between rainfall, surface water, shallow groundwater, deeper fossil water, withdrawals and consumption, the outcomes of new management changes on the levels of fossil water cannot be predicted. That said, a complete cessation of the use of fossil water is highly unlikely particularly for irrigation which comes from farmer-owned wells. A potential avenue for future use of the model is to explore the substitution of fossil water with non-conventional water sources (e.g. brackish water, wastewater, desalination). If more accurate data on fossil water availability under climate change and human water use becomes available, it can be incorporated into the model at Stage 2. One of the future scenarios could then focus on the impact of substituting fossil water with non-conventional water for irrigation. This approach aligns with Jordan’s Water Strategy 2023–2040, which includes a goal to substitute 41% of groundwater use with non-conventional water for irrigation by 2040, including areas in the highlands of the basin.

For the above reasons, the limited water for nature is the key indicator of how tightly closed this basin is. Although water for nature increases in the three future scenarios, it remains around or less than 10% of the total basin supply. In reality, it may be less than this in the future given the projected high demand for water and increased climate variability.

A further real-world difficulty arises in delivering significant reductions in GHGs. Irrigation-related GHG emissions decrease significantly in future scenarios 3 and 4. While on paper, this is to be welcomed, this nexus gain depends on a considerable step-up in electricity generation from renewables. Yet this additional supply must also meet the projected growth in energy for desalinisation and industrial and domestic use driven by a growing population and increasing affluence.

### 5.2 Deliberative dialogue on future scenarios

The rapid learning and uptake of the iGains4Gains model by twenty participants at the Nexus Advanced School at Hashemite University, Zarqa, Jordan, in February 2024 demonstrated that participants could readily understand and apply it. The process of preparing the model for the February workshop also refined the iGains4Gains model.

Interpreting the experience and feedback from this workshop, we surmise that the iGains4Gains model can facilitate and enhance locally led stakeholder co-creation of scenarios, build a shared understanding, and create a sense of ownership over basin problems and trajectories. In this regard, the iGain4Gains model is best used within a deliberative process (Payet-Burin *et al* 2019) that convenes interested parties with a stake in the four nexus gains and the factors that control them. This opportunity allows stakeholders to formulate problem statements regarding future options and to co-develop pathways to reach preferred outcomes.

Although we kept the variables between the three future scenarios quite similar, the model can accommodate many future concerns, allowing their impacts to be compared. The kinds of future concerns include: (1) climate change, seen via increases in evapotranspiration and decreases in rainfall, surface water and groundwater; (2) unregulated growth in irrigation, selected by higher proprietor starting areas or the deployment of the original BIW volumes. 3) Capture of real water savings by society rather than these dividends going to nature. This can be shown, for example, by selecting a higher society-nature ratio or higher process consumption within the domestic, industrial and tourisms sectors that make up society.

With respect to the second point in the previous paragraph, the need to regulate irrigated area expansion within the basin is a thorny challenge facing policy makers. Our model calculates potential expansion in irrigated area using a two-step process. First, it considers the proprietor’s irrigated area where efficiency measures are applied (and other means to reduce the net water requirements such as boosting rainfall capture and reduced beneficial uses to control salinity (Karimzadeh *et al* 2024). This is where losses forestalled by the proprietor (equivalent to the primary zone) are employed to expand their own area (the expansion zone) assuming withdrawals stay the same as before. Second, via the selection of an appropriate reused recovered fraction (RRF) ratio, the model accommodates water recovery to the neighbour in the reuse zone, and assumes efficiency measures are also implemented in the neighbouring reuse zone. These combine to allow for the total area to be greater than if were only dictated by the classical irrigation efficiency of, and water withdrawn into, the primary zone.

Under climate change, net irrigation requirements are projected to increase from 661 mm yr^*−*1^ in the baseline scenario to 696 mm yr^*−*1^ in future scenarios. However, implementing efficiency measures can reduce gross irrigation requirements compared to the baseline and offset these increased net irrigation requirements. On paper, this means farmers will be better positioned to face the impacts of climate change. By redistributing paper water savings from the proprietor area to both expanded and neighbouring zones, our model suggests that it is possible to maintain (SC 2) or even reduce (SC 4) irrigation withdrawals despite expanded irrigation areas.

However, given the historical context of irrigation practices in the AZ basin and lax regulations, actual growth in irrigated area, withdrawals and depletion may be unpredictable. Efficiency measures must be paired with strict limits on further irrigation expansion to avoid increasing irrigation withdrawals and depletion. Our model can help identify appropriate caps for irrigation expansion, evaluate options for constraining or reducing irrigated areas, and assess the implications for basin supply and nexus outcomes. We consider that these outcomes are predicated upon three main water and irrigation governance factors:

First, governing the implementation and consequences of further irrigation efficiency measures. Most farmers in this region rely on self-judgment to determine irrigation requirements and technologies, focusing on maximizing profits through increased crop weight, especially for fruit crops (Kafle and Balasubramanya 2023). Guiding farmers towards higher irrigation efficiency would depend on the availability, accessibility, and affordability of irrigation technologies and advisory services, and farmers’ willingness to adopt these measures. However, there is also the need to regulate how farmers turn their irrigation efficiency gains into their own (proprietary) material gain allowing them to expand their irrigated area or to apply greater depths of water within-season. This regulation may partially depend on solutions sitting within solar energy management, such as the monitoring of solar power usage and metering of water from solar pumps (Balasubramanya *et al* 2024).

Second, as well as solar-power solutions, measures coming from within irrigation will also be needed to regulate irrigation demand. For example, controls on irrigated area expansion may help. Between 1989 and 2017, agricultural areas in the basin expanded by 10%, partly due to unauthorized groundwater pumping and irrigation efficiency gains, which has exacerbated aquifer depletion (Al-Bakri *et al* 2016, Shammout *et al* 2021). Another study suggests that without limits on land expansion, increased efficiency measures could increase irrigated areas by up to 68% (Amdar *et al* 2025). Regulating the total area under irrigation is not easy, but it could be enabled by satellite image analysis paired with smartphone apps that inform farmers about their own area under irrigation, their neighbour’s areas and cumulative areas.

Third, returning to the question of how energy plays a role in governing irrigation, deliberative dialogue of changes in the costs and amounts of diesel and renewable energy illuminate future options. Farmers view the high energy costs of using diesel to pump water from deep aquifers as the primary constraint to irrigation abstractions rather than water scarcity. Thus, the benefits of a higher irrigation efficiency include reduced abstracted volumes of water (assuming no rebound) and lower diesel energy costs. The latter in particular could act as an incentive to adopt efficiency measures. However increasing adoption of solar power, which is much less costly to farmers than diesel would not incentivise irrigation performance enhancements, and instead potentially lead to greater water withdrawals and consumption (Balasubramanya *et al* 2024). Clearly dialogue is needed between farmers and irrigation services to help farmers conserve and schedule irrigation carefully to reduce irrigation withdrawals, thereby lowering irrigation costs and GHG emissions, whilst sustaining crop production, and profitability (Amdar *et al* 2025). Furthermore, even though greater use of low-cost solar power may remove energy-cost constraints (and GHG externalities), both farmers and the government need to address the declining availability of fossil water, especially if solar is employed without implementing appropriate caps on groundwater withdrawals.

## 6 Conclusions

An integrated nexus objective of reducing water consumption and carbon emissions while increasing or sustaining crop production and water for nature requires both purposive water allocation and water conservation in irrigation to be carefully governed. Our iGains4Gains model reveals that while achieving these integrated goals simultaneously is difficult, they are possible if irrigation efficiency, irrigation area, withdrawal volumes, energy usage, and other irrigation practices are carefully controlled. In addition, iGains4Gains reveals how the equity of water distribution between water-use sectors and four paracommoners, especially water for nature, may be improved on the back of irrigation efficiency gains and purposive priority water allocation.

The model is best used within a participatory deliberative forum which allows all stakeholders to co-develop objectives and build a shared understanding of the context while appreciating that nexus gains usually involve trade-offs and compromises. The model can be applied to different contexts where irrigation withdraws and consumes large volumes of water. Based on the iGains4Gains application to the AZ Basin, examples include arid and semi-arid river basins in the Middle East, or in Southern and Eastern Africa. However, we believe its application to very large humid basins in Asia would require further work to accommodate water being drawn simultaneously from shallow and deep aquifers and to cope with overlapping irrigation seasons enabled by year-round growing conditions.

## Supplementary Material

Supplementary Data

## Figures and Tables

**Table 1:** Key terms and definitions used in the main paper (see appendix A for full list).

Terms	Definition
Available basin supply (ABS)	The calculated volume of water that is allocatable to irrigation and between paracommoners after a correction of the total basin supply (TBS) minus the priority allocation
Aggregate water depletion (AWD)	The AWD is the total depletion from the total irrigation zone. It is the sum of depleted fractions across the proprietor and neighbour. AWD = PrBC + PrNBC + PrNRF + RRF (It is assumed RRF is depleted by the neighbour). Recall the proprietor in T2 can include the expansion zone
Beneficial consumption (BC), irrigation beneficial consumption (IBC) and other water beneficial consumption (OWBC)	This is the fraction of water beneficially consumed in crop evapotranspiration (ETc) for crop growth. This BC can be supplied from irrigation withdrawals (IBC) or other water such as wastewater or rainfall (OWBC). See non-beneficial consumption
Priority allocation	This is the volume of water that is always made available to nature, and society (= domestic use, tourism and industry) and is the first priority allocation made before irrigation calculations of withdrawals, depletions and distributions
Crop water productivity (CWP)	Crop water productivity (=WUE) is the useful or economic crop yield per volume of water beneficially consumed in ET
Final irrigation withdrawal (FIW)	The final irrigation withdrawal is important as it drives the agro-hydrology of the total zone and determines the non-withdrawal of water. It is the provisional water withdrawal (PWW) corrected to be equal to or less than the available basin supply because the FIW cannot exceed the ABS
Fossil fuels and fossil groundwater	Fossil fuels are carbon-based; examples include coal, petrol and diesel. Fossil water is the term given to groundwater that is not being annually recharged
Gravity irrigation, drip irrigation and sprinkler irrigation (GI, DI and SI)	The model can calculate the impacts of changes in irrigation technology on irrigation efficiency, water consumption, energy use, and carbon emissions. Salient input variables include the percentage of command area under each technology and their irrigation efficiencies
Internal basin supply (IBS) and external basin supply (EBS)	The IBS is the water volume from within the catchment. The IBS comes from both groundwater and surface water. The EBS is sourced from outside the basin
Informal supplementary water (ISW)	This is water not withdrawn from the basin supplies, but it supports crop growth. ISW comes from on-farm rainfall harvesting, small pond storage and wastewater reuse
Irrigation withdrawals (see FIW)	Irrigation withdrawals into an irrigation system divide into four water fractions. These are; the beneficial consumption (BC) of water in crop evapotranspiration; non-beneficial consumption (NBC as evaporation); recoverable flows or fraction (RF); and non-recoverable flows (NRF)
Water for nature (WfN)	Water for nature is one of the nexus gains in the model. WfN can both be purposively allocated water from total basin supplies and is also one of the paracommoners obtaining water from the non-depleted water (NDW) adjusted by effecting agro-hydrological changes in the proprietor and the society-to-nature ratio (SNR). Water for nature covers not just environmental flows but water that provides ecological benefits, such as small ponds and shallow water tables
Neighbour (Nb)	One of the paracommoners; a receiver of water discharging as drainage from the proprietor (receiving the RRF). The neighbour comprises the reuse zone if this is present
Non-beneficial consumption (NBC)	A water accounting fraction defining the amount of water depleted by evaporation that produces little or no crop production e.g. from weeds or open water
Non-depleted water (NDW)	NDW is the water that is the sum of the water not withdrawn into irrigation (and therefore is not depleted) and the water not depleted by the total zone of irrigation if it is withdrawn into irrigation
Non-recovered fraction (NRF)	A water accounting fraction that defines the amount of water not recovered to other users in the basin
Non-withdrawn water (NWW)	Both a percentage and volume, this is the amount of basin water available to irrigation but not withdrawn after the final withdrawn water flows into the proprietor is calculated
Other water beneficial consumption	OWBC is the beneficial consumption of irrigated crops using water from effective rainfall and informal supplementary water (ISW is not part of irrig withdrawals). OWBC is calculated in the model from ‘Proprietor other water beneficial consumption’, ‘Neighbour other water beneficial consumption’ which then gives the ‘total zone other water beneficial consumption’
Paracommons & paracommoners	A paracommons is a united system of water users, connected by agro-hydrological change in the proprietor irrigation system (main withdrawer and first water user). Water (re)distribution occurs between four paracommoners; proprietor, neighbour(s), nature, and society. Multiple scenarios selected to assist discussions about desirable yet unpredictable outcomes
Pareto (or pareto test)	This is a test that an effect (usually positive) in one outcome or interest does not harm another outcome or interest. E.g. water can be saved without cutting food production
Proprietor (Pr)	One of the paracommoners; the first-use withdrawer of water. The proprietor comprises the primary zone and the expansion zone if the latter is present in T2
Real water saving (RWS)	RWS is equivalent to the aggregate depletion change across the total irrigation zone when depletion in T2 is less than T1
Rebound	A rebound occurs when the T2 aggregate water depletion is higher than the T1 baseline aggregate water depletion
Recovered fraction (RF)	RF is the fraction of water that is recovered and is either consumed by the neighbour (RRF) or is unused and so flows to nature and society (URF). This split of the RF is defined by user-set inputs for RRF% and URF%
Renewable energy share (RES)	Renewable energy share corrects the potential carbon emissions from energy use in irrigation because RES determines how much of the energy comes from non-carbon energy. RES applies to the total zone and all irrigation types
Reused recovered flow/fraction (RRF)	Part of the recovered fraction as a per cent and as a volume consumed by the reuse zone (neighbour). Recall all RRF is depleted as it is assumed no further recovered flows issue from the neighbour
Society nature ratio (SNR)	A user-defined ratio that determines the split of the non-depleted irrigation water (NDW) to either society or nature. A ratio of 0.9 means society gets 90% of the NDW volume
Society (Soc)	One of the paracommoners obtaining water from the non-depleted water (NDW) adjusted by effecting agro-hydrological changes in the proprietor and the society-to-nature ratio (SNR)
Unused recovered flow/fraction (URF)	This percentage and volume of water that comes from the recovered flow (RF) from the proprietor system that is not used by the neighbour. It flows to nature and society either as a combined flow, or is subsequently apportioned to nature and society using the SNR
Time 1, baseline	The baseline scenario or ‘without changes’ which acts as a basis to calculate the changes arising in all future T2 scenarios
Time 2, scenarios	These are all future or T2 scenarios acting ‘with changes’
Total basin supply (TBS)	The TBS is the sum of the [Internal basin supply (renewable for both surface and groundwater) + External basin supply + Large scale dams supply + Fossil water supply + Desalinisation supply]. It becomes the basin water supply apportionable to all sectors, starting with the priority allocation to society and nature
Total zone potential carbon emissions and actual total zone carbon emissions	The TZPCE derives what would be the carbon emissions from the energy used if the latter were 100% derived from fossil fuels. It is the total sum of emissions for gravity, sprinkler and drip technologies. By applying the RES factor, the TZPCE is converted to actual carbon emissions

**Table 2:** Inputs for determining basin supplies and priority allocations.

Scenario		T1	Sc 2	Sc 3	Sc 4
**Supply sources**					
Internal basin supply (renewable groundwater)	hm^3^	87	73	73	73
Internal basin supply (renewable surface water)	hm^3^	25	21	21	21
External basin supply	hm^3^	165	134	134	134
Large-scale dams supply	hm^3^	0	0	0	0
Fossil water supply	hm^3^	90	50	50	50
Desalinisation supply	hm^3^	0	120	120	120
Total basin supply (& change)	hm^3^	367	398	398	398
**Municipal demand calculations**					
Population (millions)	millions	5.5	8.5	8.5	8.5
Gross per capita daily water use	l/c/day	87	100	100	100
Correction for non-revenue water losses	%	47%	25%	25%	25%
Net per capita daily water use	l/c/day	46	75	75	75
**Priority allocations non-agric**					
Priority domestic allocation (withdrawal)	hm^3^	174	310	310	310
Priority tourism allocation (withdrawal)	hm^3^	1	1	1	1
Priority industrial allocation (withdrawal)	hm^3^	40	45	45	45
Priority society allocation (withdrawal)	hm^3^	215	356	356	356
Priority water for nature allocation (net)	hm^3^	0	0	0	0
Total priority allocation (& change)	hm^3^	215	356	356	356
**Water consumption from priority allocations**					
Domestic process consumption	%	20%	20%	20%	20%
Tourism process consumption	%	80%	80%	80%	80%
Industry process consumption	%	80%	80%	80%	80%
Priority society process consumed (net use)	hm^3^	67	99	99	99
**Return flows from priority allocation**					
Society return flows	hm^3^	148	257	257	257
Society recovered return fraction (available)	%	10%	55%	55%	55%
Society recovered return fraction (available)	hm^3^	15	142	142	142
Society non-recovered fraction	hm^3^	133	116	116	116
Available basin supply (& change)	hm^3^	167	184	184	184

**Table 3:** Inputs for irrigation planning and efficiency hydrology.

Scenario		T1	Sc 2	Sc 3	Sc 4
Proprietor starting area (=primary zone area)	ha	17 000	17 000	14 000	18 930
Reference crop evapotranspiration	mm	1736	1813	1813	1813
Average areal crop factor	coeff.	0.50	0.50	0.50	0.50
Crop evapotranspiration	mm	868	907	907	907
Field ET reduction	coeff.	0.95	0.90	0.90	0.90
Deficit irrigation factor	coeff.	0.90	0.90	0.90	0.90
ETcrop actual	mm	742	734	734	734
Additional beneficial uses	mm	0	0	0	0
Rainfall	mm	202	95	95	95
Correction for effective rainfall	coeff.	0.80	0.80	0.80	0.80
Effective rainfall	mm	162	76	76	76
Informal suppl. water (not part of formal irrig withdrawals)	mm	0	0	0	0
Net irrigation req, depth equiv (from formal withdrawals)	mm	581	658	658	658
Gravity irrigation, % of total zone	%	0%	0%	0%	0%
Sprinkler irrigation, % of total zone	%	10%	10%	10%	0%
Drip irrigation, % of total zone	%	90%	90%	90%	100%
Gravity irrigation farm IE	%	40%	40%	40%	40%
Sprinkler irrigation farm IE	%	60%	70%	70%	70%
Drip irrigation farm IE	%	70%	85%	85%	90%
Average proprietor system CIE (=PZCIE)	%	69%	84%	84%	90%
Conveyance efficiency	%	95%	96%	96%	97%
Unit distribution efficiency	%	95%	96%	96%	97%
Field appl. eff. Set CIE, Ec, Ed so Ea not exceed 100%	%	76%	91%	91%	96%
Gross irrigation requirement, depth equiv	mm	841	788	788	731
Field application depth (& change)	mm	759	727	727	688
Final irrigation withdrawal rule		BIW	BIW	RIW	RIW
Final irrigation withdrawal	hm^3^	143.0	143.0	110.4	138.5
Final total irrigated area	ha	18 845	19 191	14 809	19 593

**Table 4:** Inputs for irrigation energy requirements and use of renewables.

Scenario		T1	Sc 2	Sc 3	Sc 4
Sprinkler net depth per irrigation dose	mm	40	40	40	40
Drip net depth per irrigation dose	mm	20	20	20	20
Sprinkler irrigation field app rate per hour	mm hr^−1^	7	7	7	7
Drip irrigation field app rate per hour	mm hr^−1^	2	2	2	2
Sprinkler area per pump	ha	50	50	50	50
Drip area per pump	ha	50	50	50	50
Sprinkler operating pressure	m	45	45	45	45
Drip operating pressure	m	15	15	15	15
Lift height from borehole—sprinkler	m	300	330	330	330
Lift height from borehole—drip	m	300	330	330	330
Renewable energy share—all irrigation types	%	30%	30%	65%	80%

**Table 5:** Key results including nexus gains and reversals.

Type of change over time	Unit	T1	Sc 2	Sc 3	Sc 4
Final total zone irrigated area	ha	18 845	19 191	14 809	19 593
Paper savings; change in field application depth	mm	759	−32.8	−32.8	−71.2
Paper savings; change in FAD volume over PrFA	hm^3^	129	+2.7	−27.4	+1.2
Paper savings; change in final withdrawals	hm^3^	143	0.0	−32.7	−4.6
Aggregate water depletion	hm^3^	136	139	108	136
Real savings; aggregate depletion change TZ	hm^3^	136	+3.1	−28.7	0.0
Real savings; aggregate depletion change TZ	%	136	+2%	−21%	0%
Real savings as nexus gain (+) or reversal (−)	hm^3^	136	−3.1	+28.7	0.0
Real savings as nexus gain (+) or reversal (−)	%	136	−2%	+21%	0%
Non-depleted water change	hm^3^	30	+13.7	+45.6	+16.9
Total zone crop BC change	hm^3^	140	+1.1	−31.1	+4.0
Total crop production	kt	350	352	272	360
Change total crop production (nexus gain if +)	kt	350	+2.7	−77.8	+10.0
Change total crop production % (nexus gain if +)	%	NA	+1%	−22%	+3%
Total zone carbon dioxide emissions	kt	151	166	64	45
Change in carbon dioxide emitted total zone	kt	151	+14.3	−87.5	−106.0
Change carbon dioxide emitted total zone %	%	NA	+9%	−58%	−70%
Carbon dioxide savings as gains (+) or reversals (−)	kt	NA	−14.3	+87.5	+106.0
Carbon dioxide savings as gains (+) or reversals (−)	%	NA	−9%	+58%	+70%
Water for nature	hm^3^	19.7	+28.7	+49.4	+30.7
Water for nature as % of total basin supply	%	5.4%	+7.2%	+12.4%	+7.7%
Water for nature; % points gains (+) or reversals (−)	%	NA	+1.8%	+7.0%	+2.3%

**Table 6:** Six final water disposition from priority water allocation and water conservation.

Scenarios	#1 Baseline		Sc#2		Sc#3		Sc#4	
Units	hm^3^	%	hm^3^	%	hm^3^	%	hm^3^	%
Proprietor IBC	98.7	27%	119.4	30%	92.2	23%	124.6	31%
Neighbour IBC	10.7	3%	6.9	2%	5.3	1%	4.4	1%
Society process cons.	78	21%	114	29%	125	31%	115	29%
Water for nature	19.7	5%	28.7	7%	49.4	12%	30.7	8%
Society non-recover.	133	36%	116	29%	116	29%	116	29%
Irrig. depl. losses	27.0	7%	13.2	3%	10.2	3%	7.4	2%
Total of fractions	367.0	100%	398.1	1.0	398.1	1.0	398.1	1.0

## Data Availability

All data that support the findings of this study are included within the article (and any supplementary information files).

## Footnotes

7 Fossil water is estimated as the difference between total water abstractions in the basin and the aquifers’ annual safe yield.
