# Supplementary material for: The iGains4Gains model guides irrigation water conservation and allocation to enhance nexus gains across water, food, carbon emissions, and nature
Source: Environ Res Food Syst. Author manuscript; Available in PMC 2025 Mar 15. (PMC7617495; doi:10.1088/2976-601X/adabe9)
Supplement: Supplementary Data [file EMS203569-supplement-Supplementary_Data.zip › erfsadabe9supp1.pdf]

Appendix A. Manual for the Excel model iGain4Gains
Dated 30 January 2025.

Citation: Lankford, B.A., Amdar, N, McCartney, M., Mabhaudhi, T. 2025. The iGains4Gains model guides irrigation water conservation and allocation to enhance nexus gains across water, food, carbon emissions, and nature. Environmental Research – Food Systems. https://doi.org/10.1088/2976-601X/adabe9

Table of Contents

1. Introduction, aims, abbreviations and definitions ... 2

1.1 Abbreviations in IGains4Gains ... 2
1.2 Definitions and explanations in iGains4Gains ... 5
1.3 The structure of the IGains4Gains Excel workbook ... 9

2. The conceptual model for iGains4Gains ... 9

3. Calculating, graphing and portraying nexus gains ... 12

4. The Excel model, preparing for data entry and understanding 'change' results ... 12

5. Fourteen stages to model four nexus gains ... 13

5.1 Stage 1. Establish and test multiple scenarios of saving and redistributions ... 13
5.2 Stage 2. Basin supplies, priority allocations and return flows ... 14
5.3 Stage 3. Set input variables and initial calculations for irrigation needs planning ... 15
5.4 Stage 4. Irrigation withdrawals and non-withdrawals; rules and calculations ... 16
5.5 Stages 5A and 5B. Set agro-hydrology ratios and analyse dispositions ... 16
5.6 Stage 6. The society-nature ratio for sharing the non-depleted water ... 17
5.7 Stage 7. Aggregate water depletion, aggregate depletion change, max real water saving ... 17
5.8 Stage 8. Redistribution of irrigation water between paracommoners ... 17
5.9 Stage 9. Derive other metrics ... 18
5.10 Stage 10. Five key dispositions from allocation and conservation ... 20
5.11 Stage 11. Pareto-checks on crop production ... 20
5.12 Stages 12A to 12D. Determine changes to energy requirements ... 20
5.13 Stage 13A to 13D. Carbon dioxide emissions and CO_2 savings ... 21
5.14 Further cross-checks ... 22

6. Stage 14 nexus gains results and graphs ... 22

7. Water allocation pie charts ... 23

8. Water distribution diagram ... 24

9. Data entry for the AZ Basin Case Study ... 26

9.1 Introduction ... 26
9.2 Establishing future scenarios ... 27
9.3 Setting out the supply-side and purposive water allocations ... 27
9.4 Establishing the irrigation requirements, efficiency hydrology and withdrawals ... 29
9.5 Establishing changes in energy use ... 31

10. References for this manual ... 32

1. Introduction, aims, abbreviations and definitions

This manual explains the Excel model 'iGains4Gains' which signals 'Irrigation efficiency gains for 4 nexus gains'. The Excel model given in Appendix B of the supplementary materials of this paper.

iGains4Gains is an accounting method or model for deriving water allocation, real water savings, crop production, carbon emissions and water for nature (and paracommons distribution of water), following water conservation in irrigated systems. iGains4Gains renames and builds on the Paracommons Water Accounting Model (PWAM) in: Lankford, B.A. and Scott, C.A. (2023). Lankford and Scott (2023) in turn built on this paper: Lankford (2023).

In addition to the two appendices A and B in the journal *Environmental Research – Food Systems,* readers are recommended to download the latest versions of the appendices from the website (https://www.irrigation.systems/) to follow the explanations and to explore the calculations in depth.

iGains4Gains demonstrates how four - positive or negative - nexus outcomes can be derived from a combination of both purposive water reallocation and water redistribution following an irrigation efficiency gain. The model has these steps and aims – which are described in more detail in this manual.

1. All computations are derived as occurring within a case or scenario and as changes over time from T1 (baseline) to T2 future cases.
2. It first determines basin water supplies from several sources to arrive at the total basin supply.
3. It then inputs the purposive priority water allocations for society (comprising domestic provision, industry and tourism) and nature. Society allocation decisions also set the society process depletion and recovered flows from society.
4. It allows model users to adjust irrigation planning variables and a modified version of water accounting (WA) to determine the distribution of basin water to different water fractions/dispositions following new irrigation efficiency, practices and infrastructure decisions (often taken with the aim of conserving water). iGains4Gains therefore models the effects of an irrigation efficiency gain.
5. It determines the net change in aggregate depletion as a result of changing water conservation practices. If this aggregate depletion (consumption) decreases over time, real water savings have occurred. This water saving is one of the four nexus gains.
6. It examines the effects of water conservation on the distribution of water between four interlinked paracommoners; the proprietor, its immediate neighbour, society and nature.
7. It pareto-checks whether changes in water conservation affect crop beneficial consumption and food production. This is the crop or food production nexus gain.
8. It determines the energy requirements of changes in irrigation technology from gravity to pressurised irrigation, and in turn calculates the carbon emissions when this energy is sourced from fossil and/or renewable sources. A saving in carbon emissions, meaning a reduction in carbon emissions, is a nexus gain.
9. It calculates how changes in purposive water allocation to nature and changes in irrigation hydrology result in water for nature. The same water or more water for nature is a nexus gain.
10. It acts as a scenario planning and dialogue aid. By selecting scenarios of different input choices, resulting in different distributions of gains and losses, it aims to support discussion of how water allocation and conservation can play out for the four paracommoners and the four nexus gains.

1.1 Abbreviations in IGains4Gains

Table 1 provides the abbreviations employed in IGains4Gains, while Table 2 below provides key definitions.

Table 1. Abbreviations in IGains4Gains

AAKc	Average areal crop factor (AAKc)
ABS	Available basin supply
ABSC	Available basin supply change
ABU	Additional beneficial uses
ADC	Aggregate depletion change
ADI	Aggregate depletion impact
ADITS	Aggregate depletion impact (total supply)
ADIAS	Aggregate depletion impact (available supply)
ADL	All depleted losses
AEIE	Aggregate EIE
AgHR	Agro-hydrology ratios
AID	Aggregate irrigation depletion
ALF	All loss fractions
AWD	Aggregate water depletion
BC	Beneficial consumption
BIW	Baseline irrigation withdrawal
CIE	Classical irrigation efficiency
cP	Correction for effective rainfall
CPA	Core priority allocation
CWP	Crop water productivity = WUE
DESAL	Water supply from desalinisation plants
DICA	Drip irrigation command area
CICEha	Drip irrigation carbon emissions per ha
DICIE	Drip irrigation farm IE
DIF	Deficit irrigation factor
DICPE	Drip irrigation potential carbon emissions
DPC	Domestic process consumption
Ea	Field application irrigation efficiency
Ec, Ed	Ec and Ed are the irrigation efficiencies for conveyance and distribution
EBS	External basin supply
EIE	Effective irrigation efficiency
ETc	Crop evapotranspiration
ETc act	Actual crop evapotranspiration
ETo	Reference crop evapotranspiration
FAD	Field application depth
FADC	Field application depth change
FETR	Field ET reduction
FOSIL	Fossil deep groundwater not or slowly renewed
FIW	Final irrigation withdrawal
FIWC	Final irrigation withdrawal change
FIWI	Final irrigation withdrawal impact
GICA	Gravity irrigation command area
GICEha	Gravity irrigation carbon emissions per ha
GICIE	Gravity irrigation farm IE
GICPE	Gravity irrigation potential carbon emissions
GIR	Gross irrigation requirement
GPCW	Gross per capita daily water use
IBC	Irrigation beneficial consumption
IBS	Internal basin supply (volume)
IBSGW	Internal basin supply – from groundwater
IBSSW	Internal basin supply – from surface water
IPC	Industry process consumption
ISW	Informal supplementary water
IWO	Irrigation withdrawal overplus
Ha	Hectares
Hm^3	Cubic hectometres (1 million cubic metres, MCM)
MRWS	Maximum real water saving
Nat	Nature (see also WfN; water for nature)

NBC	Non-beneficial consumption
NDWC	Non-depleted water change
NbA	Neighbour area
NbAC	Neighbour area change
NbFA	Neighbour final area = Reuse zone area (RZA)
NbALF	Neighbour all loss fractions
NbBC	Neighbour BC
NbBCC	Neighbour BC change
NbCIE	Neighbour CIE
NbNBC	Neighbour non-beneficial consumption
NbNRF	Neighbour NRF
NbOWBC	Neighbour other water beneficial consumption
NDW	Non-depleted water
NDWC	Non-depleted water change
NWW	Non-withdrawn water
NWWC	Non-withdrawn water change
NIR	Net irrigation requirement
NPCW	Net per capita daily water use
NRF	Non-recovered fraction
NRWL	Correction for non-revenue water losses
P	Rainfall
Pe	Effective rainfall
PeBC	Effective rainfall beneficial consumption
PDA	Priority domestic allocation (withdrawal)
PDAD	Proportion of PDA depleted
PIA	Priority industrial allocation (withdrawal)
PIAD	Proportion of PIA depleted
PNA	Priority nature allocation (net)
Pop	Population
Pr	Proprietor
PrALF	Prop all loss fractions
PrBC	Proprietor BC
PrCIE	Proprietor CIE
PrEIE	Proprietor EIE
PrEA	Proprietor expansion area = EZA
PrFA	Proprietor final area = PZA+EZA
PrFAC	Proprietor final area change
PrIBC	Proprietor irrigation beneficial consumption
PNA	Priority nature allocation
PrNBC	Proprietor non-beneficial consumption
PrNBCC	Proprietor non-beneficial consumption change
PrNRF	Proprietor non-recovered fraction/flow
PrOWBC	Proprietor other water beneficial consumption
PrSA	Proprietor starting area
PSA	Priority society allocation (withdrawal)
PSD	Priority society depleted (or net use)
PSPC	Priority society process consumed (net use)
PTA	Priority tourism allocation (withdrawal)
PIW	Provisional irrigation withdrawal
PZA	Primary zone area
RAM	Readily available moisture
RF	Recovered fraction
IGains4Gains	Irrigation gains for 4 nexus gains
RES	Renewable energy share - all irrigation types
RIW	Required irrigation withdrawal
RRF	Reused recovered flow/fraction
RRFC	Reused recovered flow/fraction change
RWW	Required water withdrawal
RWS	Real water saving

RZA	Reuse zone area = (neighbour area)
SICA	Sprinkler irrigation command area
SICEha	Sprinkler irrigation carbon emissions per ha
SICIE	Sprinkler irrigation farm IE
SICPE	Sprinkler irrigation potential carbon emissions
SNR	Society-to-nature ratio
SNRF	Society non-recovered fraction
Soc	Society
SRF	Society return flows (not depleted)
SRRF	Society recovered return flows
STOR	Large dams supply volume
T1	Time 1, baseline, before, without scenario
T2	Time 2, after, with, future scenarios #
TBS	Total basin supply
TBSC	Total basin supply change
TCP	Total crop production
TCPC	Total crop production change
TPA	Total priority allocation
TPAC	Total priority allocation change
TPC	Tourism process consumption
TZA	Total zone area (final) Equivalent to total aggregate area
TZAC	Total zone area change
TZCIE	Total zone classical irrig efficiency
TZCE	Total zone carbon emissions
TZCP	Total zone crop production
TZCPC	Total zone crop production change
TZCS	Total zone carbon savings as gains
TZDL	Total zone depleted losses
TZIA	Total zone irrigation area
TZIBC	Total zone irrigation BC
TZIBCC	Total zone irrigation BC change
TZOWBC	Total zone other water beneficial consumption
TZOWBCC	Total zone other water beneficial consumption change
TZPCE	Total zone carbon emissions potential, no correction for renewables
UDW	User-defined (irrigation) withdrawal
URF	Unused recovered flow/fraction
URFC	Unused recovered flow/fraction change
WEF	Water-energy-food
WfN	Water for nature
WfN%C	Water for nature % points change

1.2 Definitions and explanations in iGains4Gains

Table 2. Definitions and explanations in iGains4Gains

Terms	Definition
Additional beneficial uses	ABU, this is the water that is delivered by irrigation for other farming and cropping benefits such as water for leaching salts, for additional cooling of plants
Aggregate depletion change (ADC)	The change in total zone aggregate water depletion (AWD) from T1 to T2. A negative ADC indicates less depletion in T2 which represents a real water saving. A positive ADC is a rebound or increase in AWD. ADC = (AWD T2) – (AWD T1)
Aggregate depletion impact (ADI)	A percentage figure showing the impact of aggregate depletion from irrigation on the total basin supply. ADI% = AWD/TBS
Aggregate EIE (AEIE)	This is the effective irrigation efficiency of the beneficial consumption of whole irrigation system including the proprietor final area and neighbour area against all depleted water. AEIE% = TZIBC/(AWD)

Aggregate water depletion (AWD)	The AWD is the sum of depleted fractions across the proprietor and neighbour. AWD = PrBC + PrNBC + PrNRF + RRF (because it is assumed RRF is all depleted by the neighbour). Recall the proprietor in T2 can include the expansion zone command area
Agro-hydrology ratios (AgHR)	These are the user defined input ratios for the following PrCIE%, PrNBC%, PrNRF%, RRF%, URF%, NbCIE%, NbNBC% and NbNRF%. PrCIE% partitions FWW into BC and 'all loss fractions' (ALF). ALF distributes to the NBC, RRF, BRF and NRF
All depleted losses (ADL)	The ADL is the sum of the volumes for the NRF and NBC across the total zone. These losses are not recovered
All loss fractions (ALF)	The ALF is the sum of NBC, NRF and RF
Available basin supply (ABS)	The calculated volume of water that is allocatable to irrigation and between paracommoners after a correction of the total basin supply (TBS) minus the priority allocation of water.
Baseline irrigation withdrawal (BIW)	The BIW is the required irrigation withdrawal in the Baseline scenario. For T2 scenarios, the BIW might be carried forward depending on the RIW RULE
Beneficial consumption (BC), irrigation beneficial consumption (IBC) and other water beneficial consumption (OWBC)	This is the fraction of water beneficially consumed in crop evapotranspiration (ETc) for crop growth. This BC can be supplied from irrigation withdrawals (IBC) or other water such as wastewater or rainfall (OWBC). See non-beneficial consumption.
Classical irrigation efficiency (CIE)	Classical irrigation efficiency (CIE) is the ratio of crop beneficial evapotranspiration (BC) to the withdrawal water taken from headworks of the system (Seckler, 1996); CIE = BC/(BC+NBC+NRF+RF)
Crop water productivity (CWP)	Crop water productivity (= WUE) is the useful or economic crop yield per volume of water beneficially consumed in ET
Effective irrigation efficiency (EIE)	The ratio of crop beneficial evapotranspiration (BC) to the depleted fractions; EIE = BC/(BC+NBC+NRF) within the proprietor irrigation system or across the aggregate total irrigated system
Field application depth (FAD)	The field application depth is either depth equivalent (mm) or a volume (hm^3) and is derived from the net irrigation requirement corrected for the Ea%. The Ea % is assumed to apply to all zones of irrigation. The volume of FAD is computed from the depth x total zone area. A reduction in FAD is a paper water saving
Field application irrigation efficiency (Ea%)	Ea is the field level application irrigation efficiency to correct for losses when applying water from the field edge to the crop. It allows calculation of FAD in mm and hm^3 volume
Field ET reduction (FETR)	An adjustment factor that, alongside deficit irrigation, turns ETc into ETc act. FETR incorporates practices such as shade cloth, etc.
Final irrigation withdrawal (FIW)	The final irrigation withdrawal is important as it drives the agro-hydrology of the total zone and determines the non-withdrawal of water. It is the provisional irrigation withdrawal (PIW) corrected to be equal to or less than the available basin supply because the FIW cannot exceed the ABS
Final irrigation withdrawal impact (FIWI)	This is the impact of the proprietor's withdrawal on the available basin supply, where FIWI = FIW/ABS
FIW RULE	This line is where text is manually entered. Text 'BIW' inserts the baseline withdrawal from T1. Text 'RIW' inserts the required water withdrawal. Text 'UDW' inserts the user-defined water withdrawal
Fossil fuels and fossil groundwater	Fossil fuels are carbon-based; examples include coal, petrol and diesel. Fossil water is the term given to groundwater that is not being annually recharged
Gravity irrigation, drip irrigation and sprinkler irrigation (GI, DI and SI)	The model can calculate the impacts of changes in irrigation technology on irrigation efficiency, water consumption, energy use, and carbon emissions. Salient input variables include the percentage of command area under each technology and their irrigation efficiencies
Gross irrigation requirement (GIR)	This is the net irrigation water requirement corrected for the classical irrigation efficiency of the proprietor system. GIR = NIR/CIE
Informal supplementary water (ISW)	This tends to be local water not withdrawn from river basin supplies, but it supports crop growth. ISW comes from on-farm rainfall harvesting, small pond storage and wastewater reuse
Internal basin supply (IBS) and external basin supply (EBS)	The IBS is the water volume from within the catchment. The IBS is from renewable groundwater and renewable surface water. The EBS is the water volume imported from outside of the catchment (see also IBS)
Irrigation withdrawal overplus (IWO)	The IWO is the surplus water withdrawal calculated by comparing the final irrigation withdrawal with the required irrigation withdrawal for that scenario. An overplus arises if the final withdrawal is greater than the required withdrawal

Irrigation withdrawals (see FIW)	Irrigation withdrawals into an irrigation system are divided into four water fractions. These are; the beneficial consumption (BC) of water in crop evapotranspiration; non-beneficial consumption (NBC as evaporation); recoverable flows or fraction (RF); and non-recoverable flows (NRF) (Perry, 2011; Willardson et al., 1994)
Maximum real water saving (MRWS)	The MRWS is the maximum reduction in aggregate depletion across all scenarios. By identifying MRWS, discussions can examine what could be the maximum realisable water resulting from water conservation
Neighbour (Nb)	One of the paracommoners; a receiver of water discharging as drainage from the proprietor (receiving the RRF). The neighbour comprises the reuse zone if this is present
Neighbour BC (NbBC)	The percentage and volume of BC in the neighbour. It is derived from NbBC = CIE% x RRF
Neighbour CIE (NbCIE)	A user defined variable that sets the CIE of the neighbour system
Neighbour final area (NbFA) (RZA)	This is the irrigated area of the neighbour derived from a calculation of the NbBC volume divided by the depth equivalent of the net irrigation requirement (which is usually the same for the proprietor and neighbour)
Neighbour non-beneficial consumption (NbNBC% and NbNBC)	A user-defined percentage leading to a calculated volume.<br>The calculated volume is NbNBC = NbNBC% x RRF
Neighbour NRF (NbNRF% and NBNRF)	A user-defined percentage leading to a calculated volume.<br>The volume is NbNRF = NbNRF% x RRF
Non-beneficial consumption (NBC)	A water accounting fraction defining the amount of water depleted by evaporation that produces little or no crop production
Non-depleted water (NDW)	NDW is the water that is the sum of the water not-withdrawn into irrigation or is not depleted by the total zone of irrigation if it is withdrawn
Non-recovered fraction (NRF)	A water accounting fraction that defines the amount of water not recovered to other users in the basin
Non-withdrawn water (NWW)	Both a percentage and volume, this is the amount of available basin water allocatable to irrigation as a withdrawal, but is remaining after the final withdrawn water flows into the proprietor is calculated. NWW = ABS-FIW
Paracommons & paracommoners	A paracommons is a united system of water users, connected by agro-hydrological change in the proprietor irrigation system (main withdrawer and first user of water). Water (re)distribution occurs between four paracommoners; proprietor, neighbour(s), nature, and society. Multiple scenarios selected to assist discussions about desirable yet unpredictable outcomes
Priority allocation	This is the volume of water that is always made available to nature, domestic use, tourism and industry (= society) and is the first priority allocation made before any irrigation calculations of withdrawals, depletions and distributions
Priority society process consumed (net use)	This is the water beneficially consumed within society and is the sum of the process consumption within each of the domestic, tourism and industry sectors
Proprietor BC (PrBC)	The proprietor’s beneficial consumption as a volume
Proprietor CIE (PrCIE%)	The proprietor’s classical irrigation efficiency
Proprietor EIE (PrEIE)	The proprietor’s effective irrigation efficiency
Proprietor expansion area	Equivalent to the expansion zone, the proprietor expansion zone occurs when in T2 scenarios more water is withdrawn than is required (delivering an irrigation withdrawal overplus)
Proprietor final area (PrFA)	The proprietor’s final area once re-calculated = PZA+EZA
Proprietor non-beneficial consumption (PrNBC% and PrNBC)	The proprietor’s NBC as a percentage and volume
Proprietor non-recovered fraction/flow (PrNRF% and PrNRF)	The proprietor’s NRF as a percentage and volume
Proprietor starting and final area (PrSA, PrFA)	The PrSA is proprietor’s starting area in hectares. It is an independent user defined variable. The final area of the proprietor may differ from the starting area because an irrigation withdrawal overplus may arise which allows the proprietor to expand into an expansion zone. Thus PrFA = PZA+EZA. The final area of the proprietor is derived from a calculation of the PrBC volume divided by the depth equivalent of the net irrigation requirement (which is usually the same for the proprietor and neighbour)
Rainfall (P), effective rainfall correction (cP) and effective rainfall (Pe)	User defined inputs from agro-meteorological data convert the seasonal rainfall depth to an effective rainfall figure
Real water saving (RWS)<br>Realisable water overplus (RWO)	The RWS is equivalent to the aggregate depletion change when the latter is negative. In other words, aggregate depletion goes up there is a rebound in depletion across all three irrigation zones

Recovered fraction (or flows) (RF)	RF is the fraction of water that is recovered and is either consumed by the neighbour (RRF) or is unused and so flows to nature and society (URF). This split of the RF is defined by user defined inputs for RRF% and URF%
Renewable energy share (RES)	Renewable energy share corrects the potential carbon emissions from energy use in irrigation because RES factors in how much of the energy comes from non-carbon nuclear or renewable energy. RES applies to the total zone and all irrigation types. TZCE = TZPCE * (1 – RES)
Required irrigation withdrawal (RIW)	The RIW is the required water withdrawal provided new (T2) water conservation practices reduce the NIR and GIR that in turn should reduce the water abstracted at the headworks in accordance with the new GIR. The RIW may not be withdrawn however depending on the FIW RULE
Reused recovered flow/fraction (RRF)	Part of the recovered fraction as a percent and as a volume consumed by the neighbour. Recall all RRF is depleted as it is assumed no further recovered flows issue from the neighbour
Society (Soc)	One of the paracommoners obtaining water from the non-depleted water (NDW) adjusted by effecting agro-hydrological changes in the proprietor and the society-to-nature ratio (SNR)
Society nature ratio (SNR)	A user-defined ratio that determines the split of the non-depleted water (NDW) to either society or nature. A ratio of 0.9 means society gets 90% of the NDW volume
Society return flows (SRF)<br>Society recovered return fraction (SRRF)<br>Society non-recovered fraction (SNRF)	The society return flows is the difference between the Priority society allocation (withdrawal) and the priority society process consumed (net use). The return flows are then split into the recovered return fraction (SRRF, which can be reused within the basin) and the non-recovered fraction (SNRF which is either unavailable for use in the basin or flows out of the basin)
Time 1, baseline	The baseline scenario or 'without changes' which acts as a basis to calculate the changes arising in all future T2 scenarios
Time 2, scenarios	These are all future or T2 scenarios acting 'with changes'
Total basin supply (TBS)	The TBS is the sum of the [Internal basin supply (renewable) + External basin supply (renewable) + Large scale dams supply + Fossil water supply + Desalinisation supply]. It becomes the basin water supply apportionable to all sectors, starting with the priority allocation to society and nature
Total basin supply (TBS)	The TBS is the sum of the [Internal basin supply (renewable) + External basin supply (renewable) + Large scale dams supply + Fossil water supply + Desalinisation supply]. It becomes the basin water supply apportionable to all sectors, starting with the priority allocation to society and nature
Total final irrigated area (TFIA)	This is the sum of the final proprietor and neighbour areas (ha)
Total irrigation BC (TIBC)	This is the final irrigation BC from the proprietor and neighbour areas
Total zone potential carbon emissions and actual total zone carbon emissions	The TZPCE derives what would be the carbon emissions from the energy used if the latter were 100% derived from fossil fuels. It is the total sum of; gravity irrigation potential carbon emissions (GPCE); sprinkler irrigation potential carbon emissions (SPCE) and drip irrigation potential carbon emissions (DPCE). By applying the RES factor, the TZPCE is converted to actual carbon emissions
Unused recovered flow/fraction (URF)	This the percentage and volume of water that comes from the recovered flow (RF) from the proprietor system that is not used by the neighbour. It flows to nature and society either as a combined flow, or is subsequently apportioned to nature and society using the SNR
Water for Nature (WfN)	Water for nature is one of the nexus gains in the model as measured by the difference in percentage points of total basin supplies allocated to nature moving from the T1 % share to T2 % share. Water for nature can both be purposively allocated water from total basin supplies and is one of the paracommoners obtaining water from the non-depleted water (NDW) adjusted by effecting agro-hydrological changes in the proprietor and the society-to-nature ratio (SNR). Water for nature covers not just environmental flows but water that provides ecological benefits, such as small ponds and shallow water tables

1.3 The structure of the IGains4Gains Excel workbook

There are nine worksheets in the iGains4Gains model as explained in Table 3:

Table 3. The nine worksheets in the Excel model

#	Sheet name	Explanation
1	Introduction	Introduction. General introduction to the model and its objectives
2	Quick start guide	Quick start guide and explaining some possible ways of using the model
3	Inputs 4 scenarios	The 'Inputs 4 scenarios' sheet uses four scenarios to explore different scenarios of future nexus gains
4	Stage 14 Nexus metrics	This sheet compiles the main relevant results from the previous 'Inputs 4 scenarios' sheet
5	Nexus gains graphs	This sheet contains different graphs of the nexus gains
6	Tables 1 to 5	This sheet creates tables 1-5 for copying and pasting into Word
7	Piecharts & table 6	This sheet contains Table 6 and pie-charts of the distribution of water between different sectors and paracommoners
8	Redistrib 4 figs	This generates four spatial distributions of water between paracommoners after water conservation in the proprietor system
9	Notes, maps	This sheet contains other relevant information e.g. maps or supplementary data

2. The conceptual model for iGains4Gains

iGains4Gains builds on two models, ISA and PAWM, created by one of the authors (Lankford, 2023; Lankford and Scott, 2023). Readers are guided to those two articles and models for further information about the evolution of the models. iGains4Gains is a new model that takes the relative simplicity of PAWM, brings in some of the additional detail of ISA and adds further details and calculations on total basin supplies, purposive water allocation, and carbon emissions.

The three diagrams that explain the approach of iGains4Gains are given in **Figures 1 to 3.** F**igure 1** introduces our task and approach. This task is framed as a single question; “Can changes in water allocation and irrigation management in irrigated river basins deliver water-energy-food nexus gains?” To answer this, we developed an Excel model called iGains4Gains. This model combines purposive water allocation decisions and the redistribution of water based on irrigation efficiency improvements across three zones (primary, expansion and reuse) of irrigated systems located within a river basin/aquifer to assess nexus gains. It compares selected nexus metrics in T2 (after) scenarios with the same metrics from a T1 baseline case (before). **Figure 1** presents a process that starts in the top left-hand corner, where decisions about priority basin water allocation are made (blue box). These determine how much water is withdrawn into the irrigated systems of the basin. The next step (white box) applies irrigation/hydrology variables and calculations (e.g. irrigation efficiency and effective rainfall) to these withdrawals, resulting in the irrigation systems' water, crop and energy accounts (grey box). The model then reconstitutes the different flows from the priority allocations and irrigation hydrology to derive interim basin water allocations and dispositions, including; the amount of water beneficially consumed in crop production; the energy used within, and carbon emissions, from irrigation technologies, such as sprinkler or drip; and the proportion of the water allocated to nature. These interim accounts are then employed to calculate the four nexus gains; water savings, changes in crop production, savings of carbon emissions and changes to water for nature.

Thus, summarising, moving from left to right in Figure 1, the modeller selects user-defined (independent) water allocation and irrigation hydrology variables to derive metrics and graphs of nexus gains or reversals. User-defined selections are usually estimates guided by existing data, experience, and literature sources, including textbooks, stakeholder dialogue, and new research. Some variables (e.g. total command area under irrigation) can be relatively accurately determined from satellite imagery. Other variables (e.g. irrigation efficiency) are often difficult to measure across time and space, and are informed by experience, estimates and fieldwork.

Figure 1. The iGains4Gains determination of four nexus gains in an irrigated river basin

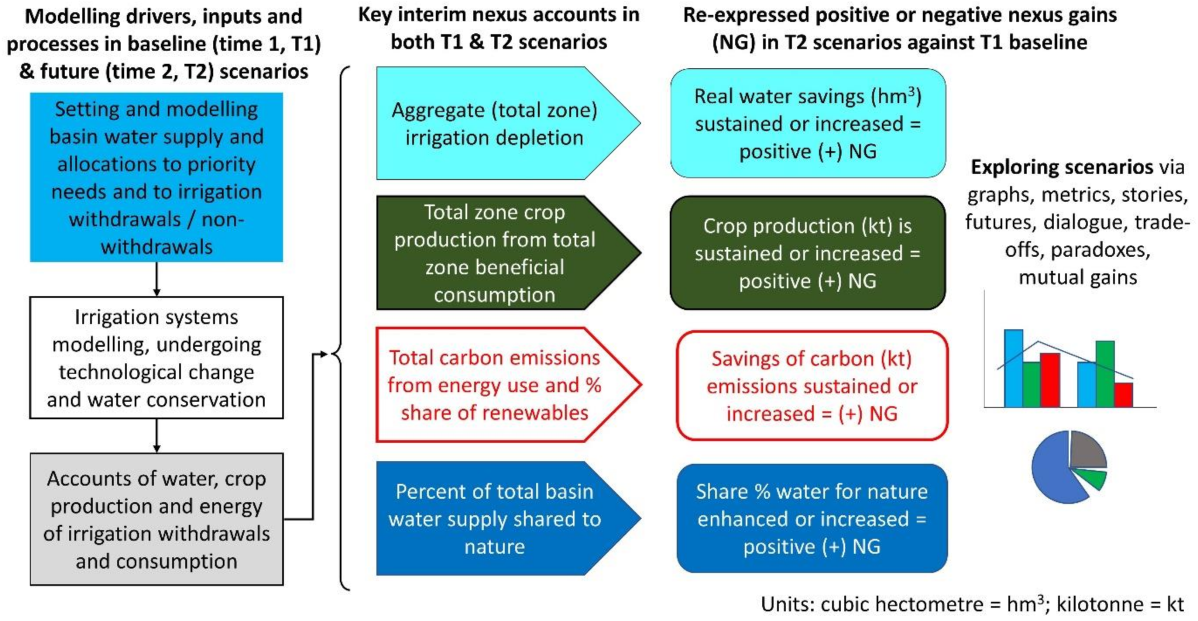

Figure 2. The five main steps in deriving the nexus gains of an irrigated river basin

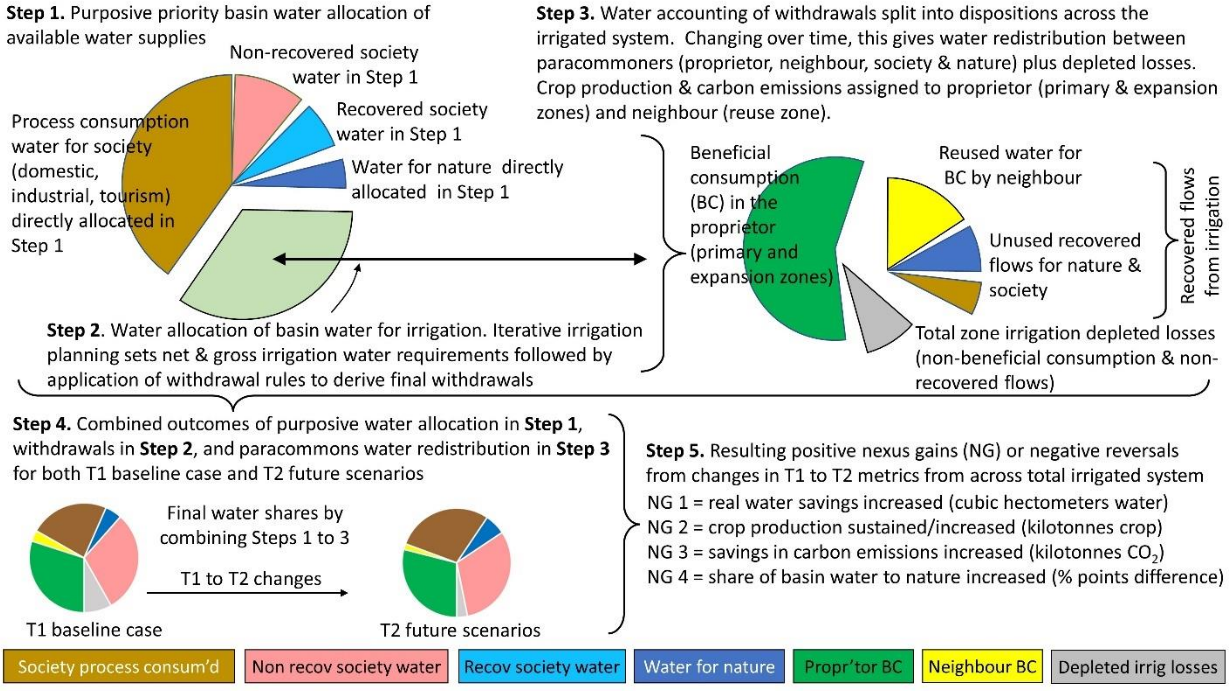

Building on **Figure 1**, **Figure 2** presents an overview of the iGains4Gains model broken down into five steps (the model comprises 14 stages, explained below). The top lefthand side of the diagram presents the first step of purposive priority water allocation. These priority allocations result in water for society (domestic, industrial and tourism use) and water for nature. The second step establishes the water allocation for agriculture as a final withdrawal volume, derived from the net and gross irrigation requirements adjusted by some withdrawal rules for both T1 and T2 scenarios. In the third step, in the top right-hand side of the diagram, the volume withdrawn is fractionated by irrigation planning variables into water accounting dispositions (e.g. beneficial consumption, non-beneficial consumption, etc) within three zones of the irrigated system (primary, expansion and reuse). These dispositions, zones of irrigation and their areal extent can then be linked to changes per-zone and to total crop production and carbon emissions. The

fourth step, at the bottom of the diagram, is the combination of results from steps 1 to 3, namely purposive water allocation, irrigation withdrawals and the management of irrigation water. These are calculated as changes over time from T1 to T2. For example, water for nature is derived from changes to initial purposive allocation and changes to return flows or savings within irrigation. In the fifth and final step, our model calculates how these changes generate four nexus outcomes expressed as gains: 1) water savings boosted (depletion is reduced across the total irrigated system); 2) crop production is sustained or boosted; 3) carbon emissions are saved (reduced), and 4) water for nature is sustained or increased.

Figure 3. Block diagram of water flows and dispositions in the iGains4Gains model.

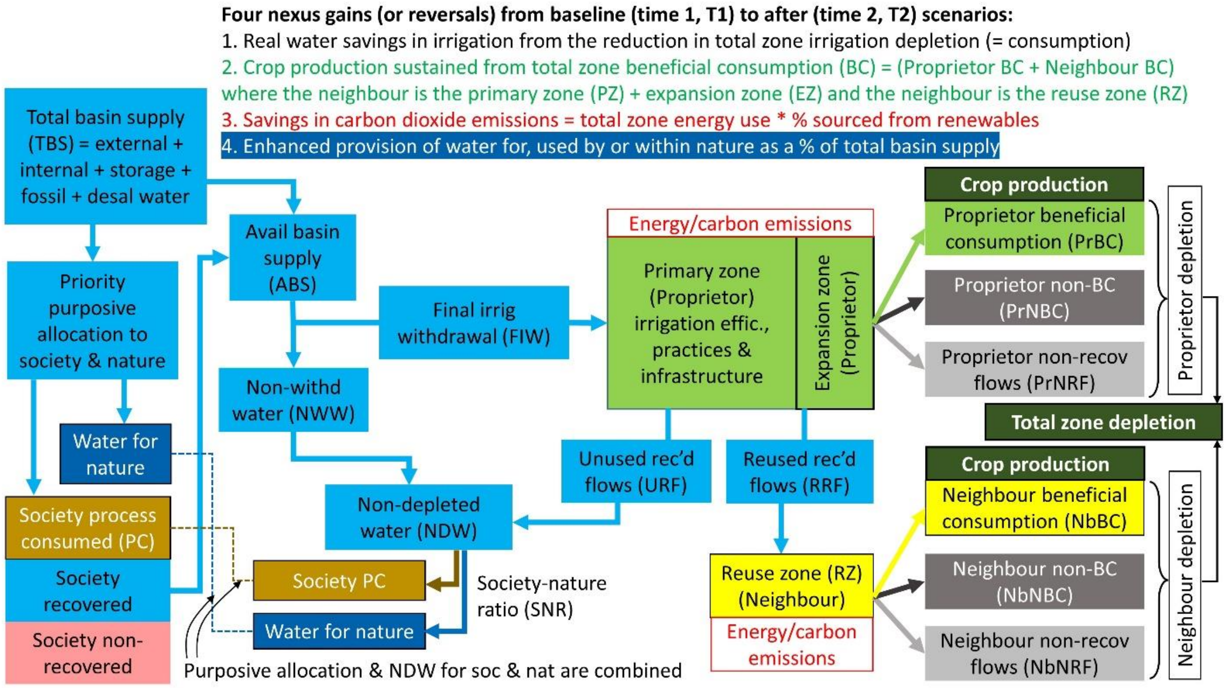

Of the three introductory figures, **Figure 3** is the most instructive. It is a block diagram showing water supplies in a river basin divided via water allocation and irrigation water conservation to six different dispositions. The mathematical comparison of selected inputs and outputs contained changes over time that allow the nexus gains to be calculated. **Figure 3** contains these elements:-

1. The river basin supplies and allocations are mainly coloured light blue. These cover the total basin supply, the available basin supply, the water withdrawn into irrigation, its non-withdrawal and return flows from irrigation. The latter two give the remaining non-depleted water that is available for nature and society.
2. First is the determination of the total priority first-in-line allocation for society and nature. From society's allocation are return flows that either exit the basin and are no longer part of the available basin water supply (in pink), or that feed into the available basin water supply (light blue).
3. Coloured brown and dark navy blue are the two water uses by society and nature respectively. Their total volume of water is the water from priority allocation plus that which is not withdrawn and not depleted by irrigation. The latter is split to society and nature via a society-nature-ratio.
4. Coloured green, the first-use withdrawal of water into the proprietor irrigation system (also called the primary zone). This generates a distribution of water to different irrigation fractions/dispositions depending on irrigation efficiency, practices and infrastructure. Also coloured green is the expansion zone of the primary zone that can occur in T2.
5. Coloured yellow is the reuse of recovered flows which generates water and beneficial consumption for the neighbour (equivalent to the reuse zone).
6. Coloured dark and light grey are irrigation's depleted losses, namely non-beneficial consumption and non-recovered flows respectively. In other diagrams there are a single grey colouring.

7. Coloured red, is the calculation of the total carbon dioxide emissions from irrigated agriculture under changing practices. This is a function of the energy use across the total zone factored by the source of that energy. If the source is from fossil fuels it leads to carbon dioxide emissions but if it from renewables (and nuclear) it is assumed to be emissions-free. A nexus gain occurs when savings of carbon emissions are achieved.

3. Calculating, graphing and portraying nexus gains

Before the model's working is explained in the next section, the subject of nexus gains is introduced. It is helpful to know at this point what the main objective of the model is, meaning what is the conceptual reasoning behind, and mathematical calculation and graphing of, the four nexus gains. The nexus gains are so important that they are placed prominently in the model in various locations. For example they are placed at the very top of the worksheet called '***Inputs 4 scenarios',*** plus they have their own worksheet '***Nexus gains metrics'*** and their own graphs in '***Nexus gains graphs'***. There are four nexus gains which are derived from changes between T1 (baseline) and T2 scenarios. The four nexus gains are:

1. **Real water savings achieved or increased**. Real water savings are derived from reduction in total zone or aggregate irrigation depletion (synonymous with consumption). Savings occur from a decrease in aggregate depletion over time, so a nexus gain is when real water savings occur or increase (thus when depletion in T2 is less than depletion in T1). A nexus reversal is when real water savings do not occur or decrease (when aggregate depletion increases or rebounds over time)
2. **Same or higher crop production**. A nexus gain is when total crop production is sustained or increased in T2 compared to the T1 crop production. This occurs when total zone beneficial water consumption in T2 from the total zone (Proprietor + Expansion + Neighbour BC) remains the same as T1 or is higher than T1. The cross-check of the changes in aggregate depletion with crop production is the pareto check commonly referred to in the literature (Pérez-Blanco et al., 2020).
3. **Savings in carbon dioxide emissions**. A nexus gain occurs when T2 irrigation carbon emissions are reduced in T2 as compared to T1. Emissions come from total zone energy use in irrigation factored by the percentage of energy sourced from renewables. This calculation also acts as a pareto check that the act of conserving water by adopting new irrigation technology does not leave others worse off by increasing carbon emissions.
4. **Water for Nature sustained or boosted**. A nexus gain in water for nature occurs when in T2, the amount of water within or for nature as a percentage of total basin supply is either sustained increased. This fourth gain could be seen as another way of expressing 'real water saving' but it gives weight to the purposive allocation of water from total basin supplies and the redistribution of water to nature as a paracommoner following water conservation in the irrigated system. Therefore, arguably this provides another pareto check on whether water conservation in irrigation harms or helps nature.

4. The Excel model, preparing for data entry and understanding 'change' results

To follow the explanation below, it is useful to access the worksheet '***Inputs 4 scenarios'*** where the logic and content of the calculations is clearer. Equations are simplified and omit corrections for converting area, depth and volumes. (Recall when converting depth equivalents (mm) to volumes (cubic hectometres) an area-depth-volume correction is also applied knowing 1 mm of water on 1 hectare is 10 cubic metres, and that one cubic hectometre contains a million (1,000,000) cubic metres).

In the worksheet the independent variables (e.g. annual rainfall, irrigation efficiency or the correction for non-revenue water losses) are shown in orange to help with data entry. The values for these independent variables is best done iteratively by referring to a number of sources. These sources cover, best-guesses, experimental and gauged data, published statistics, accepted regional norms and practices, or to numbers derived from active discussions, e.g. in a workshop setting, to clearly separate out scenarios. Where these data strongly contradict each other (e.g. modelled versus gauged return flows), the modeller(s), should discuss which to adopt or to adapt and their reasoning.

To reflect their own river basin case studies more accurately, modellers are welcome to alter the composition and order of calculations, or to delete or add new calculations. An example of such a change might be the way return flows from 'society' are further divided into recovered within the basin, recovered outside of the basin or are not-recovered anywhere. These additional edits would alter the rows of the calculations and therefore any changes need to be carefully traced for their knock-on effects elsewhere in the spreadsheet.

The model iGains4Gains is specifically designed to derive 'change over time' results. Therefore, unless otherwise explained, all the calculations below are conducted for all T1 and T2 scenarios. Note however, that some T2 cells reference data in T1 (baseline) cells (e.g. the text 'BIW') according to the Withdrawal RULE set. Furthermore, all the 'change' (delta) calculations are derived by subtracting T1 value from the T2 value. For example, the aggregate depletion change (ADC), where ADC = AIDT2 – AIDT1. A negative result means less depletion.

Recall, pressing F2 in Excel in each cell will bring up the cell's calculations.

5. Fourteen stages to model four nexus gains

There are fourteen stages in the Excel model which help separate out different parts of the model and provide a more accessible breakdown of per-stage objectives. **Figure 4** draws the 14 stages onto the block diagram of **Figure 3** to explain how each part sits within the model as a sequence of decisions, noting that users will usually operate the model iteratively.

Figure 4. The 14 stages of the iGains4Gains model using Figure 3

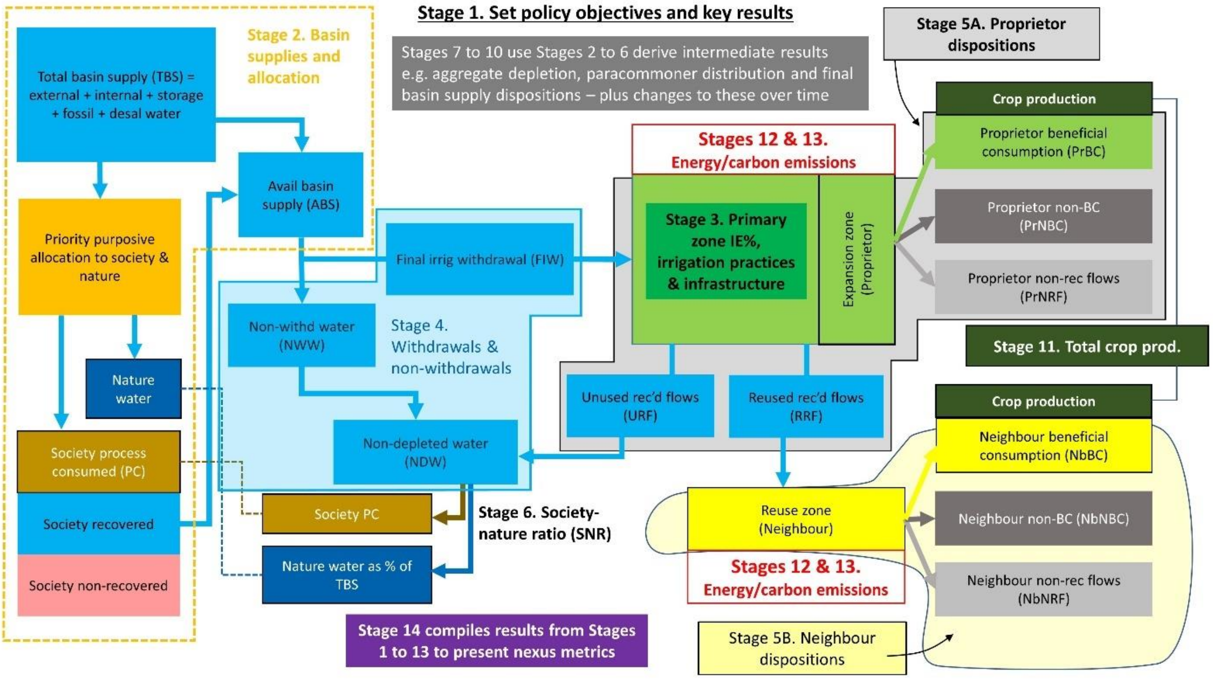

5.1 Stage 1. Establish and test multiple scenarios of saving and redistributions

This first stage establishes how multiple scenarios reveal future policy alternatives for discussion. Therefore Stage 1 sets the contrasting stories of nexus gains and reversals. For example, options for setting up and testing scenarios include:

- Deciding what year in the future represents a target date; e.g. in 25 years' time.
- Deciding what the three future T2 scenarios should represent in terms of pathway narratives or discussion points. For example, it might be relevant to demonstrate a rebound in water consumed, or some real water savings, or show how the nexus outcomes are interconnected. Options include;
    - Observing how changes in the irrigation efficiency ratios give rise to new distributions.

- Maximising the beneficial consumption and/or area under irrigation
- Maximising the volumes flowing to nature and society
- Balancing the gains in volumes partitioned between irrigation, nature and society
- Showing that changes within the proprietor often impact the neighbour's agro-hydrology. In other words, the neighbour's BC is rarely protected at the same level as T1.
- Examining what factors give rise to the largest possible real water saving, termed a maximum real water saving (MRWS).

In the Excel model, Stage 1 also presents the key headline nexus results at the top of the spreadsheet.

5.2 Stage 2. Basin supplies, priority allocations and return flows

The aim of Stage 2 is threefold; 1) to determine the total available supply (TBS) from different sources; 2) to specify the priority allocations that are secured prior to withdrawals for irrigation; and 3) to determine the remaining balance termed the available water supply (ABS). Stage 3 starts by adding up six types of water supply which make up the total basin supply. These are the; internal basin supply (renewable groundwater); internal basin supply (renewable surface water); external basin supply (renewable); large-scale dams supply; fossil water supply; and desalinisation supply. Each catchment might have its own norms for determining how much surface and groundwater storage falls within external and internal renewable water supply versus how much surface and groundwater storage stems from interseasonal large scale dams and the use of deep non-renewed fossil groundwater. It is important to discuss and clarify these norms otherwise water volumes will be omitted or double-accounted.

The following steps itemise these decisions and the resulting total.

1) Set the external and internal basin supply figures (EBS and IBS, hm^3) for the relevant time period or season. Note that the internal basin supply is derived from the internal renewable supply for groundwater (GW) and surface water (SW). The external supply is that which is transferred in by artificial means or, more rarely, arrives via a form of natural underground.
2) Set the large-scale storage, fossil groundwater and desalinised water supply figures (STOR, FOSIL and DESAL, hm^3) for the relevant time period or season.
3) Adding these inputs gives the total basin water supply (TBS, hm^3)

Then the core priority water withdrawals for domestic, industry, tourism and nature much be set and accounted for. These needs must be met prior to any water being made available to all sectors including irrigated agriculture. Society needs begins with a calculation of the domestic water supply from the population in the basin and per capita daily household water use. Amounts of water for industry and tourism are then added to arrive at the total 'society' supply. Water for nature is then entered. The following steps, for both T1 and T2 cases, itemise these decisions and the resulting calculations:

1) Enter the catchment human population in millions for the relevant scenario.
2) Enter the net daily per capita water use at the household level in litres per day.
3) Enter the correction for non-revenue water losses.
4) The model uses the non-revenue losses correction to calculates the gross daily per capita water use at the household level in litres per day. For example, if losses entered are 20% then the correction to the net per capita use is adjusted by 120%.
5) iGain4Gains then calculates the priority domestic allocation (PDA) from the population and gross per capita water use. By multiplying this by 365 days, the model calculates the annual withdrawal for domestic use in hm^3.
6) Set the priority tourism allocation (PTA) as a user-defined input to the model in hm^3. The model separates tourism from domestic provision because in some basins this can be very large, and tourism facilities can use a lot of water (from hotels and swimming pools).
7) Set the priority industry allocation (PIA) as a user-defined input to the model in hm^3.
8) The model then calculates the sum of PDA, PTA and PIA as PSA (priority society allocation).
9) Set the priority nature allocation (PNA) as a user-defined input to the model.
10) The model then calculates the total priority allocation as the total of PNA + PSA.
11) Then enter three selections for the percentage of process-consumed water of the domestic, tourism/leisure and industrial withdrawals. Process-consumption is the beneficial consumption of

these three sectors. These three decisions allows the Excel model to determine the volume of beneficially consumed (depleted) water by society (PSPC), from domestic, tourism and industrial depletion respectively.

12) Note there is no separate entry and calculation of water depleted by nature. In other words, all water for nature is process water (and thus supports ecosystem services), regardless of whether it is depleted or not.
13) The model then calculates the return flows from society (SRF) as a function of society withdrawals minus process society consumption.
14) From these society return flows the model needs to determine how much is no longer available for the basin and how much is available for reuse. To determine these dispositions, the user enters the percentage of return flows that are recovered (SRRF, this water is available for reuse in the basin). The model also returns this recovered fraction as a volume.
15) The latter step simultaneously determines the volume of non-recovered return flows (SNRF) that are no longer available for use within the basin. SNRF is one of main six dispositions of the model (shaded pink).
16) The model then calculates the available basin supply (ABS) as the total basin supply (TBS) minus the priority nature allocation minus the water process consumed in society minus the society non-recovered flows. Thus ABS = TBS – PNA – PSPC – SNRF.

5.3 Stage 3. Set input variables and initial calculations for irrigation needs planning

Stage 3 sets irrigation planning factors that establish the irrigation water withdrawals given Stage 4. Stage 3 utilises user-defined inputs described under irrigation planning for determining irrigation water requirements (FAO, 1999). Recall inputs and calculations are conducted for both the baseline (T1) and after (T2) cases or scenarios.

A. Select nine input variables for the proprietor system for the whole season. These are the;
    1. Proprietor starting area irrigated (PrSA, ha). Note this starting area is not the same as the final area as the latter changes depending on expansion of the proprietor (and reuse by the neighbour also contributes to the final irrigated area).
    2. Reference crop ETo (ETo, mm per season)
    3. Average areal crop factor (AAKc). Note the AAKc allows the modeller to account for changes in cropping patterns for the whole season or year.
    4. Field ET reduction (FETR, as a factor) that reflects steps taken to reduce ET, such as shade cloth and soil mulches.
    5. Deficit irrigation factor (DIF) which allows for deficit irrigation.
    6. Additional beneficial uses (ABU as mm per season), covering for example, salinity control, crop cooling etc.
    7. Rainfall (P) for the season.
    8. The correction for effective rainfall (cP) to determine effective rainfall (Pe). Note, soil management such as ridging and mulching can improve the extent to which rainfall is better captured.
    9. Informal supplementary water (ISW as mm per season). These are soil water additions that are not supplied by formal water withdrawals. Examples include local farm water from boreholes, rainwater harvesting, shallow watertables and non-conventional wastewater. These volumes may be assisted by the presence of on-farm water storage (ponds).
B. The areal proportions of the irrigation efficiencies for each type of technology need to be set. These are user-defined and although are set for the proprietor, also apply as percentages to the total aggregate zone. The coverages are; gravity irrigation command area as % of total zone; sprinkler irrigation command area as % of total zone; and drip irrigation command area as % of total zone.
C. The irrigation efficiencies for the three types of technology are then set. These are; gravity irrigation farm %IE; sprinkler irrigation farm %IE; and drip irrigation farm %IE.
D. From the latter two steps and their inputs, the average proprietor and total zone classical irrigation efficiency (CIE%) values are determined by the model.
E. With the CIE set, the conveyance efficiency (Ec) and unit distribution efficiency (Ed) then need to be set. The three data points of the CIE, Ec and Ed, then allow the model to calculate the field application efficiencies (Ea%). The Ec, Ed and Ea efficiencies apply to all irrigated zone areas.

The field application efficiency is important for calculating changes in field application depths which are a measure of paper water savings.

From the above inputs, iGains4Gains calculates the following:

1) The crop evapotranspiration ETc = ETo x AAKc
2) The actual crop evapotranspiration, ETc act (mm) = ETc x FETR x DIF.
3) The corrected effective rainfall, Pe = P x cP
4) The net irrigation requirement (NIR) where NIR (mm) which must account for any surplus of rainfall plus informal supplementary water over the need for water for 'ETc act' plus additional beneficial uses, which would mean irrigation is not needed. Thus the equation is NIR = if [(Pe + ISW) > (ETc act + ABU), 0, (ETc act + ABU – Pe – ISW)]
5) The field application efficiencies (Ea%) from Ea% = CIE/(Ec x Ed)
6) The gross irrigation requirement (GIR) where GIR (mm) = NIR/CIE%.
7) The field application depth (FAD, mm) where FAD = NIR/Ea% and its change from T1 to T2.

5.4 Stage 4. Irrigation withdrawals and non-withdrawals; rules and calculations

This stage uses Stage 3 results and some withdrawal decisions to determine the final irrigation withdrawal (FIW) and the residual non-withdrawn water (NWW). The non-withdrawn water is a necessary part of the calculations of water accounting in irrigation as it determines the amount of water for non-irrigation sectors by not being withdrawn into and thus depleted by or returned by the irrigation system.

1) The required irrigation withdrawal (RIW, hm^3) is calculated by the model by referring to the new gross irrigation requirement and proprietor starting area from Stage 3. Thus RIW = GIR x PrSA x (area-depth-volume correction).
2) Next the baseline irrigation withdrawal (BIW, hm^3) is calculated and applied to all scenarios. The baseline withdrawal is always the required irrigation withdrawal (RIW) for the T1 baseline scenario. The T1 BIW therefore references the T1 RIW. For all T2 scenarios, the T2 BIW cell references the T1 BIW value.
3) A row is then reserved for a user-defined withdrawal (UDW) should this be necessary, for example when exploring the impact of different withdrawals on irrigated area and nexus gains.
4) Then the FIW RULE text is entered by typing in one of three options; 'BIW', 'RIW' and 'UDW'. These respectively instruct the spreadsheet to reference either the baseline withdrawal, the required withdrawal or the user-defined withdrawal when determining the provisional water withdrawal.
5) The provisional irrigation withdrawal (PIW) is then determined using an IFS statement allowing the model to select the correct withdrawal by referring to the text entered into the FIW RULE cells. An IFS statement is a three-way IF statement. Thus if the rule text is 'BIW', then PIW returns the BIW value, and so on, depending on the text in the FIW RULE cells.
6) Next the provisional withdrawal is converted to the final irrigation withdrawal using an Excel two-way 'IF statement' so that if PIW exceeds ABS, then ABS becomes the FIW and if not, then the PIW becomes the FIW. The IF equation is FIW=IF(PIW>ABS,ABS,PIW).
7) The final irrigation withdrawal impact (FIWI, %) is calculated, where FIWI = FIW/ABS as a measure of how much of the available basin supply is withdrawn in percentage terms.
8) The irrigation withdrawal overplus (IWO, hm^3) is then calculated by the model. The IWO is the surplus withdrawal calculated by comparing the final irrigation withdrawal with the required irrigation withdrawal within each scenario. IWO = FIW–RIW. However, an IF statement operates to return zero if FIW is less than RIW. Thus the equation is IWO = IF(FIW>RIW,FWW–RIW,0).
9) The non-withdrawn water (NWW, hm^3) is calculated as a volume; NWW = ABS–FWW.
10) The non-withdrawn water (NWW, hm^3) is calculated as a percentage of ABS = (1 – FIWI%).
11) In the second 'change' column (to the right of each scenario), the changes in TBS, ABS, FIW and NWW are calculated (each by subtracting the baseline T1 values from the new T2 values).

5.5 Stages 5A and 5B. Set agro-hydrology ratios and analyse dispositions

Stage 5 uses the agro-hydrology ratios (AgHR) to apportion the withdrawn water into different fractions/dispositions. Stage 5 begins with two automatic calculations:

1) Recall, the proprietor CIE has already been set in Stage 3 above, and thus Stage 5 returns this automatically to calculate the volume of the proprietor beneficial consumption (PrBC, hm^3) from the volume of the FIW, where PrBC = (FIW*CIE%).

2) Second, the CIE of the proprietor then determines the remaining ‘all loss fractions’ (ALF) as a percentage and volume, where ALF% = (1–CIE%) and ALF (hm^3) = (FIW*ALF%).

In Stage 5A, the following five agro-hydrology ratios of the proprietor require user-defined inputs:

1) The proprietor CIE%, recalling this is already set in Stage 2 above.
2) The proprietor non-beneficial consumption (PrNBC, %).
3) The proprietor non-recovered fraction (PrNRF, %).
4) The reused recovered fraction (RRF, %).
5) The unused recovered fraction (URF, %).

In Stage 5B, the following three ratios require definition or can be calculated:

6) The neighbour CIE (NbCIE, %) potentially could be user-defined, but the current version of the model references the average CIE from the proprietor CIE calculations. This means that the distribution of gravity, sprinkler and drip set for the proprietor then applies to the neighbour.
7) The neighbour NBC (NbNBC, %).
8) The neighbour NRF (NbNRF, %).

Recall, the first four ratios apportion the ‘all loss fractions’ and these should add up to 100% for ease of error checking. From the above eight user-defined AgHR ratios, the following volumes are calculated by the model for all T1 and T2 scenarios. By comparing T1 and T2 the change in volume over time are also calculated.

1) The proprietor non-benefical consumption (PrNBC, hm^3).
2) The proprietor non-recovered fraction (PrNRF, hm^3).
3) The reused recovered fraction (RRF, hm^3).
4) The unused recovered fraction (URF, hm^3).
5) The neighbour BC (NbBC, hm^3).
6) The neighbour NBC (NbNBC, hm^3).
7) The neighbour NRF (NbNRF, hm^3).

5.6 Stage 6. The society-nature ratio for sharing the non-depleted water

Stage 6 sets the society-nature ratio (SNR) which splits the non-depleted water (water from the non-withdrawn water and the unused return flows) to nature and society. This calculation is done in Stage 6, while in Stage 10, these volumes are added to the core priority allocations to nature to find the total allocation of water for nature.

1) The volume of the non-depleted water (NDW), where NDW = (NWW+URF).
2) A cross-check on the composite volumes that add up to the FIW passing through the proprietor. Thus FIEW should be equal to the sum of PrBC+PrNBC+PrNRF+RRF+URF.

5.7 Stage 7. Aggregate water depletion, aggregate depletion change, max real water saving

Stage 7 calculates the aggregate water depletion, its change and the maximum reduction in aggregate depletion expressed as the realisable water overplus. The aggregate water depletion is the sum of the proprietor beneficial consumption, non-beneficial consumption, non-recovered fraction and the reused recovered fraction (which goes to the neighbour and is all depleted). The calculations are as follows:

1) Aggregate water depletion (AWD) in each of T1 and T2 scenarios; where AWD = PrBC+PrNBC+NRF+RRF
2) Aggregate depletion change (ADC) where ADC = AWDT2 – AWDT1. A negative result means less depletion which is a real water saving and is one of the nexus gains.
3) Maximum realisable water saving (MRWS) is equivalent to the maximum negative ADC from all the scenarios. This can be found manually and is shown in the model by manually highlighting the result in red bold font.

5.8 Stage 8. Redistribution of irrigation water between paracommoners

Stage 8 collates, from information above, the volumes of water accruing to each paracommoner; the proprietor (with a primary and expansion zone), an immediate neighbour (the reuse zone), and society and

nature. Recall water for society and nature via paracommons redistribution is not that derived by purposive allocation in Stage 2. Furthermore, the gain or loss of water by nature and society is a key gauge of the success of re-allocation of water in the paracommons.

1) The water accruing to the proprietor is given by PrBC (hm^3).
2) The water distributed to the neighbour is given by NbBC (hm^3).
3) The water distributed to society is given by the society-nature ratio applied to the non-depleted water, where society (hm^3) = SNR*NDW.
4) The water distributed to nature is given by the society-nature ratio applied to the non-depleted water, where Water for Nature (hm^3) = (1–SNR)*NDW.
5) The 'all depleted losses' (ADL) that are not attributed to any paracommoner (although they occur within both the proprietor and neighbour) is given by NRF + NBC.
6) Comparing T1 and T2 allow the changes in volumetric distributions to be calculated. These are positive when a paracommoner gains water, and negative when the paracommoner loses water. The calculation is as follows; volume change = T2 volume – T1 volume.
7) The volumes shared between the paracommoners (and the ADL) are recalculated as a percentage of the available basin supply (ABS).
8) Two cross-checks are then performed: 1) The sum of the percentage shares to each paracommoner and ADL combined, must add up to 100%. 2) The sum of the volumes must add up to the ABS which visually can be checked to be the same as ABS at the top of the Stage 2 above.
9) The model ascertains two measures of how equitable the paracommoner redistributions have been by seeing how much water is relatively beneficially consumed by the proprietor. First, the percentage of the paracommoner water that is apportioned to the proprietor is calculated. This is = PrIBC/(PrIBC+NbIBC+Soc+Nat). Second, the change in this percentage is calculated by subtracting the percentage in the Baseline T1 scenario from the percentage in the given T2 scenario.
10) The number of people potentially supplied by society's water redistribution can be calculated this by dividing by the gross per capita daily water allowance, correcting for 365 days.

5.9 Stage 9. Derive other metrics

The calculations in Stage 9 start by referencing the depth equivalent net irrigation requirement (NIR) from the NIR in Stage 2 above. This figure applies to both proprietor and neighbour (though for the latter it is an approximation because the neighbour BC is a function of the neighbour CIE applied to the RRF). Restating the NIR clarifies that this figure is employed to calculate the final irrigation areas and total BC. An area-depth-volume correction is also applied knowing 1 mm of water on 1 hectare is 10 cubic metres, and that one cubic hectometre contains a million (1,000,000) cubic metres.

The total zone area (TZA) needs to be calculated because it differs from the 'proprietor starting area' for two main reasons:

- The final proprietor area (PrFA) differs from the starting proprietor area (PrSA) because the starting area for each scenario defines the required water withdrawal (RWW). However, the actual final water withdrawal is controlled by the FFW RULE. This rule may override the RWW volume to dictate that, instead of the required withdrawal, the T1 Baseline water withdrawal (BWW) enters the proprietor/primary zone. Since the T1 BWW is usually larger than T2 RWW (due to water conservation measures in T2), the larger BWW, combined with a smaller T2 NIR and GIR, grows the T2 final area that can be irrigated. This T2 final proprietor area is a combination of the primary proprietor zone area and the expansion zone area (= proprietor expansion area). Thus even though T2 starting areas for the proprietor may be set by user choice and input, the final area still needs to be calculated.
- The neighbour final area (NbFA) is added to the proprietor final area to find the final total irrigated area (TFIA). Recall the neighbour area is not entered as a starting area and is a function of several factors occurring in earlier Stages above; a) the changing final water withdrawn (FWW); b) the changes in the percentages and volume of water that is the reused recovered fraction (RRF); and c) the changing NbCIE%.

With regards to the final areas, note the following:

- The difference between the proprietor starting area (PrSA) and the final proprietor area (PrFA) in each T2 scenario reveals how the proprietor area can expand by; a) cutting its field application depths of application (seen in a lower NIR); and b) continuing with the baseline withdrawal. Thus the proprietor expansion area (PrEA) = (PrFA–PrSA).
- Note the PrEA is not the same as comparing T2 final total zone irrigated areas with T1 final total zone irrigated area because these include the neighbour areas which change.
- The total final area is important as this generates accurate aggregate results for the indicators in Stage 7 described below.

There are no user-defined independent inputs in Stage 9, and therefore all calculations are performed by the model:

1) The proprietor final area (PrFA) is the volume of the PrBC divided by the PrBC (= NIR) as a mm depth equivalent corrected for area-depth-volume, where PrFA = PrBC/NIR*(area-depth-volume correction).
2) The change in proprietor area from the baseline scenario to the given scenario is calculated for all T2 scenarios, and is given in hectares and as a percentage change.
3) The proprietor expansion area (PrEA) is the difference between the final and starting areas for the proprietor, where (PrEA) = (PrFA–PrSA).
4) Neighbour final area (NbFA) is the NbBC (as a volume) divided by the NIR as a mm depth equivalent, corrected for area-depth-volume.
5) The change in neighbour area from the baseline scenario to the new scenario is calculated for T2 scenarios, given in hectares and as a percentage change.
6) Total zone irrigated area (TZA) is the sum of the PrFA and NbFA for each scenario.
7) The change in total area from the baseline scenario to the given scenario is calculated for T2 scenarios, given in hectares and as a percentage change.
8) The total irrigation BC (TIBC) is the sum of the PrBC and NbBC (in hm^3).
9) The change in total irrigation BC in each T2 scenario from the baseline scenario is calculated for T2 scenarios, given in volume and as a percentage change.
10) The 'proprietor other water beneficial consumption' (PrOWBC) is the beneficial consumption of water by crops drawing on local water given by effective rainfall and the informal supplementary water (which is not part of formal irrig withdrawals).
11) The 'neighbour other water beneficial consumption' (NbOWBC) is the OWBC in the neighbouring system.
12) Total zone other water beneficial consumption is then computed from the sum of the proprietor and neighbour other water BC.
13) The change in 'total zone other water beneficial consumption' (TZOWBCC) in volume and % change is then computed.
14) The 'total zone crop BC' (TZCBC) is then the sum of 'total zone irrigated BC' and 'total zone other water BC'.
15) The volumetric and % change in 'total zone crop BC' (TZCBCC) is then computed as the difference between TZCBC in T1 and T2.
16) The proprietor effective irrigation efficiency (PrEIE%) applies to the proprietor system only, where (PrEIE%) = (PrBC)/(PrBC+PrNRF+PrNBC).
17) The aggregate effective irrigation efficiency applies across the whole system of both proprietor and neighbour. Thus AEIE% = (PrBC+NbBC)/AWD.
18) Aggregate depletion impact on total basin supply, is a measure of the impact of aggregate water depletion by irrigation as a percentage of the total basin supply, thus ADITS% = AWD/TBS.
19) Aggregate depletion impact on available basin supply, is a measure of the impact of depletion by irrigation as a percentage of the available basin supply, thus ADIAS% = AWD/ABS.

5.10 Stage 10. Five key dispositions from allocation and conservation

Stage 10 computes five key dispositions arising from both priority allocation of basin supply and paracommoner distribution of withdrawals/non-withdrawals also from total basin supply. The key dispositions in cubic hectometres (= million cubic metres) are:-

1. Total zone beneficial consumption (TZIBC)
2. Total society process consumption (TZPC) from the total of priority allocation and from the non-withdrawn and non-depleted water
3. Total water for nature (WfN) from both priority allocation and from non-withdrawn and non-depleted water
4. Society non-recovered flows/fraction (SNRF)
5. Total zone irrigation depleted losses (TZDL)

Stage 10 then calculates changes to water for nature which is one of the four nexus gains metrics. Changes record the percentage points difference moving from T1 to T2 scenarios in the total volume of water for nature as a percentage of the total basin supply. Stage 10 thus produces important information for the pie-charts given in the worksheet '***Allocation piecharts'***, with the only difference being that the total zone BC is split into the proprietor's and neighbour's BC.

5.11 Stage 11. Pareto-checks on crop production

Stage 11 produces a key nexus metric, the determination of change in the total zone crop production (TZCP). This is derived from the total zone (aggregate) beneficial consumption that is the sum of irrigation application BC (TIBC) and other water beneficial consumption (TZOWBC). It then uses the water use efficiency (water productivity) of a given crop (e.g. maize) in tonnes crop per million cubic metres (t/hm^3) multiplied by the total BC to derive crop production in tonnes or kilotonnes. The nexus gain is when total zone crop production change (TZCPC, derived by comparing T2 TZCP against T1 TZCP) is positive and a nexus reversal is when TZCPC is negative (T2 TZCP<T1 TZCP).

5.12 Stages 12A to 12D. Determine changes to energy requirements

Stage 12 determines the energy required to withdraw water (if from boreholes) and to pump it to fields through the conveyance and distribution network. These calculations utilise standard pressurised pump calculations. There are four sub-stages 12A to 12D as described below:

Stage 12A. Area distribution of three types of irrigation technology. This stage draws on information inserted by the modeller into Stage 3 to calculate the percentage and hectares covered by each type of irrigation technology; gravity, sprinkler and drip. This division into different technology types is critical because of the differences in operating pressure between the three; gravity irrigation uses no pressure (energy) to distribute water, sprinkler is a high-pressure piped system and drip is a low-pressure piped system. In addition to the operating pressure, the lift height in metres (from the aquifer depth to the surface) can be manually inserted.

Stage 12B. Gravity irrigation agronomy to calculate pumped borehole volume. This stage calculates the borehole volumes and subsequent pressures if gravity irrigation must first draw that water from an aquifer. Recall there is no distribution pressure or energy used because gravity distributes the water through open channels. Calculations of lift flow rates, pressures, and therefore energy requirements, take into account the operation of the gravity system. For example if the system is run 24 hours a day, and is supplied by many small pumps, then each pump will have a different energy requirement than say if the system is run 12 hours a day from only a few larger pumps.

Stage 12C. Sprinkler and drip irrigation agronomy to calculate pumped volumes. This stage calculates the volumes and flow rates for both sprinkler and drip.

Stage 12D. Gravity, sprinkler and drip energy requirements. This stage uses the flow rates from Stages 12A to 12C to calculate the energy requirements of the pumps to irrigate within each type of technology.

5.13 Stage 13A to 13D. Carbon dioxide emissions and CO_2 savings

Stage 13 utilises the energy requirements for each type of irrigation technology computed in Stage 12 to derive the carbon dioxide emissions for the total aggregate zone of irrigation. A savings in carbon emissions becomes a nexus gain (if the emissions decline from T1 to T2) and a nexus reversal occurs if emissions increase from T1 to T2. The following steps generate these calculations:

13A Emissions for each type of irrigation technology prior to correction for use of renewables

1. First we have to convert the energy in kWh to the energy in diesel from; diesel kg/kWh = 0.215.
2. The second referenced conversion factor is to derive tonnes of CO2 for each kg of diesel used, from t CO2/kg diesel = 0.002639.
3. For each of the four scenarios, the model converts the total energy demand from Stage 12 to derive four metrics. The four metrics are; gravity irrigation potential carbon emissions (GIPCE); sprinkler irrigation potential carbon emissions (SIPCE); drip irrigation potential carbon emissions (DIPCE) and total zone potential carbon emissions, (TZPCE). This allows the model to know the potential carbon emissions if all energy for irrigation were derived from carbon/fossil fuels. This step is prior to the correction for the use of renewable energy to run the irrigated area – see next.

13B Actual emissions corrected for renewable energy share

1. For each scenario the modeller/user inputs the renewable energy share (RES) as percentage of energy derived from renewables (taken to be carbon-free, which includes nuclear power). This figure applies to all irrigation technology types. For example, a figure of 25% means 25% of emissions from the previous step are taken out.
2. The model then calculates the actual total zone carbon emissions from the previous two steps whereby the TZCE = TZPCE * (1 – RES).
3. The model can then calculate the nexus gains metric of the change in carbon emissions moving from T1 to T2, noting that an increase in emissions over time is a nexus reversal and a drop in emissions in T2 compared to T1 is a nexus gain.

13C Emissions metrics no correction for use of renewable/carbon free energy

This step computes, for each of the three types of irrigation technology, two important carbon emissions metrics but with no correction for renewables. This produces six results for each scenario:

- Gravity irrigation potential emissions per ha (GIPCEha)
- Sprinkler irrigation potential emissions per ha (SIPCEha)
- Drip irrigation potential emissions per ha (DIPCEha)
- Gravity irrig potential emissions per m3 withdrawn (GIPCEm3)
- Sprinkler potential emissions per m3 withdrawn (SIPCEm3)
- Drip irrig potential emissions per m3 withdrawn (DIPCEm3)

13D Emissions metrics corrected for using renewables

Stage 13D reproduces the same six metrics for each scenario, but corrected for the use of renewable energy. These are:-

- Gravity irrigation carbon emissions per ha (GICEha)
- Sprinkler irrigation carbon emissions per ha (SICEha)
- Drip irrigation carbon emissions per ha (DICEha)
- Gravity irrig C emissions per m3 withdrawn (GICEm3)
- Sprinkler irrig C emissions per m3 withdrawn (SICEm3)
- Drip irrig C emissions per m3 withdrawn (DICEm3)

From these calculations we can see that the CO2 emissions of irrigation (kg CO_2/m^3) fall within the range given by the literature (Rothausen and Conway, 2011) of approximately 0.1 to 1.5 kg CO_2/m^3 depending on the type of irrigation technology and lift height.

5.14 Further cross-checks

This small stage currently performs a cross-check on the preceding stages. It compares the 'total gross applied from three irrigation tech' with the 'final irrigation withdrawn' as these should be equal.

6. Stage 14 nexus gains results and graphs

The worksheet '***Stage 14 Nexus metrics***' contains the key nexus results and two graphs of the four nexus gains. It starts by reproducing for each of the four scenarios the four underlying metrics that become the basis for computing the gains. The four underlying metrics are:

1. Aggregate irrigation depletion given by volume (where MCM = hm^3)
2. Crop production given by kilotonnes (kt)
3. Energy carbon emissions given by kilotonnes (kt)
4. Water for nature % of total basin supply

The next part then reproduces the *change* in the four metrics moving from T1 to T2.

1. Aggregate depletion change (hm^3)
2. Change in crop production given by kilotonnes (kt)
3. Change in energy carbon emissions given by kilotonnes (kt)
4. Change in water for nature % of total basin supply, given by the percentage points change

The nexus gains are then recalculated (if necessary) and presented so that a gain is seen as normatively good. For example this is where a reduction in aggregate water depletion is seen as a positive nexus gain.

1. Real water savings. Gains (+) occur when savings occur meaning when ADC decreases (when T2 aggregate depletion is less than T1 aggregate depletion. Reversals (-) occur when ADC increases.
2. Crop production. Gains (+) occur when crop production in T2 is greater than T1. Reversals (-) occur when T2 crop production is less than T1 crop production.
3. Carbon emission savings. Gains (+) occur when emissions in T2 are less than T1 emissions. Reversals (-) occur when T2 emissions are larger than T1 emissions.
4. Water for Nature. Gains (+) occur when T2 % of water for nature is the same as or greater than T1 % of water for nature. Reversals (-) occur when T2 water for nature is less than T1 water for nature.

The volumetric/tonnage nexus gains are also computed for the three T2 scenarios as percentage changes:

1. Real water savings; % gains (+) or reversals (-)
2. Crop production; % gains (+) or reversals (-)
3. Carbon emission savings; % gains (+) or reversals (-)
4. Nature water; % points gains (+) or reversals (-)

Nexus gains graphs

The worksheet '***Nexus gains graphs'*** is specifically devoted to the graphing of the key nexus results. The two graphs presented in the main paper are shown here as **Figures 5 and 6**. The following points apply:-

1. There are three graphs in this worksheet but only two were used in the journal article.
2. The colour coding has been carefully selected to follow previous colours: light blue for aggregate irrigation depletion, green for crop production, red for carbon emissions and navy blue for water for nature.
3. The authors took the decision that the graphs of the nexus gains should be reproducible using Excel without having to export results to other software or to use other Add-In software.
4. The three graphs should be read together. Taken in isolation one graph (e.g. of the percentage change) may generate a false picture of the extent of gains and reversals. Recall, only scenarios 2, 3 and 4 provide the nexus gains graphs because these T2 scenarios compare changes against the results of the T1 scenario.

Figure 5. Total water depletion, crop production, carbon and nature for four scenarios

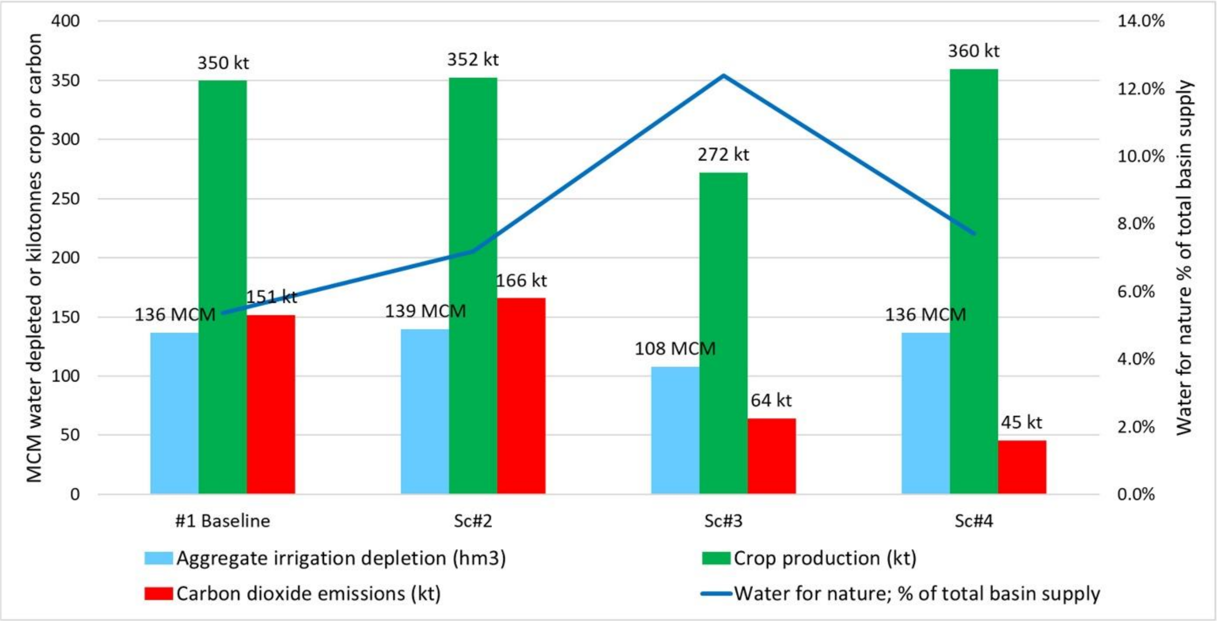

Figure 6. Change in water depletion, crop production, carbon and nature for T2 scenarios

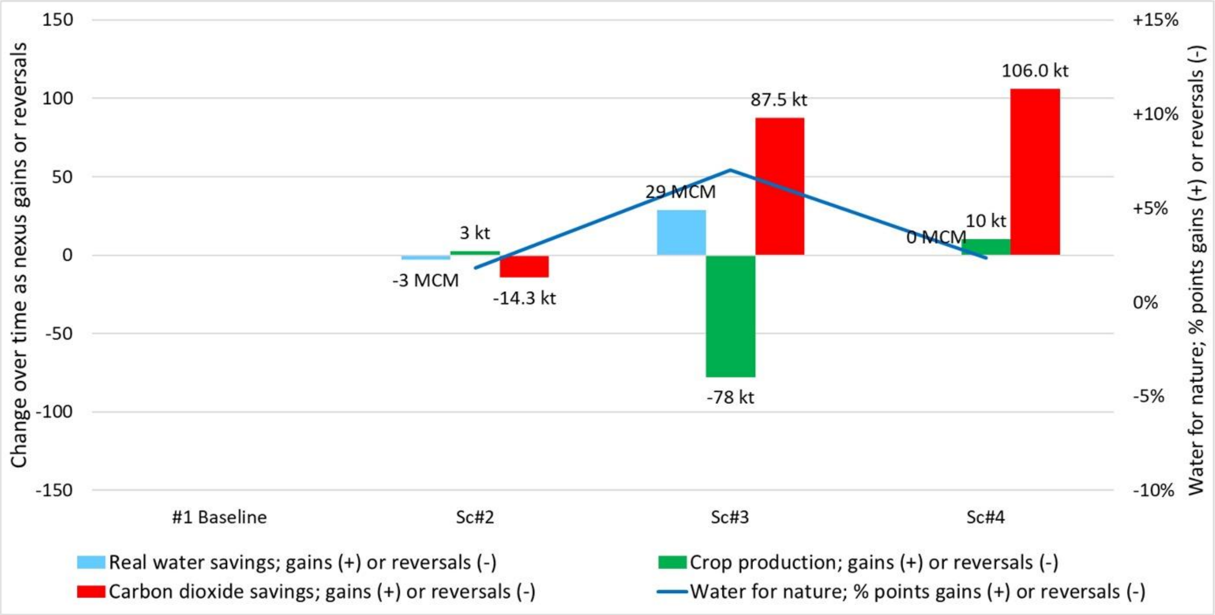

7. Water allocation pie charts

The worksheet '***Piecharts & table 6'*** presents the final water allocations to the six key dispositions of water consumption. These are derived by summing the priority water allocations (and their effects on non-recovered water from society) and the water redistributions through irrigation withdrawals, non-withdrawals and their changing management. The six disposition or fractions are:-

1. Proprietor irrigation beneficial consumption (PrIBC)
2. Neighbour irrigation beneficial consumption (NbIBC)
3. Society process consumption (SPC)
4. Water for Nature (WfN)

5. Society non-recovered fraction (SNRF)
6. Total zone depleted losses (TDZL)

The results (for the AZ Basin) are presented graphically in **Figure 7** and in **Table 4** (Table 6 of the main paper).

Table 4. Six final water disposition from priority water allocation and water conservation

Scenarios	#1 Baseline		Sc#2		Sc#3		Sc#4	
Units	hm^3	%	hm^3	%	hm^3	%	hm^3	%
Proprietor IBC	98.7	27%	119.4	30%	92.2	23%	124.6	31%
Neighbour IBC	10.7	3%	6.9	2%	5.3	1%	4.4	1%
Society process cons.	78	21%	114	29%	125	31%	115	29%
Water for nature	19.7	5%	28.7	7%	49.4	12%	30.7	8%
Society non-recover.	133	36%	116	29%	116	29%	116	29%
Irrig. depl. losses	27.0	7%	13.2	3%	10.2	3%	7.4	2%
Total of fractions	367.0	100%	398.1	1.0	398.1	1.0	398.1	1.0

Figure 7. Final water allocations from priority allocation and paracommons redistribution

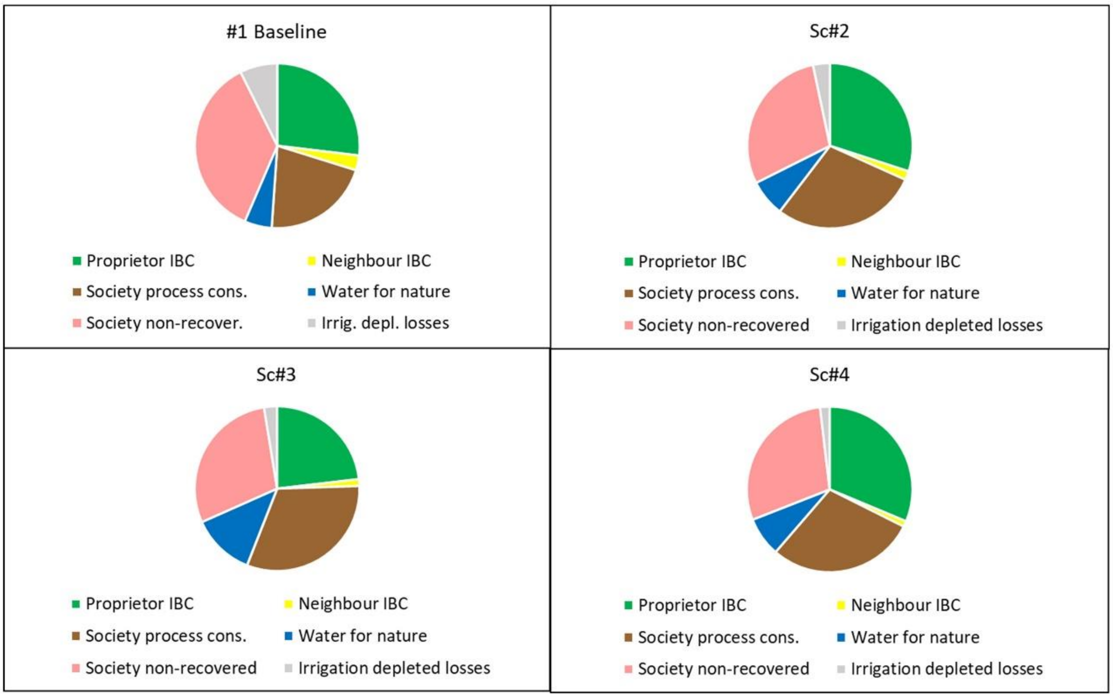

8. Water distribution diagram

In worksheet ‘***Redistrib 4 figs’***, the model generates a water distribution diagram for all four scenarios. This distribution is a function of both a) the purposive water allocation decisions taken in Stage 2 and b) the paracommons water distributions calculated in Stages 8 and 10. The figure, reproduced in **Figure 8,** takes the opportunity of giving the key metrics that underpin the nexus gains.

Figure 8. The water distribution output from the Excel iGains4Gains model

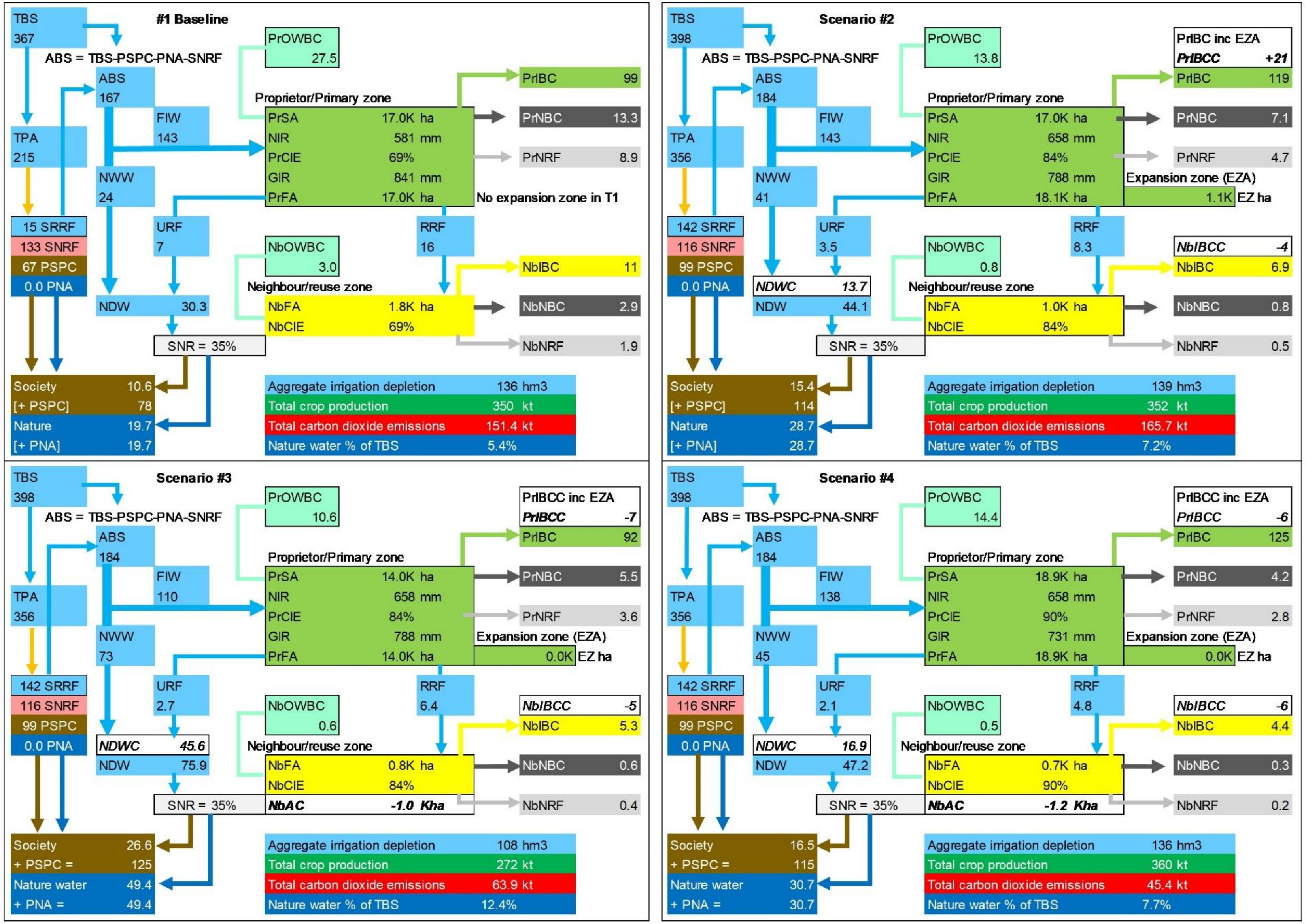

9. Data entry for the AZ Basin Case Study

9.1 Introduction

The AZ Basin in the north-western part of Jordan is part of the larger Jordan River Basin (**Figure 9**). The AZ basin covers an area of approximately 4,100 km^2, with 93% within Jordan and the remaining 7% within Syria. The basin is naturally characterized by an arid to semi-arid climate with limited water resources (the mean annual rainfall is approximately 200 mm). It hosts over 60% of Jordan's population, 80% of its industries, and significant agricultural activities (Al-Omari et al., 2013). Given the high demand from various users and limited water availability, the basin is currently experiencing the consequences of extensive groundwater over-extraction, evidenced by the rapid decline in groundwater levels, with depths in some areas approaching 500 meters (MWI, 2015).

The Amman-Zarqa basin relies primarily on internal groundwater resources and imports from neighbouring basins to meet its water supply needs. The Zarqa River, the basin's main surface watercourse, serves primarily as a carrier for treated wastewater generated within the basin. This treated wastewater is transported via the river for irrigation in the Jordan Valley. Surface water use within the basin is limited to small areas under specific agreements with farmers, allowing only 10% of the river's flow to be utilized locally. The remaining mixed flow is designated for irrigation purposes outside the basin.

Figure 9. The Amman-Zarqa (AZ) river basin

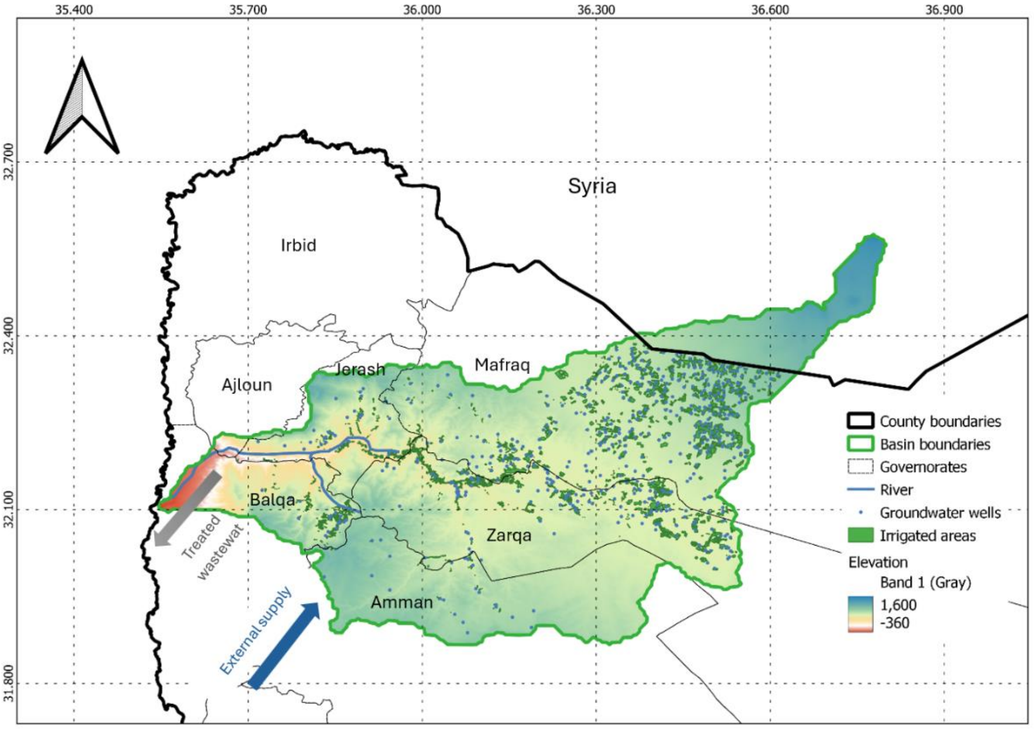

We selected the Amman-Zarqa (AZ) basin as our case study to test and develop iGains4Gains because it faces significant water allocation and irrigation management challenges, which the model is well positioned to answer. These challenges arise from a rapidly changing semi-arid climate, coupled with significant population and economic growth, and persistent over-abstractions of water for irrigation. Combined, these factors exacerbate water scarcity and intensify the complexity of water allocation decisions in the basin.

Over the past two decades, water use efficiency measures, particularly in irrigated agriculture have been a major component of Jordan's efforts to address increasing water scarcity and demand. The efficiency-oriented approach has been further emphasized in Jordan's National Water Strategies for 2016–2023 and 2024–2040 (MWI, 2016; 2023). Significant efforts have been made to improve irrigation efficiency at the field scale, particularly in the Mafraq highlands, a major irrigated area within the AZ basin (Osorio-Cortes,

2022). Although these interventions have demonstrated field-scale water savings through monitoring (Amdar and Elmahdi, 2021), their impacts on basin hydrology and broader nexus outcomes remain unclear. Understanding these effects is important for evaluating the long-term consequences and achieving a balanced approach to water management.

The iGain4Gains model is particularly relevant in this case as it offers a structured approach to understanding the intricate dynamics between available water supply and demand where irrigation is a major consumer of water. Thus, the model supports evaluation of the potential for water savings in irrigated agriculture in the basin and the exploration of choices for reallocating water while monitoring the hydrological impacts and broader nexus implications of reallocation decisions Another pragmatic reason for selecting the AZ basin stems from the availability of key data covering most of the model inputs.

9.2 Establishing future scenarios

To reveal how iGain4Gains calculates nexus changes requires the development of future T2 scenarios, which can be compared to the T1 baseline case. Although the three future scenarios are set for this paper, other model users applying the model to the AZ case study or other basins may select their own future scenarios. The objectives for each of the three future scenarios of the AZ basin are given below. The selected scenarios in this paper explore how changes in irrigation efficiency and irrigated area impact nexus outcomes under climate change. Some variables were chosen based on available data, particularly those related to basin supply (Stage 2) and evapotranspiration changes under climate change (Stage 3), further explained in section 3.3. However, the remaining input variables were selected for their ability to demonstrate the working of the model and trade-offs in nexus gains, and to promote dialogue, rather than to present accurately forecasted and officially recognised future goals. For example, there is no official target for delivering water for nature, which also recognises how politically unpalatable this is in this populous, arid and over-extended basin. Similarly, there are no goals or policies addressing specific changes in irrigated areas required under future climate change. However, in all three future scenarios, irrigation efficiency increases.

The aim of scenario 2 is to show an increase in both water consumption by irrigation and total crop production, higher carbon emissions and a minor gain in water for nature. The rebounds in water consumption and carbon emissions are driven by increases in irrigation efficiency but with no complementary reduction in T2 irrigation withdrawals or additional use of renewables.

Scenario 3 applies a higher efficiency, cuts the irrigated area and imposes a required water withdrawal (not the baseline withdrawal) to derive real water savings for nature. The cut in irrigation sees crop production drop by nearly a third, but this cut, plus greater use of renewable energy, delivers considerable carbon savings. Scenario 3 sees the highest amount of water provided to nature.

Scenario 4 uses a higher irrigation efficiency, applies the required irrigation withdrawal (RIW) as the withdrawal rule, and sets a goal-seek solution for its starting irrigated area to create no change in water consumption (real water savings = 0.0). Compared to the baseline, this scenario delivers gains in crop production, considerable savings in carbon emissions, and slightly more of total basin supply to nature.

9.3 Setting out the supply-side and purposive water allocations

Table 5 summarizes the data used to quantify the AZ basin supply and priority allocation under the baseline scenario (T1), and three potential future scenarios under climate change (Sc 2, Sc 3, Sc 4). Data utilized to develop the T1 scenario were taken from Amdar et al., (2024) who developed the annual water budget for the AZ basin based on ground observations sourced from the Ministry of Water and Irrigation (MWI) and a soil water balance model for the hydrological year 2020 (September 2019 to August 2020). The data and decisions behind them are described below.

Table 5. Inputs for determining basin supplies and priority allocations for the AZ Basin

Scenario		T1	Sc 2	Sc 3	Sc 4
**Supply sources**					
Internal basin supply (renewable groundwater)	hm3	87	73	73	73
Internal basin supply (renewable surface water)	hm3	25	21	21	21
External basin supply	hm3	165	134	134	134
Large-scale dams supply	hm3	0	0	0	0
Fossil water supply	hm3	90	50	50	50
Desalinisation supply	hm3	0	120	120	120
Total basin supply (& change)	hm3	367	398	398	398
**Municipal demand calculations**					
Population (millions)	millions	5.5	8.5	8.5	8.5
Gross per capita daily water use	l/c/day	87	100	100	100
Correction for non-revenue water losses	%	47%	25%	25%	25%
Net per capita daily water use	l/c/day	46	75	75	75
**Priority allocations non-agric**					
Priority domestic allocation (withdrawal)	hm3	174	310	310	310
Priority tourism allocation (withdrawal)	hm3	1	1	1	1
Priority industrial allocation (withdrawal)	hm3	40	45	45	45
Priority society allocation (withdrawal)	hm3	215	356	356	356
Priority water for nature allocation (net)	hm3	0	0	0	0
Total priority allocation (& change)	hm3	215	356	356	356
**Water consumption from priority allocations**					
Domestic process consumption	%	20%	20%	20%	20%
Tourism process consumption	%	80%	80%	80%	80%
Industry process consumption	%	80%	80%	80%	80%
Priority society process consumed (net use)	hm3	67	99	99	99
**Return flows from priority allocation**					
Society return flows	hm3	148	257	257	257
Society recovered return fraction (available)	%	10%	55%	55%	55%
Society recovered return fraction (available)	hm3	15	142	142	142
Society non-recovered fraction	hm3	133	116	116	116
Available basin supply (& change)	hm3	167	184	184	184

- ***Supply sources*** include; 1) internal renewable groundwater supply estimated at 87 hm^3/year, which aligns with the safe yield of the basin's aquifers (MWI, 2015); 2) surface water from the Zarqa River of 25 hm^3/year (MWI, 2023); 3) to meet the high demand, the basin further receives an external supply totalling 165 hm^3/year, primarily piped water from the Disi fossil aquifer located in Southern Jordan to support municipal supply; and 3) an additional 90 hm^3/year of water sourced from internal fossil water reserves within the basin[1]. Collectively, these resources provide 367 hm^3/year of water for the basin.
- ***Municipal water supply:*** the estimated water supply is 87 l/c/day (MWI, 2023). However, due to the significant losses in municipal networks, which amount to 47% (MWI, 2023), the actual net water supply could be as low as 46 l/c/day.

[1] Fossil water is estimated as the difference between total water abstractions in the basin and the aquifers' annual safe yield.

- ***Priority allocation in the basin*** encompasses municipal, industrial, and tourism sectors. Municipal supply, estimated based on an 87 l/c/day demand, totals 174 hm^3/year. Industrial water allocation is 40 hm^3/year, and tourism allocation is less than 1 hm^3/year. This brings the total priority non-agricultural water allocation to 215 hm^3/year.
- ***Priority water for nature allocation*** is set at 0.0 hm^3/year under all scenarios. However, water is provided to nature via return flows and savings from irrigation.
- ***Water consumption from priority allocation*** is estimated at 20%, 80% and 80% of water allocated to the domestic, tourism and industrial sectors, respectively, equivalent to a collective net use of 67 hm^3/year.
- ***Return flows from priority allocation***; the remaining water allocated to priority sectors but not consumed is 148 hm^3/year, equivalent to the volume of treated wastewater (TWW) generated within the basin. The return flows are calculated by iterating the 'Priority society process consumed (net use)' to a level that best reflects known conditions in the basin[2]. Of this amount, 10% is recovered for restricted irrigation along the Zarqa River (Al-Bakri et al., 2016), while the remaining return flow, referred to in our model as the 'society non-recovered fraction', is transferred via the Zarqa River to the Jordan Valley for irrigation (which is not included in the model).

Concerning future scenarios Sc 2, Sc 3, and Sc 4 variables in the model, projections on the basin's water resources under climate change by 2050 were sourced from Jordan's 4th National Communication Report (UNDP and Ministry of Environment, 2022). The report provides detailed projections of future water supplies in the AZ basin described in the following points:

- ***Supply sources***: the basin is projected to experience an average reduction of 16% in recharge by 2050 compared to the baseline period of 1990 to 2020. This reduction could decrease the internal renewable groundwater supply to 73 hm^3/year and fossil water supply to 50 hm^3/year by 2050.Similarly, surface water supply is expected to decline to 21 hm^3/year Given the anticipated downward trend in water availability in Jordan, supply from external sources is also projected to decrease by 20% to 134 hm^3/year. To cover the future water demand gap, the government has initiated the National Desalination Project. This project will provide around 300 hm^3/year of desalinated seawater by 2040 (MWI, 2023). Our model assumes approximately 120 hm^3/year, given that not all of this will be achieved or delivered entirely to the AZ basin. Collectively, these resources could raise the basin supply to 398 hm^3/year by 2050.
- ***Municipal water supply***: with an anticipated future gross demand of 100 l/c/day, and accounting for water losses in municipal networks of 25%, the anticipated net per capita supply in the municipal sector is projected to be 75 l/c/day.
- ***Priority allocation*** in the basin is expected to increase to 356 hm^3/year due to population growth reaching approximately 8.5 million. Allocation for industries is projected to rise to 45 hm^3/year, while the tourism sector's allocation will remain slightly less than 1.0 hm^3/year.
- ***Process water consumption from priority allocation*** was estimated at 20%, 80% and 80% of water supply to the domestic, tourism and industrial sectors, respectively.
- ***Return flows from priority allocation*** were estimated at 257 hm^3/year. Of this, 142 hm^3/year can be recovered within the basin and used for expanding irrigation with treated wastewater along the Zarqa River. The remaining flows via the Zarqa River out of the basin to the Jordan Valley, is used by irrigation.

Based on the data explained above, the remaining available basin supply after allocation to priority users was estimated at 167 hm^3/year under the baseline T1 scenario, and increases to 184 hm^3/year in the three future scenarios.

9.4 Establishing the irrigation requirements, efficiency hydrology and withdrawals

In the next step (**Table 6**), we estimated the irrigation water requirement and final irrigation withdrawal for the baseline T1 period using the data below.

[2] However the gauging station downstream of the major urban centres records different values. This is an instance where the modellers have to decide how to judge different hydrological processes and recorded flows.

Table 6. Inputs for irrigation planning and efficiency hydrology

Scenario		T1	Sc 2	Sc 3	Sc 4
Proprietor starting area (= primary zone area)	ha	17000	17000	14000	18930
Reference crop evapotranspiration	mm	1736	1813	1813	1813
Average areal crop factor	coeff.	0.50	0.50	0.50	0.50
Crop evapotranspiration	mm	868	907	907	907
Field ET reduction	coeff.	0.95	0.90	0.90	0.90
Deficit irrigation factor	coeff.	0.90	0.90	0.90	0.90
ETcrop actual	mm	742	734	734	734
Additional beneficial uses	mm	0	0	0	0
Rainfall	mm	202	95	95	95
Correction for effective rainfall	coeff.	0.80	0.80	0.80	0.80
Effective rainfall	mm	162	76	76	76
Informal suppl. water (not part of formal irrig withdrawals)	mm	0	0	0	0
Net irrigation req, depth equiv (from formal withdrawals)	mm	581	658	658	658
Gravity irrigation, % of total zone	%	0%	0%	0%	0%
Sprinkler irrigation, % of total zone	%	10%	10%	10%	0%
Drip irrigation, % of total zone	%	90%	90%	90%	100%
Gravity irrigation farm IE	%	40%	40%	40%	40%
Sprinkler irrigation farm IE	%	60%	70%	70%	70%
Drip irrigation farm IE	%	70%	85%	85%	90%
Average proprietor system CIE (=PZCIE)	%	69%	84%	84%	90%
Conveyance efficiency	%	95%	96%	96%	97%
Unit distribution efficiency	%	95%	96%	96%	97%
Field appl. eff. Set CIE, Ec, Ed so Ea not exceed 100%	%	76%	91%	91%	96%
Gross irrigation requirement, depth equiv	mm	841	788	788	731
Field application depth (& change)	mm	759	727	727	688
Final irrigation withdrawal rule		BIW	BIW	RIW	RIW
Final irrigation withdrawal	hm3	143.0	143.0	110.4	138.5
Final total irrigated area	ha	18845	19191	14809	19593

- ***Irrigated area***, which represents the starting proprietor area *within* the AZ basin, was estimated at approximately 17,000 ha (Shammout et al., 2021). These areas are clustered in the north-eastern part of the basin within the Mafraq governorate. Nearly 85% of this irrigated land is cultivated with orchards such as stone fruits and olives, while the remaining area is used for seasonal vegetables. Most irrigated areas utilise drip irrigation, while fewer farms still use high-flow mini-sprinklers primarily on stone fruits (Al-Raggad and Fraj, 2019).
- ***The reference crop evapotranspiration and rainfall*** over irrigated areas in the basin were derived from the FAO's Water Productivity portal through Open-Access to level 2 remotely sensed data. The average crop evapotranspiration was 1,736 mm/year, while the average rainfall was 202 mm/year in 2020.
- ***The actual crop evapotranspiration***: given the dominance of fruit orchards, their irrigation season commencing from February to November, the average areal crop factor was estimated at 0.5. Considering a field reduction factor of 0.95, and a deficit irrigation factor of 0.9, the average actual crop evapotranspiration in this region was calculated at 742 mm/year. This value was validated against that estimated for this region between 2017 and 2019 at between 716-722 mm/year (Al-Bakri et al., 2023).
- ***The net irrigation requirements*** were thus calculated at 581 mm/year, assuming a contribution of 162 mm/year of effective rainfall, where 742 – 162 = 581.

- Considering the weighted average efficiency per irrigation system type and conveyance efficiency, ***the field application depth*** was estimated at 759 mm/year under the baseline scenario.
- The above inputs resulted in a gross irrigation requirement of 841 mm, and a final irrigation withdrawal (termed the baseline irrigation withdrawal in T1) of 143.0 hm^3.

Also, in **Table 6**, and following the same approach, the field application depths and water withdrawals were estimated under the T2 scenarios (Sc 2, Sc 3, and Sc4) as follows:

- ***Irrigated areas*** were maintained at 17,000 ha under Sc 2, equal to the baseline scenario T1. However, irrigated areas were assumed to decrease to 14,000 ha under Sc 3 and increase to 18930 ha under Sc 4 (derived using Excel's goal-seek to achieve no change in depleted water). Note that the final irrigated areas differ from the proprietor's starting area due to irrigation expansion or reuse.
- ***Average crop evapotranspiration*** is projected to increase by an average of 4% by 2050, from 1,736 mm/year to 1,813 mm/year. However, rainfall is projected to decrease from 202 mm/year in 2020 to an average of approximately 95 mm/year by 2050 (UNDP and Ministry of Environment, 2022). Accordingly, the net irrigation requirements increase from 581 mm/year in the baseline case to 658 mm/year for all three future scenarios.
- ***Improvements in farm irrigation efficiency*** were assumed, taking sprinkler irrigation up to 70% efficiency and drip irrigation to 85% efficiency in Scenarios 2 and 3. This resulted in a new lower average field application depth of 788 mm/year. In Sc 4, drip was applied to 100% of the AZ basin, accompanied by an increased efficiency to 90%, delivering a lower field application depth of 688 mm. These increases in efficiency mean that, even though net irrigation requirements increase due to climate change, gross irrigation requirements decrease from 841 mm/yr in Case 1 to 727 mm/yr for Scenarios 2 and 3 and down to 688 mm/year in Scenario 4.
- *The **final irrigation withdrawal** (FIW)* was set using the baseline withdrawal (BIW) for Scenario 2 and the required irrigation withdrawal (RIW) for Scenarios 3 and 4. This resulted in withdrawals for Scenarios 2, 3 and 4 of 143.0 hm^3, 110.4 hm^3 and 138.5 hm^3 respectively. These withdrawal rules were chosen deliberately to demonstrate different nexus trade-offs.

9.5 Establishing changes in energy use

Table 7 presents the key input selections that drive the changes in energy use, both in terms of energy requirements for irrigation and the proportion of energy sourced from non-GHG renewable sources. Regarding the latter, this is set at 30% in the T1 case (NEPCO, 2022) For Scenario 2 it is maintained at 30%, but is increased in Scenarios 3 and 4 to 65% and 80%, respectively. The lift height from the deep aquifers is set at 300 metres for the baseline, increasing to 330 m in all three future scenarios (MWI and BGR, 2019). It is important to note that there is no gravity-fed field irrigation in all four scenarios, and that all changes occur within sprinkler and drip technologies.

Table 7. Inputs for irrigation energy requirements and use of renewables

Scenario		T1	Sc 2	Sc 3	Sc 4
Sprinkler net depth per irrigation dose	mm	40	40	40	40
Drip net depth per irrigation dose	mm	20	20	20	20
Sprinkler irrigation field app rate per hour	mm/hr	7	7	7	7
Drip irrigation field app rate per hour	mm/hr	2	2	2	2
Sprinkler area per pump	ha	50	50	50	50
Drip area per pump	ha	50	50	50	50
Sprinkler operating pressure	m	45	45	45	45
Drip operating pressure	m	15	15	15	15
Lift height from borehole - sprinkler	m	300	330	330	330
Lift height from borehole - drip	m	300	330	330	330
Renewable energy share - all irrigation types	%	30%	30%	65%	80%

10. References for this manual

Al-Bakri, J.T., D'Urso, G., Calera, A., Abdalhaq, E., Altarawneh, M., Margane, A. (2023) Remote Sensing for Agricultural Water Management in Jordan. Remote Sensing 15, 235.

Al-Bakri, J.T., Shawash, S., Ghanim, A., Abdelkhaleq, R. (2016) Geospatial Techniques for Improved Water Management in Jordan. Water 8, 132.

Al-Omari, A., Al-houri, Z., Al-Weshah, R. (2013) Impact of the As Samra wastewater treatment plant upgrade on the water quality (COD, electrical conductivity, TP, TN) of the Zarqa River. Water Science and Technology 67, 1455-1464.

Al-Raggad, M., Fraj, B.M., (2019) Simulation of the Project Impact on Groundwater Resources Under Scenarios of Adoption of Water Saving Technologies and Practices in Azraq and Mafraq, Jordan: Water Innovation Technologies Project. USAID.

Amdar, N., Elmahdi, A., (2021) Water Accounts Monthly Report for Water Innovation Technologies (WIT). International Water Management Institute and Mercy Corps, USAID, Amman, Jordan.

Amdar, N., Seyoum, S., Al-Bakri, J., Rutten, M., Jewitt, G., Mul, M. (2024) Developing a water budget for the Amman-Zarqa basin using water accounting plus and the pixel-based soil water balance model. Modeling Earth Systems and Environment.

FAO (1999) Crop Evapotranspiration, FAO Irrigation and Drainage Paper No. 56. Food and Agriculture Organisation of the United Nations (FAO), Rome.

Lankford, B.A. (2023) Resolving the paradoxes of irrigation efficiency: Irrigated systems accounting analyses depletion-based water conservation for reallocation. Agricultural Water Management 287, 108437.

Lankford, B.A., Scott, C.A. (2023) The paracommons of competition for resource savings: Irrigation water conservation redistributes water between irrigation, nature, and society. Resources, Conservation and Recycling 198, 107195.

MWI, (2015) Jordan Water Sector Facts and Figures. Ministry of Water and Irrigation, Amman, Jordan.

MWI, (2023) National Water Strategy 2023-2040 – Summary. Ministry of Water and Irrigation, Amman, Jordan.

MWI, BGR, (2019) Groundwater Resource Assessment of Jordan 2017. . Ministry of Water and Irrigation; Bundesanstalt für Geowissenschaften und Rohstoffe, Amman, Jordan, p. 151.

NEPCO, (2022) National Electric Power Company Annual Report 2022, Jordan, p. 112.

Osorio-Cortes, L.E., (2022) Harnessing market systems for water conservation in Jordan. Mercy Corps, USAID, Amman, Jordan.

Pérez-Blanco, C.D., Hrast-Essenfelder, A., Perry, C. (2020) Irrigation Technology and Water Conservation: A Review of the Theory and Evidence. Review of Environmental Economics and Policy 14, 216-239.

Perry, C. (2011) Accounting for water use: Terminology and implications for saving water and increasing production. Agricultural Water Management 98, 1840-1846.

Rothausen, S.G.S.A., Conway, D. (2011) Greenhouse-gas emissions from energy use in the water sector. Nature Clim. Change 1, 210-219.

Seckler, D.W. (1996) The new era of water resources management : from "dry" to "wet" water savings. International Irrigation Management Institute, Colombo, Sri Lanka.

Shammout, M.a.W., Shatanawi, K., Al-Bakri, J., Abualhaija, M.M. (2021) Impact of Land Use/Cover Changes on the Flow of the Zarqa River in Jordan. Journal of Ecological Engineering 22, 40-50.

UNDP, Ministry of Environment, (2022) Jordan's Fourth National Communication Report on Climate Change. United Nations Development Programme.

Willardson, L., Allen, R., Frederiksen, H., (1994) Elimination of irrigation efficiencies, 13th Technical Conference. USCID, Denver, Colorado, pp. 19-22.
